# Rapid Discovery of De Novo Deleterious Mutations in Cattle Enhances the Value of Livestock as Model Species

**DOI:** 10.1038/s41598-017-11523-3

**Published:** 2017-09-13

**Authors:** E. Bourneuf, P. Otz, H. Pausch, V. Jagannathan, P. Michot, C. Grohs, G. Piton, S. Ammermüller, M.-C. Deloche, S. Fritz, H. Leclerc, C. Péchoux, A. Boukadiri, C. Hozé, R. Saintilan, F. Créchet, M. Mosca, D. Segelke, F. Guillaume, S. Bouet, A. Baur, A. Vasilescu, L. Genestout, A. Thomas, A. Allais-Bonnet, D. Rocha, M.-A. Colle, C. Klopp, D. Esquerré, C. Wurmser, K. Flisikowski, H. Schwarzenbacher, J. Burgstaller, M. Brügmann, E. Dietschi, N. Rudolph, M. Freick, S. Barbey, G. Fayolle, C. Danchin-Burge, L. Schibler, B. Bed’Hom, B. J. Hayes, H. D. Daetwyler, R. Fries, D. Boichard, D. Pin, C. Drögemüller, A. Capitan

**Affiliations:** 1 GABI, INRA, AgroParisTech, Université Paris-Saclay, 78350 Jouy-en-Josas, France; 2 LREG, CEA, Université Paris-Saclay, 78350 Jouy-en-Josas, France; 3 Veterinary School of Lyon (VetAgro Sup), Cattle Pathology Unit, Marcy l’Etoile, France; 4 Chair of Animal Breeding, Technische Universitaet Muenchen, Freising-Weihenstephan, Germany; 5 Institute of Genetics, Vetsuisse Faculty, University of Bern, Bremgartenstrasse 109a, 3001 Bern, Switzerland; 6 ALLICE, Paris, France; 7 Institut de l’Elevage, Paris, France; 8 INRA, MIMA2 MET – Equipe Plateformes, Jouy-en-Josas, France; 9 Université de Lyon, Veterinary School of Lyon (VetAgro Sup), UPSP 2011-03-101 Interactions Cellules Environnement, Marcy l’Etoile, France; 10 Vereinigte Informationssysteme Tierhaltung w.V., Verden, Germany; 11 LABOGENA, Jouy-en-Josas, France; 12 Veterinary School of Lyon (VetAgro Sup), Imaging Department, Marcy l’Etoile, France; 13 INRA, UMR703 Animal Pathophysiology and Biotherapy for Muscle and Nervous system Diseases, Atlantic Gene Therapies, Nantes, France; 14 LUNAM University, Oniris, Nantes-Atlantic National College of Veterinary Medicine, Food science and Engineering, Nantes, France; 15 INRA, Sigenae Bioinformatics Group, UR875, 31320 Castanet-Tolosan, France; 16 GenPhySE, Université de Toulouse, INRA, INPT, INP-ENVT, 31320 Castanet Tolosan, France; 17 Chair of Animal Biotechnology, Technische Universität München, Freising-Weihenstephan, Germany; 18 ZuchtData GmbH, Vienna, Austria; 19 Clinic for Ruminants, University of Veterinary Medicine Vienna, Vienna, Austria; 20 Lower Saxony State Office for Consumer Protection and Food Safety, Department of Pathology, Oldenburg, Germany; 21 Landesuntersuchungsanstalt für das Gesundheits- und Veterinärwesen Sachsen, Bahnhofstrasse 60, 04158 Leipzig, Germany; 22 Veterinary Practice Zettlitz, Strasse der Jugend 68, 09306 Zettlitz, Germany; 23 INRA, UE0326, Domaine expérimental du Pin-au-Haras, Exmes, France; 24 UMOTEST, Ceyzeriat, France; 25 AgriBio, Centre for AgriBioscience, Biosciences Research, DEDJTR, Bundoora, Australia; 26 Queensland Alliance for Agriculture and Food Innovation, Centre for Animal Science, The University of Queensland, Brisbane, Australia; 27 School of Applied Systems Biology, La Trobe University, Bundoora, Australia

## Abstract

In humans, the clinical and molecular characterization of sporadic syndromes is often hindered by the small number of patients and the difficulty in developing animal models for severe dominant conditions. Here we show that the availability of large data sets of whole-genome sequences, high-density SNP chip genotypes and extensive recording of phenotype offers an unprecedented opportunity to quickly dissect the genetic architecture of severe dominant conditions in livestock. We report on the identification of seven dominant *de novo* mutations in *CHD7*, *COL1A1*, *COL2A1*, *COPA*, and *MITF* and exploit the structure of cattle populations to describe their clinical consequences and map modifier loci. Moreover, we demonstrate that the emergence of recessive genetic defects can be monitored by detecting *de novo* deleterious mutations in the genome of bulls used for artificial insemination. These results demonstrate the attractiveness of cattle as a model species in the post genomic era, particularly to confirm the genetic aetiology of isolated clinical case reports in humans.

## Introduction

For centuries humans have taken advantage of their apparent similarities with animals to gain insights into their own anatomy, physiology, and diseases. In modern biology, research mainly focuses on a very limited number of species (i.e. laboratory animals) selected for their high reproductive rate, small generation interval and ease of manipulation^1^. The study of laboratory animals has dramatically improved our understanding of the molecular basis of a number of phenotypes, and in particular human genetic disorders, with the characterization of thousands of genes/mutations in the past 30 years.

Nevertheless, the use of laboratory animals has limitations when it comes to the study of certain dominant conditions. For example, rodents, which are the most commonly studied mammals, have undergone a rapid evolution and display an unusual tolerance to haploinsufficiency of transcription factor genes^2^. The absence of genetic variability in inbred strains requires several generations of crossing with other strains to map spontaneous *de novo* mutations. Finally, the most harmful dominant mutations cannot be studied since (i) they result in the death of the carriers before they reach sexual maturity and (ii) the prolificacy of laboratory animals is often not high enough to produce enough affected individuals among the progeny of mosaic parents.

Livestock such as cattle have the potential to be relevant models for these dominant conditions. Cattle have higher effective prolificacy (i.e. through the use of artificial insemination (AI), bulls can have thousands or tens of thousands of offspring), greater genetic diversity, and greater similarity with humans in terms of physiology, body size and longevity. In the past, the costs per animal and lack of genomic tools limited the value of cattle as a model species. Recently there has been a rapid accumulation of whole genome sequences (WGS) of bulls, industry scale SNP chip genotyping data for routine genomic evaluations (with millions of animals genotyped), and comprehensive phenotyping in large well-recorded pedigrees (through industry performance recording). This provides a unique opportunity to speed up the identification of mutations associated with Mendelian diseases in cattle, and makes cattle an attractive model species^3, 4^.

The aim of this study was (i) to identify deleterious *de novo* mutations using WGS data as a primary source of information and (ii) to draw the attention of the scientific community to the opportunities offered by the bovine animal model.

## Results and Discussion

### Rapid discovery of *de novo* deleterious mutations using WGS data

**Figure 1:**
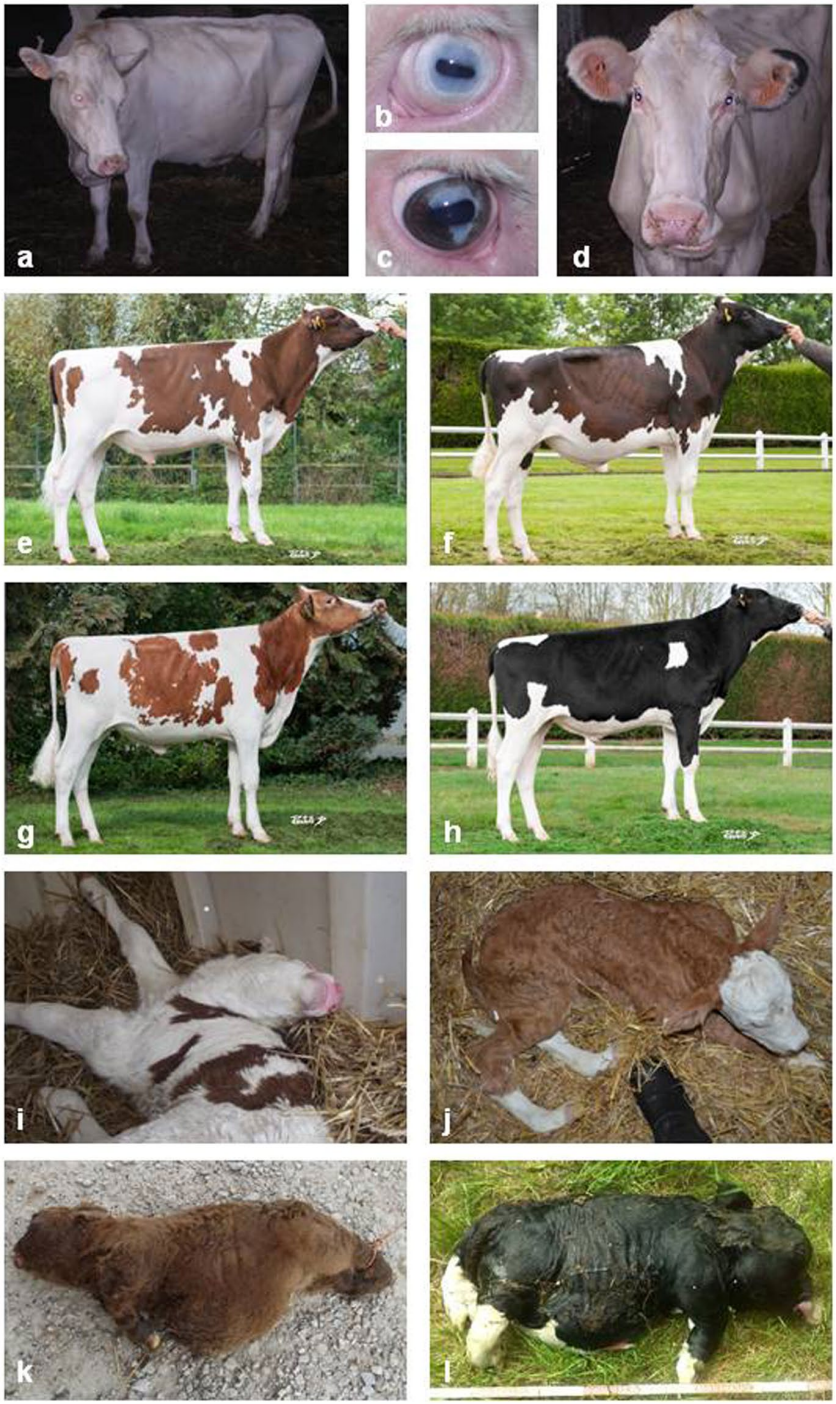
Brief overview of the dominant conditions studied. Glass-Eyed Albino (**a**–**d**) animals trace back to a mutant heifer born in 1994. Clinical features include white coat color (**a**), deafness, and “glass-eyes” (**b**). Partial phenotypic reversion consisting in *heterochromia irides* (**c**) or black spots on the ears (**d**) are sometimes observed. Dominant Red (**e**–**h**) is distinct from the traditional recessive red allele of the Melanocortin-1 receptor (MC1R^e^) found in Holstein^66^. It emerged in 1980 with the birth of a red mutant heifer (MC1R^D/D^ & DR^DR/+^) from parents which were homozygous for the dominant black allele (MC1R^D/D^ & DR^+/+^). In adulthood MC1R^D/D^ & DR^DR/+^ dominant red animals (**e**,**f**) display substantial variations in colour ranging from a light brown which is close to the MC1R^e/e^ & DR^+/+^ recessive red coat (**g**), to a dark brown close to MC1R^D/D^ & DR^+/+^ dominant black coat (**h**). Neurocristopathy (NC) was observed in half of the progeny of a mildly affected Montbéliarde bull. Its main symptoms include hypotonia (**i**), lack of balance and coordination in the days following birth, facial abnormalities, heart defects and growth delay. *Osteogenesis imperfecta* type 2 (OI), is characterized by brittle bones that are prone to fracture (e.g. hind limbs in **j**). It has been reported in 29% of the progeny of an unaffected Fleckvieh bull. Bulldog (BD) calf syndrome is a lethal form of chondrodysplasia characterized by a generalized shortening of long bones (**k**,**l**). BD has been reported in 4% of crossbred calves from a Charolais bull (BD1, **k**), in 21% of purebred calves from a Holstein sire (BD2, **l**), and in an isolated case born from Holstein parents (BD3). Pictures **e–h** were generously provided by the breeding company Origenplus.

We collected samples from affected animals and their healthy relatives for seven autosomal dominant conditions in four cattle breeds: glass-eyed albino (GEA) and dominant red (DR) in Holstein cattle, neurocristopathy (NC) in Montbéliarde cattle, *osteogenesis imperfecta* type 2 (OI) in Fleckvieh cattle, and bulldog calf syndrome (BD1, BD2 and BD3) in a Charolais X Salers cross and in two Holstein pedigrees (Fig. 1; for information on pedigrees see Supplementary Note 1).

We sequenced the genome of one animal per condition at nine to 20.7 × coverage on Illumina HiSeq instruments using paired-end reads (see Methods). We called variants with SAMtools^5^ and filtered them to retain heterozygous polymorphisms that were absent from 1230 control genomes^4^ (Supplementary Table 1) and were predicted to be deleterious to protein function according to Ensembl Variant Effect Predictor annotations^6^. For each of the seven conditions studied, the large number of control genomes enabled us to decrease the number of candidate causal polymorphisms from millions to only one mutation (Table 1, Supplementary Table 2, and Supplementary Fig. 1).

**Table 1:** Results of the Whole Genome Sequencing and filtering approach.

Defect	Coverage; Gaps	Het. variants	Priv. het. variants	Delet. priv. het. variants	Candidate mutation	Human syndromes (MIM #)
Glass-Eyed Albino (GEA)	11.5 x; 1.5%	3.1 × 10^6^	4.4 × 10^3^	3	MITF p.R211del	Tietz syndrome (103500)
Dominant Red (DR)	13.4 x; 1.2%	3.4 × 10^6^	3.2 × 10^3^	3	COPA p.R160C	—
Neurocristopathy (NC)	13.4 x; 0.9%	3.8 × 10^6^	9.7 × 10^3^	15	CHD7 p.K594AfsX29	CHARGE syndrome (214800)
*Osteogenesis imperfecta* type 2(OI)*	20.7 x; 1.1%	3.5 × 10^6^	4.5 × 10^3^	12	COL1A1 p.A1049_P1050delinsS	*Osteogenesis imperfecta* type 2 (166210)
Bulldog Calf Syndrome (BD1)	12.9 x; 1.2%	3.8 × 10^6^	28.4 × 10^3^	29	COL2A1 p.G600D	Achondrogenesis type II (200610)
Bulldog calf syndrome (BD2)**	9.0 x; 1.6%	1.9 × 10^6^	4.2 × 10^3^	28	COL2A1 p.G996S	
Bulldog calf syndrome (BD3)	15.1 x; 1.1% (15.8 x; 1.2% & 16.2 x; 1.4%)	3.2 × 10^6^	9.0 × 10^3^ (200)	9 (1)	COL2A1 p.G720S	

Het. variants: number of heterozygous variants; Priv. het. variants: number of private heterozygous variants; and Delet. priv. het. variants: number of deleterious private heterozygous variants after filtering for variants presents in 1230 control genomes or, between brackets, with 1230 control genomes and both parents. x: unit corresponding to the average number of time that one base pair of the genome is read. Gaps: percentage of the UMD3.1 bovine sequence assembly that is not covered by sequence reads. *: the sequenced animal was the mosaic sire. **: to identify mutations compatible with BD2 syndrome in Holstein cattle, we applied a less stringent quality threshold (quality score = 15 instead of 30) compared to the other defects due to the lower genome coverage (9.0 x) of the BD2 sequencing data (see Methods). Note that for the Holstein GEA, DR, BD2 and BD3 animals, the number of private heterozygous variants is inferior or close to the number of *de novo* mutations which may have accumulated since the creation of this breed considering that approximately 200 mutations accumulated at each generation over 20 generations. This illustrates how the small effective size of the worldwide Holstein population (Ne ~100) combined with the high number of control genomes for this breed (n = 345) enable to capture most of its genetic diversity. In contrast, the elevated number of private heterozygous variants for the Charolais X Salers crossbred calf BD1 reflects the small number of control genomes available for the Salers breed (n = 4).

### Confirmation of the *de novo* nature and validation of the causality of the candidate mutations

Several analyses were conducted to confirm the causality of the candidate mutations and the reliability of our approach.

For GEA, DR and NC, we took advantage of the large pedigrees of livestock populations and mapped the conditions to 1.6-, 4.6- and 0.7-Mb intervals, respectively, using case-control association testing with Illumina BovineSNP50 genotype data (see Methods, Supplementary Table 3). We mined the WGS data of one affected animal per condition and identified only 3, 4 and 2 small private mutations that were compatible with dominant inheritance and located within the GEA, DR and NC associated intervals, respectively. In addition, we performed structural variant detection in the sequence data of case and control animals with Pindel^7^, DELLY^8^, and the Integrative Genomics Viewer (IGV)^9^, and we did not detect any additional candidate mutation within these intervals. We then mined Illumina BovineSNP50 phased genotypes from case relatives and from the large French genomic selection database (231,115 Holstein cattle genotypes and 99,044 Montbéliarde cattle genotypes) to identify unaffected individuals carrying ancestral versions of the disease-associated haplotypes, i.e. without the causative mutations. Finally, we genotyped these specific control individuals and affected animals by PCR and Sanger sequencing for the candidate mutations. We observed that only mutation p.R211del in microphthalmia-associated transcription factor (MITF) for GEA, mutation p.K594AfsX29 in chromodomain-helicase-DNA-binding protein 7 (CHD7) for NC and mutation p.R160C in coatomer protein complex, subunit alpha (COPA) for DR were absent from control animals carrying ancestral versions of the disease-associated haplotypes. Our results confirm the *de novo* nature of these mutations and support their causality (Supplementary Tables 4 and 5).

For OI, BD1 and BD2, analyzing DNA samples extracted from blood and semen with Sanger sequencing and/or pyrosequencing, we demonstrated that the three healthy sires of affected calves were both germline and somatic mosaic for mutations p.A1049_P1050delinsS in Collagen type I alpha 1 chain (COL1A1), and p.G600D and p.G996S in Collagen type II alpha 1 chain (COL2A1), respectively (Supplementary Figs 2–4).

Finally, for BD3, we sequenced the unaffected parents of the bulldog calf and determined that among the putative deleterious variants absent from 1230 control genomes, only mutation COL2A1 p.G720S occurred *de novo* (Table 1, Supplementary Fig. 5).

### Cattle, a reliable animal model for studying human genetic defects

Strikingly, clinical examinations showed perfect genotype-phenotype correlations between affected calves and human patients with mutations affecting the same domains of the same proteins (i.e. for BD1-3 and COL2A1, OI and COL1A1, GEA and MITF, and NC and CHD7), demonstrating that cattle may be a useful model for human genetic diseases.

**Figure 2:**
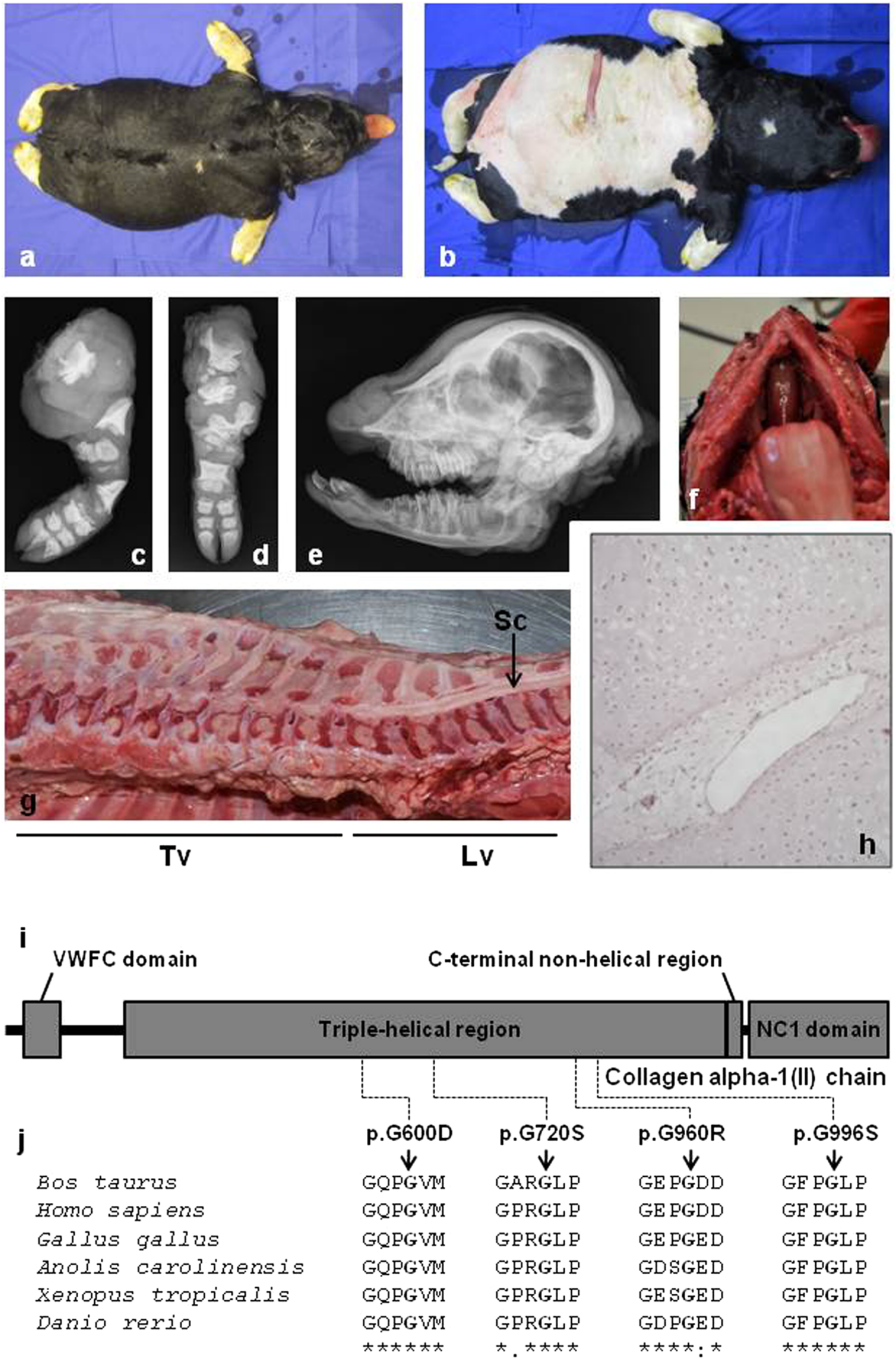
Bulldog calves show perfect genotype-phenotype correlations with humans affected by hypochondrogenesis/achondrogenesis type II. (**a**–**h**) Phenotypic characteristics of a bulldog calf (here the COL2A1 p.G720S/+ calf BD3) which, like humans affected by hypochondrogenesis/achondrogenesis type II, shows abnormal endochondral ossification resulting in a general shortening of long bones. (**a**) dorsal view and (**b**) ventral view. Note the extremely short limbs and short head. Radiographs: (**c**) of the left hind limb and (**d**) the left front limb showing multiple short dysplastic bones, (**e**) of the head demonstrating a ventrally rotated and dysplastic splanchnocranium. (**f**) Cleft palate (palatoschisis). (**g**) Longitudinal section through columna showing multiple areas of marked spinal cord compression due to abnormal epiphyseal development. (**h**) The centre of the proximal epiphysis of the femur shows non organized hypertrophic chondrocytes and large vessels. Ossification is not present. (HE 75x). Clinical features for BD1 and BD2 are presented in Supplementary Figs 6 and 7. (**i**) Domain and region information for the α1 chain of type II collagen obtained from the UniProt database (http://www.uniprot.org/; accession number: P02459). (**j**) Multispecies alignment of the COL2A1 proteins from different vertebrate species. Note the perfect conservation of the glycine residues of the typical Gly-x-y structural motif, the mutation of which causes hypochondrogenesis/achondrogenesis type II in humans and bulldog calf syndrome in bovine (i.e. p.G600D for BD2, p.G720S for BD3, p.G960R for the cases presented in Daetwyler *et al*.^4^, and p.G996S for BD2). Protein sequences accession numbers in Ensembl are ENSBTAP00000017505, ENSP00000369889, ENSGALP00000035064, ENSACAP00000006225, ENSXETP00000043834 and ENSDARP00000091007.

For example, COL2A1 encodes the alpha-1 chain of type II collagen, a fibrillar collagen found in cartilage and the vitreous humor of the eye in the form of homotrimers. In humans, mutations in this gene can cause as many as 16 different conditions with both recessive and dominant inheritance depending on their nature and location (http://www.omim.org/entry/120140). These conditions include hypochondrogenesis/achondrogenesis type II (MIM: 200610), a dominant syndrome which shows a strong similarity with Bulldog calf syndrome (Fig. 2, Supplementary Figs 6 and 7). Interestingly, hypochondrogenesis/achondrogenesis type II is caused by non-synonymous polymorphisms that (i) disrupt the Gly-x-y structural motif essential for the assembly of the collagen triple-helix^10^ and (ii) occur *de novo* or are inherited from mosaic parents. This is exactly the case for the three BD mutations presented herein and the p.G960R that we previously reported^4^ (Fig. 2, Supplementary Figs 3–5). We thus report four bovine models for human hypochondrogenesis/achondrogenesis type II. Furthermore, in addition to the symptoms commonly described in humans, we observed liver fibrosis at necropsy in some of the affected calves (Supplementary Figs 6 and 7), which supports the view that abnormal accumulation of type II collagen among other extracellular matrix proteins may play a role in organ fibrosis^11^.

The most severe form of *osteogenesis imperfecta* (OI), i.e. OI type II (OMIM 166210) in humans and in the progeny of the Fleckvieh bull “Halvar PP” is characterized by bone fragility, with many pre- or perinatal fractures, severe bowing of long bones, undermineralization, and high perinatal mortality due to respiratory insufficiency (Supplementary Note 1, Supplementary Fig. 8). In humans OI type 2 is caused by heterozygous mutations in the *COL1A1* gene (OMIM 120150) or the *COL1A2* gene (OMIM 120160). Both encode respectively, the pro-alpha1 and pro-alpha2 chains of type I collagen, a fibrillar collagen whose triple helix comprises two alpha1 chains and one alpha2 chain and which is the most abundant protein in the bone matrix^12^. More than 90% (80/88 variants for COL1A1 and 44/46 for COL1A2; http://www.uniprot.org/uniprot/P02452; http://www.uniprot.org/uniprot/P08123 accessed 2016/06/01) of the mutations causing OI type 2 affect the typical Gly-x-y structural motif of these proteins, as is also the case for the COL1A1 p.1049_1050delinsS mutation reported here (Supplementary Fig. 2).

Of note, when genotyping seven affected and 20 unaffected progeny of the mosaic sire using Sanger sequencing, we found an asymptomatic male calf that carried the mutant allele (Supplementary Figs 2 and 9). Inspection of the raw sequencing data indicated an underrepresentation of the mutant allele in this animal, possibly resulting from chimaerism. This phenomenon is not rare in bovines where the twinning rate is about 1% and where anastomoses between twins’ placentas are frequent. Since the male calf was born from a single gestation pregnancy, we hypothesized that (i) it may have received blood cells from a twin that died during gestation or that (ii) it could be the result of an early fusion between two embryos. Genotyping of two different blood samples of this animal using a bovineSNP50 genotyping array yielded evidence that the healthy calf was a chimera and provided a molecular explanation to its unaffected phenotype (Supplementary Fig. 9). Thus, we not only report the identification of the first bovine model for *osteogenesis imperfecta* type 2 in human, but also the exceptional case of an unaffected mosaic bull that sired an asymptomatic chimera. This rare discovery further illustrates the power of the tools and resources available in livestock.

### GEA, an unparalleled animal model for human Tietz syndrome

With the example of GEA, caused by a dominant mutation in *MITF*, we can also demonstrate that the bovine model is, in certain instances, a better model than the mouse.

MITF is a basic helix-loop-helix leucine zipper transcription factor that acts as a master regulator of melanocyte development, function and survival^13^. In humans, mutations in *MITF* cause two distinct autosomal dominant syndromes depending on their type and location. Non-truncating mutations affecting the binding of MITF to the DNA have a dominant negative effect with mutant proteins interfering with the DNA binding domain of wild-type proteins^14^. Such mutations result in an albinoid-like hypopigmentation of the skin, hair and iris and severe hearing loss known as Tietz syndrome (MIM: 103500). In contrast, other MITF mutations cause Waardenburg syndrome type 2A (MIM: 193510) which is characterized by patchy depigmentation and uni- or bilateral deafness.

To our knowledge, seven non-truncating mutations of the MITF basic domain have been reported so far in non-human mammals (Supplementary Fig. 10). MITF mutations p.H209R (mitf^Mi-enu5^ and mitf^Mi–bcc2^), p.I212N (mitf^Mi-wh^), p.R216K (mitf^Mi-Or^) and p.R217del (mitf^Mi^) in mouse, and p.N310S in horse cause a combination of white-spotting, coat color dilution and sometimes hearing loss in the heterozygous state but not Tietz syndrome *sensu stricto* ^15–19^. Because of the mouse-specific tolerance to haploinsufficiency of transcription factor genes^2^, only homozygote animals display a general depigmentation of the skin and hair which, in addition, is associated with other symptoms. In contrast, the two bovine mutations p.R210I^18^ and p.R211del result in a phenotype similar to Tietz syndrome in heterozygotes. Among them, only the MITF p.R211del mutation described in this study is orthologous to a mutation observed in humans, i.e. MITF p.R217del (Supplementary Fig. 10). The latter is the most frequent MITF mutation observed in patients with Tietz syndrome and often occurs *de novo*. Its recurrence might be due to the presence of a short nucleotide triplet repeat^20^. Interestingly, in a recent study, Léger *et al*.^20^ reported that p.R217del does not always result in regular Tietz syndrome but rather in a large range of phenotypes with some patients showing café-au-lait macules. They also reported that sun-exposed freckles were observed more frequently in Asian populations suggesting possible interaction with modifier loci. Finally, Léger *et al*.^20^ highlighted the impossibility in using the mouse as a model for this disease given the differences in transmission in mouse and human. In contrast, the existence of both regular Tietz phenotype and Tietz phenotype with *heterochromia irides* and revertant patches (i.e. small black spots) in MITF p.R211del/+ cattle (Fig. 1; Supplementary Fig. 11) makes GEA a perfect model to investigate the molecular basis of the phenotypic reversion.

**Figure 3:**
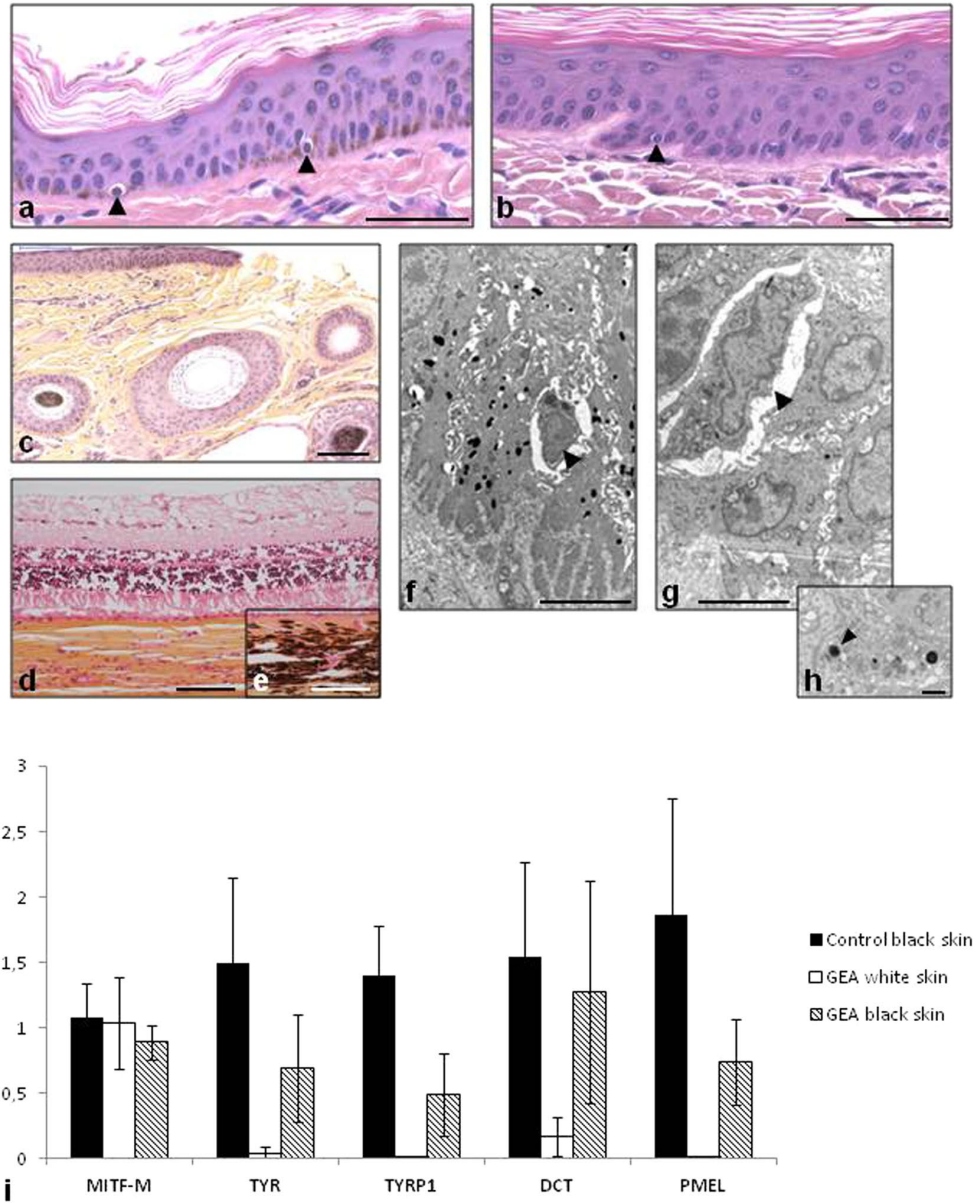
Histological and gene expression analysis of skin biopsies from GEA and control animals. (**a**–**c**) Hematoxylin-eosin-stained sections of ear skin biopsies. (**a**) Black skin from a control black and white Holstein animal. Melanocytes are located on the basal layer of epidermis (indicated by arrowheads) and brown melanin granules are disseminated within keratinocytes. (**b**) Melanocytes are visible, but not melanin in white skin from a GEA animal. (**c**) Black skin from a GEA animal with a revertant phenotype. Melanin is present in the epidermis. Note the presence of both black and white hair. (**d** and **e**) Hematoxylin-eosin-safran stained sections of eyes from GEA and control animals. Note the complete lack of pigmentation of the choroid from the GEA animal (**d**) as compared with control (**e**). (**f**–**h**) Transmission Electronic Microscopy pictures of skin from wild-type (**f**) and from a GEA animal (**g**). Melanocytes (arrowheads) are easily distinguished from the surrounding keratinocytes. Numerous melanosomes lie in the wild-type keratinocytes, while only a few abnormal ones are observed in the mutant skin. (**h**) Detail of a GEA keratinocyte cytoplasm containing abnormal melanosomes (arrowhead). (**i**) Relative expression quantification of melanogenic genes in the skin of wild-type and GEA Holstein animals. *MITF-M* expression is similar in black skin from control animals (n = 3) and in unpigmented (white) and revertant skin (black) from GEA animals (n = 3), showing that the regulation of expression of the melanogenic genes is not a consequence of a downregulation of *MITF-M* in mutant animals. Melanogenic genes (*TYR*, *TYRP1*, and *PMEL*) are not expressed in GEA white skin, with the exception of *DCT* which has already been shown to be regulated by MITF-independent mechanisms. In all black skins sampled from GEA revertant animals, the expression of melanogenic genes is partially restored. Scale bars represent 50 µM in (**a**) and (**b**); 100 µM in (**c**–**e**); 5 µM in (**f**,**g**); and 1 µM in (**h**).

Histological and electron-microscopic studies revealed the presence of melanocytes in white skin samples from MITF p.R211del/+ cattle consisting of round-shaped cells sitting on the basal layer of the epidermis (Fig. 3a–h). Melanin granules and melanosomes were observed in the epidermis of controls, but not in white skin samples from MITF p.R211del/+ animals. Finally, the absence of pigment was evidenced in the choroid of affected *versus* control animals (Fig. 3d,e). This set of evidence confirmed at the microscopic level the perfect similarity between the human and bovine conditions. A gene expression study showed that in white skin samples from MITF p.R211del/+ animals, *MITF* was normally expressed and that, in agreement with the absence of melanin synthesis, MITF target genes such as *TYR*, *TYRP1* and *DCT* were down-regulated (Fig. 3i). Interestingly, the expression of these genes was only partially restored in revertant patches from the same individuals as compared with wild type controls. To gain further insights into the molecular mechanisms involved in this phenotypic reversion, we extracted genomic DNA and RNA from white and black skin biopsies from three MITF p.R211del/+ animals. Sanger sequencing of genomic DNA extracted from white and black skin patches did not reveal any sign of genetic reversion (i.e. of mutation from the mutant allele back to the wild type; Supplementary Fig. 10). In addition, a pyrosequencing test was used to quantify allelic expression. We found no difference in the wild type/mutant allele ratio between white and black skin samples from the same animals (Supplementary Fig. 10) indicating that neither genetic reversion nor allelic imbalance were involved in the phenotypic reversion observed. The persistence of revertant patches throughout life, their location in areas of the body rich in terms of melanocytes, and the dominant negative effect of this mutation^14^ suggest a stochastic aetiology in which rare melanoblasts having slightly higher levels of wildtype homodimers, would undergo a nearly normal differentiation. In conclusion, we present an unparalleled mammalian model for human Tietz syndrome and the first investigation of the molecular mechanism underlying the revertant patches observed in some affected individuals.

### Large half-sib pedigrees allow for the mapping of modifier loci in clinically variable syndromes

The availability of large half-sib pedigrees and routinely collected phenotypes in cattle offers the opportunity to explore aspects of clinically variable syndromes that are difficult to investigate in less prolific species. An exemplary case is CHARGE syndrome in human which, like NC in Montbéliarde cattle, is caused by the haploinsufficiency of CHD7, an ATP-dependent chromatin remodeler essential for activation of core components of neural crest transcriptional circuitry^21^. “CHARGE” is an acronym coined by Pagon *et al*.^22^ referring to five cardinal clinical features of the syndrome: **c**oloboma (of the eyes), **h**eart disease, ***a*** *tresia choanae*, **r**etarded growth and retarded development and/or central nervous system anomalies, **g**enital and/or urinary anomalies, and **e**ar anomalies and/or deafness. Since then, diagnostic criteria have been reevaluated^23–25^; patients should harbor either four major symptoms (ocular coloboma, choanal atresia or stenosis, cranial nerve dysfunction or anomaly, and characteristic ears) or three major and at least three minor symptoms (including genital hypoplasia, developmental delay, cardiovascular malformations, growth deficiency, orofacial cleft, tracheoesophageal fistula, and distinctive facial features) to be considered as having CHARGE syndrome. To date, more than one hundred pathological mutations (including frameshift, splice-site, missense, nonsense and structural variations) have been identified throughout the CHD7 gene. Most of them are private and occurred *de novo*, but a few cases with germline mosaicism and familial inheritance have also been reported^26–31^. No correlation between the type or location of these mutations and phenotypes has been observed even in large cohorts of patients, and different clinical manifestations have been reported in parent/child or sib pairs with the same mutation suggesting the possible involvement of modifier loci^26–31^.

**Figure 4:**
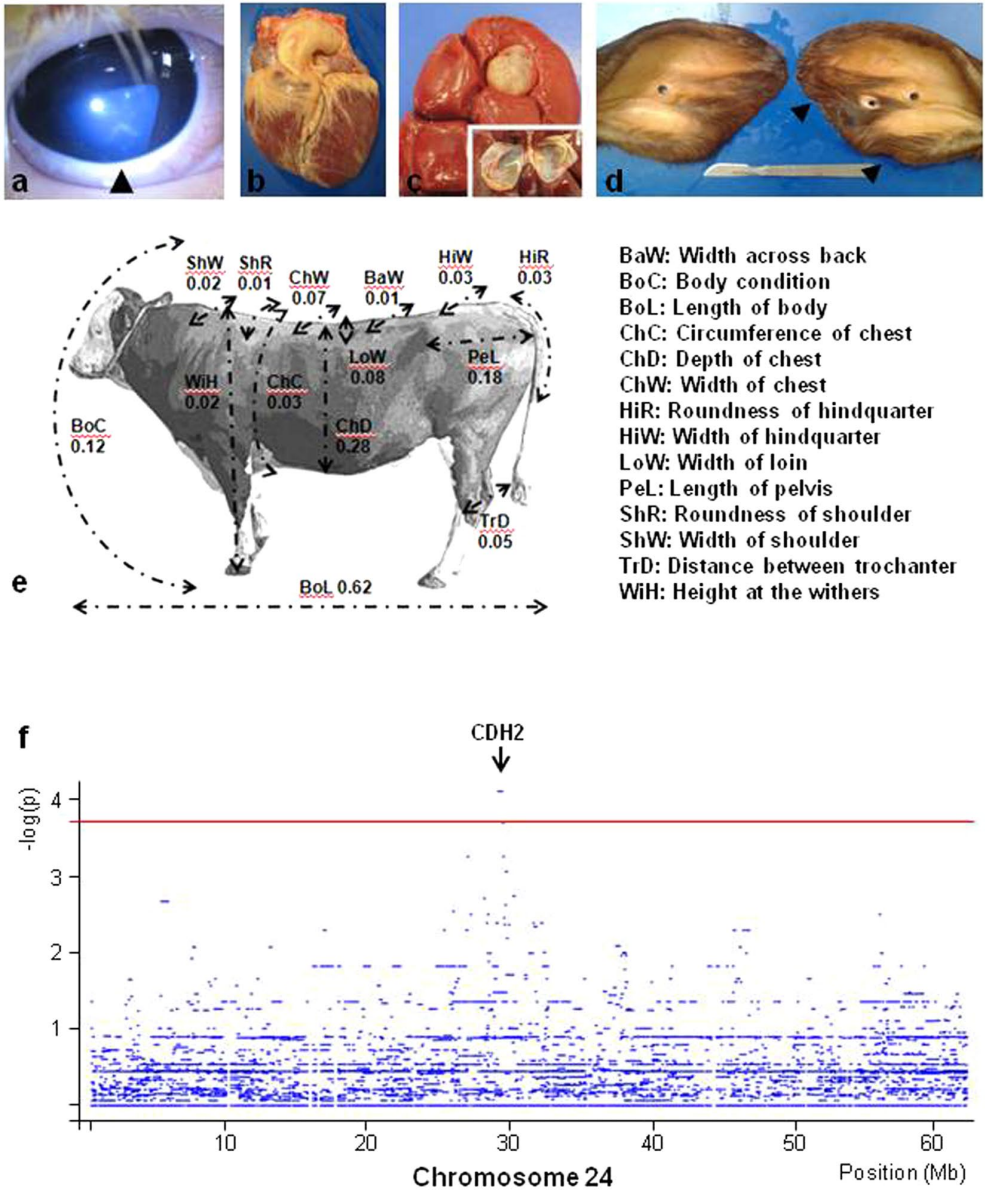
A large bovine half-sib pedigree allows the mapping of modifier loci for CHARGE syndromes. (**a**–**e**) Presentation in CHD7 p.K594AfsX29/+ cattle of the different symptoms which gave rise to the CHARGE acronym in humans (C-coloboma (of the eyes), H-heart disease, A-*atresia choanae*, R-retarded growth and retarded development and/or central nervous system anomalies, G-genital and/or urinary anomalies, and E-ear anomalies and/or deafness). (**a**) iris coloboma highlighted by an arrowhead. (**b**) Heart showing tetralogy of Fallot; note the dextroposition of the aorta. (**c**) Renal cyst and its section. (**d**) Ears from an affected heifer showing abnormalities of the right pinna. *Atresia choanae* is not presented here but was demonstrated by the lack of revulsive reaction of certain animals after smelling alcohol. (**e**) Picture (at four years old) and scores (at 9 months old) of the mutant sire “Etsar” (MONFRAM002528725202) for 14 morphological traits expressed in percentiles with respect to distributions observed in 467 young Montbéliarde bulls raised by the same breeding company with the same protocol. Other symptoms are presented in Supplementary Note 2, Supplementary Figs 12 and 13 and Supplementary Tables 6–7). (**f**) Mapping on chromosome 24 of a locus influencing the clinical features of CHARGE syndrome (see Methods). The red line indicates the chromosome-wide empirical significance threshold of 0.05 as determined by 10 000 permutations of phenotype data (see Methods). Seven consecutive windows of ten markers from BTB-00883964 to ARS-BFGL-NGS-1731 (Chr24:29,132,144–29,762,125) had an empirical p-value of 0.03. Information on the gene content of the corresponding region is presented.

Here the analysis of the numerous descendants (n = 1058) of the NC bull “Etsar” (MONFRAM002528725202), which is heterozygous for a frameshift mutation in CHD7 (p.K594AfsX29), allowed us (i) to observe each of the most common symptoms of CHARGE syndrome and (ii) to improve our knowledge on the variable clinical features which may be associated with this genetic defect (Fig. 4, Supplementary Note 2, Supplementary Figs 12 and 13 and Supplementary Tables 6 and 7). In addition, it gave us a unique opportunity to map modifier loci influencing the clinical features of CHARGE syndrome. Although the usual approach is to compare mildly and severely affected individuals, the rapid death of the latter animals prevented the sampling of a sufficient number of them (n = 7). Therefore, we compared haplotype frequencies for sliding windows of 10 markers between 49 mildly affected animals and 89 unaffected half-sibs genotyped with the Illumina BovineSNP50 Beadchip, using Fisher’s exact test for allelic association. Significant association (chromosome-wide empirical p-value = 0.03) was observed for seven consecutive windows constituting a 16-marker haplotype located on bovine chromosome 24 (marker BTB-00883964 to marker ARS-BFGL-NGS-1731; Chr24:29,068,445-29,893,427; Fig. 4f). The associated segment was present in the heterozygous state in 24.5% (12/49) of the mildly affected animals, 2.2% (2/89) of the unaffected half-sibs, and in none of the seven severely affected animals. This ten-fold enrichment in frequency between the mildly affected and control animals is particularly striking considering that the first group consists approximately of the 10% least affected individuals among carriers of the *CHD7* frameshift mutation (see Methods). It suggests that this haplotype has a major positive effect on the clinical presentation of CHARGE syndrome. Remarkably, the corresponding region contains a single gene, *CDH2*, which is a target of the CHD7 transcription factor according to the CHEA Transcription Factor Targets dataset^32^ available in the Harmonizome^33^ (http://amp.pharm.mssm.edu/Harmonizome). Moreover, conventional and tissue specific inactivation of *CDH2* in mouse have revealed the critical role played by this gene in heart and neural tube formation^34–36^, two developmental processes affected by CHD7 haploinsufficiency. Our results support the involvement of modifier loci in the variable expression of CHARGE syndrome, a hypothesis which has never been confirmed in humans because of the limited number of patients carrying the same mutation.

### Rare conditions occurring in cattle provide interesting models for basic research

We can also demonstrate that natural mutations occurring in livestock species, such as the p.R160C substitution in COPA, can provide interesting models for basic research.

COPA is a subunit of the coat protein I complex (COPI), which mediates the retrograde transport of dilysine-tagged proteins from the cis-Golgi to the rough endoplasmic reticulum (ER)^37, 38^. Several mutations affecting other subunits of COPI have been reported to cause hypopigmentation together with severe phenotypes in zebrafish^39^ and mouse^40^, suggesting a possible involvement of this protein complex in pigmentation.

In an independent study, Dorshorst *et al*.^41^ identified the same candidate mutation for DR phenotype in Holstein cattle that we presented at the World Congress on Genetics Applied to Livestock Production held in Vancouver^42^. The approach described in our study proved definitively the *de novo* nature and the causality of the mutation. Hair pigment analysis^41^, expression studies, and histological analyses revealed that the COPA p.R160C substitution causes a downregulation of melanogenic genes and a switch towards the production of pheomelanin in animals which, being homozygous for a constitutively active MC1R receptor, should produce only eumelanin (Supplementary Figs 14 and 15).

**Figure 5:**
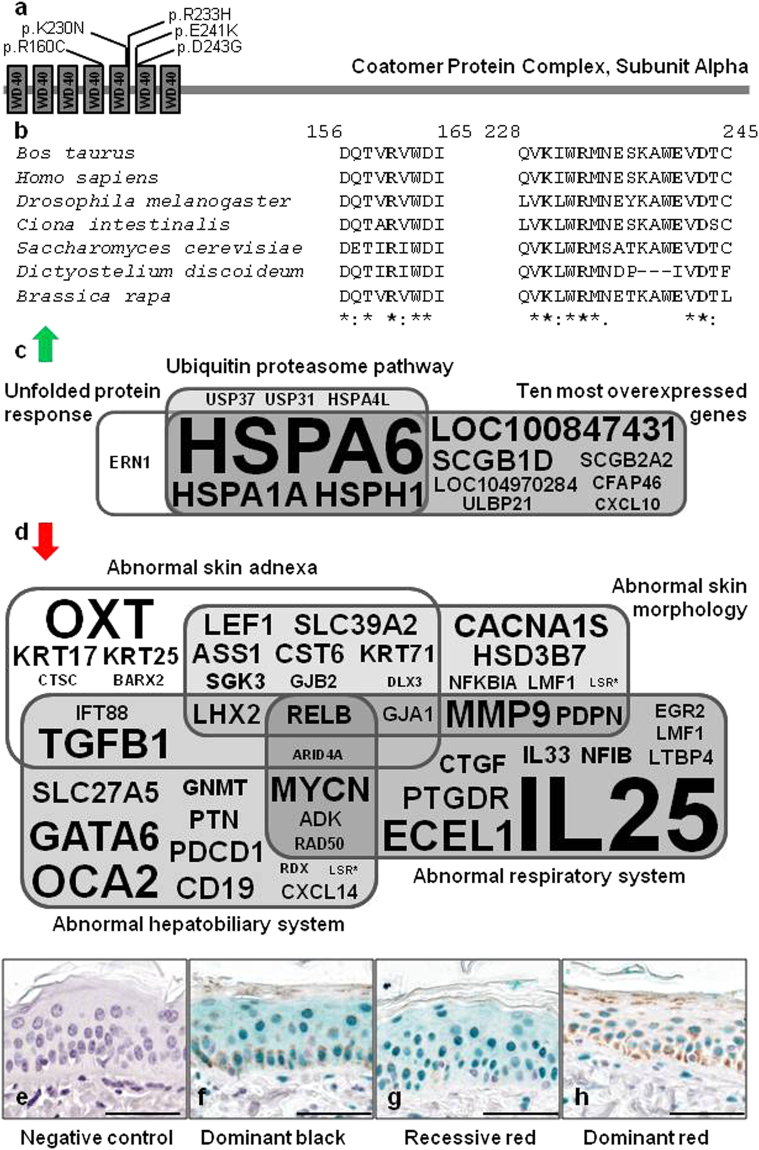
Characterization of the effect of bovine p.R160C substitution in COPA. (**a**) Domain information for COPA obtained from the UniProt database (http://www.uniprot.org/; accession numbers: Q27954 and P53621) and localization of the natural mutations described in cattle (p.R160C) and in human (p.K230N, p.R233H, p.E241K, p.D243G). The coordinates are identical in human and bovine orthologs due to the strong conservation of the WD40 domain that interacts with dilysine motifs^44^. (**b**) Multispecies alignment of COPA proteins illustrating the strong conservation of the mutated residues among eukaryotes. Protein sequences accession numbers in Ensembl are respectively ENSBTAP0000000567, ENSP00000357048, FBpp0072694, ENSCINP00000018763, YDL145C, EAL73444 and Bra010674.1-P. (**c**) Ingenuity Pathway Analysis on genes that are markedly upregulated in the skin of dominant red versus dominant black Holstein cattle (fold change ≥2; FDR < 0.05; according to the RNAseq data produced by Dorshorst *et al*.^41^; Supplementary Data 1) identifies the unfolded protein response and ubiquitin proteasome pathway as the two most significantly enriched canonical pathways (P < 0.001). (**d**) Functional annotation of downregulated genes in the same RNAseq data using Enrichr^46, 47^ reveals a significant enrichment (P < 0.05) for four “MGI Mammalian Phenotype Level 3′. Results of gene set enrichment analyses are presented in Supplementary Tables 8 and 9. Font size is proportional to the fold change in (**c**) and to the inverse of the fold change in (**d**). (**e**–**h**) Immunohistochemical analysis with rabbit polyclonal antibodies against NFI transcription factors in epidermis from dominant black (MC1R^D/D^, DR^+/+^), recessive red (MC1R^e/e^, DR^+/+^), and dominant red (MC1R^D/D^, DR^DR/+^) Holstein animals. Scale bars correspond to 50 µm. NFI proteins are revealed with HRP-Green system, and all samples have been counterstained with hematoxylin. Staining is absent in negative control (**a**; recessive red), shows a nuclear and cytoplasmic distribution in dominant black (**f**) and recessive red (**g**) skins, while the distribution is only nuclear in dominant red skin (**h**). This observation was confirmed in samples from different animals (n = 3, 2 and 3 for **f**–**h** respectively).

Little is known about the clinical consequences of COPA mutations in vertebrates. To our knowledge, only four mutations have been described so far in humans^43^ (Fig. 5a). All these mutations are located in a particular region of the human protein, at the end of the fifth - and at the beginning of the sixth WD40 repeat, and cause a new dominant syndrome with incomplete penetrance characterized by autoimmune-mediated lung disease and arthritis^43, 44^ (i.e. “COPA syndrome”). These human mutations have no visible effect on hair pigmentation (Anthony Shum, pers.comm.), which is in contrast with the bovine p.R160C mutation, located in the fourth WD40 repeat of COPA. Despite differences in clinical manifestations and localization within the WD40 repeat domain of the protein, we assumed that the bovine and human mutations might share common pathogenic mechanisms, possibly associated with abnormal retrograde Golgi-to-ER transport of dilysine-tagged proteins. To test this hypothesis we reanalyzed the RNA-sequencing data produced by Dorshorst *et al*.^41^ (Supplementary Data 1).

Strikingly, Ingenuity Pathway Analysis identified the unfolded protein response (UPR) and ubiquitin proteasome pathway (UPP) as the two most significantly enriched canonical pathways (P < 0.001) among genes that are markedly upregulated in the skin of DR versus black Holstein cattle (fold change ≥2; FDR < 0.05; Fig. 5b and Supplementary Table 8). The UPR is a cellular stress response activated in reaction to an accumulation of un- or misfolded proteins in the ER. Notably, studies in humans have demonstrated that COPA variants impair binding to proteins targeted for retrograde Golgi-to-ER transport and cause a marked increase in ER stress in the lung epithelium and alveolar macrophages. The UPP is the principal mechanism for protein catabolism in the cytosol and nucleus of eukaryotic cells^45^. The upregulation of this pathway, in the context of upregulation of the UPR, is consistent with the need to degrade increased amounts of abnormal proteins that have been removed from the ER to preserve its homeostasis.

In addition, we annotated the function of the genes that are downregulated in the skin of DR versus black Holstein cattle (fold change ≤1; FDR < 0.05) using Enrichr^46, 47^. We observed a significant enrichment (P < 0.05) for four “MGI Mammalian Phenotype Level 3”: abnormal skin adnexa, abnormal skin morphology, abnormal hepatobiliary system, and abnormal respiratory system (Fig. 5c, Supplementary Table 9). The latter enrichment is particularly interesting considering that COPA mutations cause chronic pulmonary disease in humans. It is even more striking to note that it comprises genes whose mutation in mice cause lung malformations (e.g. *CTGF* ^48^, *NFIB* ^49^ and *PDPN* ^50^) and genes involved in immune response/inflammation (e.g. *IL25* ^51^, *IL33* ^52^, *MMP9* ^53^ and *RELB* ^54^). Such a combination of general perturbation of the immune system and subnormal development of a specific organ could explain the tissue-specific nature of the autoimmune manifestations observed in human COPA syndrome.

Three genes among those that are downregulated in skin samples from DR versus black Holstein cattle particularly caught our attention (*RELB*, *IL25* and *NFIB*). *RELB*, is the only gene common to the four Mammalian Phenotypes previously mentioned. Its loss-of-function in mouse causes inflammatory cell infiltration of organs^54^, a frequent finding in patients with autoimmune disorders. Moreover, Valéro *et al*.^55^ demonstrated that defective NF-kappa B/RELB pathway was associated with inefficient expansion of thymocyte and dendritic cells in autoimmune-prone New Zealand black mice. IL25 is the second most downregulated gene (fold change = 0.11) and a key regulator of inflammatory and autoimmune diseases^51^. Downregulation of both *RELB* and *IL25* genes could play a central role in autoimmunity in COPA syndrome patients. Finally, to the best of our knowledge, *NFIB* is the only gene among 642 differentially expressed genes (FDR < 0.05; Supplementary Data 1) to potentially affect both lung development and melanogenesis. Indeed, NFIB is essential for lung maturation^49^ and also controls epithelial-melanocyte stem cell behaviour^56^. A downregulation of *NFIB* (fold change = 0.62; P < 0.0001) could be the primary cause of the downregulation of eight hair keratin genes (*KRT17*, *KRT25*, *KRT35*, *KRT71*, *KRT73*, *KRT74*, *KRT79* and *KRT85*; 0.51≤ fold change ≤0.66) and the differential expression of five genes involved in melanogenesis (*ATRN* which is upregulated with a fold change of 1.74 and *LEF1*, *OCA2*, *TFAP2A*, and *TYRP1* which are downregulated; 0,36≤ fold change ≤0,73). Interestingly, immunohistochemical analysis with rabbit polyclonal antibodies against NFI transcription factors (i.e. NFIA, NFIB, NFIC and NFIX) revealed marked differences in terms of intracellular signal distribution between skin samples from DR animals (COPA+/−) and dominant black and recessive red animals (COPA+/+). This result suggests that, in skin epithelial cells, COPA p.R160C mutation affects not only the expression of NFIB but also the cellular transport of at least one of the NFI transcription factors (Fig. 5e–h).

In conclusion, these results call for a thorough clinical examination of COPA+/− cattle and provide interesting avenues to explore in order to better understand the pathogenetic mechanisms associated with mutations affecting WD40 repeats of COPA.

### Detection of *de novo* mutations in the genome sequence of elite sires – towards in silico identification of recessive genetic defects

After identifying mutations causing dominant conditions, we investigated the feasibility of anticipating the emergence of recessive genetic defects by detecting *de novo* deleterious mutations in the genome of healthy AI bulls. To achieve that goal, we screened the genomes of 43 French sires (24 Holstein, 11 Montbéliarde, five Normande and three Charolais) born one to four generations before the current population (since recessive defects typically emerge at the fifth generation with the first consanguineous mating between descendants of a common ancestor).

We identified 18 private deleterious mutations, out of which seven were confirmed to be *de novo* mutations after genotyping by PCR and Sanger sequencing of the bulls and several of their close relatives (Methods, Supplementary Table 10, Supplementary Fig. 16). Except for *FAM189A1* and *CSNK1G2* for which, to our knowledge, no homozygous mutant has been described in mammals, the five other mutations in *COL6A3*, *EDAR*, *ITGA3, SLC35A3* and *SOWAHB*, are expected to cause severe recessive conditions leading to embryonic lethality, perinatal mortality or euthanasia according to the literature (Supplementary Table 10).

**Figure 6:**
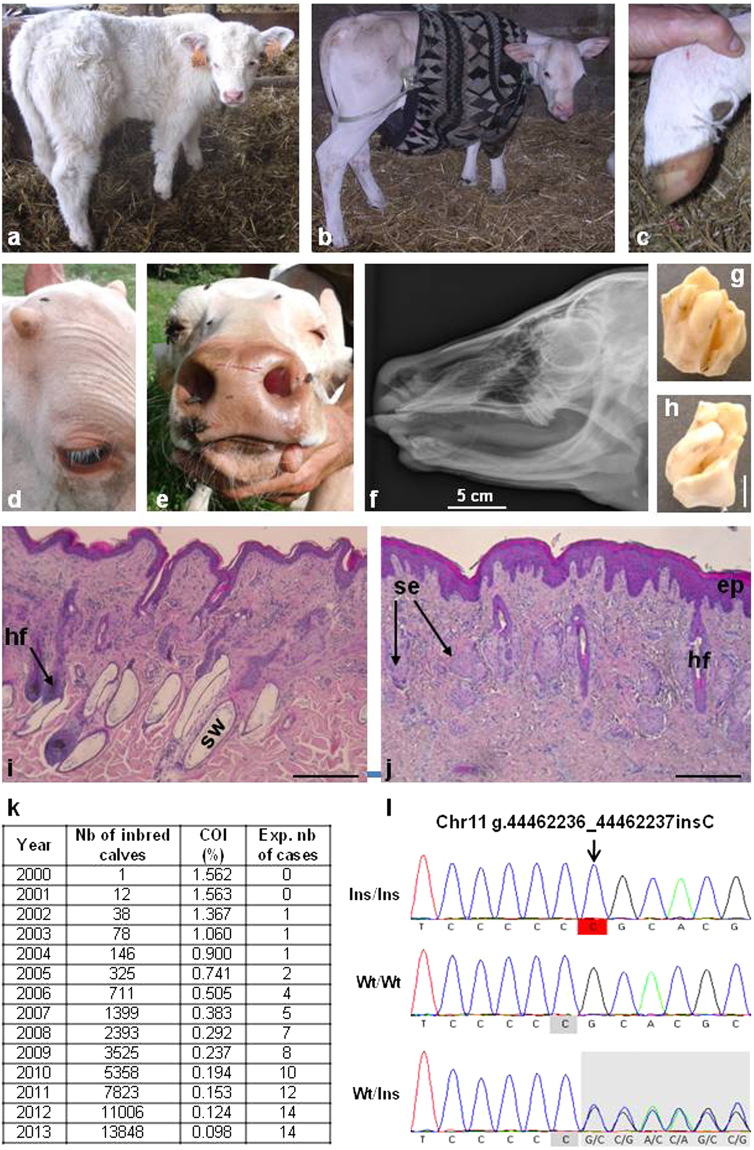
Identification of a *de novo* mutation causing recessive anhidrotic ectodermal dysplasia (AED) before the emergence of this defect in the population. (**a**) One-month old control calf. (**b**) One-month old AED affected calf showing generalized hypotrichosis. (**c**) Hoof of the same AED calf with no notable malformations. Note that in humans most AED patients have normal nails too. (**d**) Detail of the head of a five-month-old AED calf, showing eyelashes and normal development of horns. (**e**) Muzzle of the same calf showing normal vibrissae and extreme skin dryness due to the absence of nasolabial glands (see also Supplementary Fig. 19). (**f**) X-ray of the skull of a one-month old calf showing a complete absence of incisors and the presence of only one upper molar on each side. (**g**,**h**) Detail of one upper molar showing abnormal cusps and deep grooves. (**i**,**j**) Histological sections of skin biopsies from control (**i**) and AED (**j**) one-month-old calves. In AED animals, the epidermis (ep) is acanthotic (i.e. thickened). The number of hair follicles is greatly reduced and deep portions of them are atrophic or absent. Sweat glands (sw) are absent. Sebaceous glands (se) appear normal but a number of them are “orphan” (i.e. without associated hair follicle). Scale bars represent 500 µm. (**k**) Yearly estimation of the number of calves predicted to be born homozygous for the g.44462236_44462237insC mutation on chromosome 11 which is predicted to cause a frameshift and premature truncation of EDAR. The total numbers of calves with both parents related to primo-mutant bull Invincible, as well as their coefficient of inbreeding (COI) are also presented. (**l**) Electrophoregrams of a homozygous (Ins/Ins) AED affected calf, a homozygous wild-type (Wt/Wt) animal and a heterozygous carrier of the mutation (Wt/Ins).

As a proof of concept, we investigated the putative effect of a frameshift mutation in the Ectodysplasin A Receptor (EDAR p.P161RfsX97) that was found on the paternal chromosome of the Charolais bull “Invincible” (CHAFRAM001893105503 born in 1993).This mutation is predicted to result in a truncated receptor lacking the Death Domain (Supplementary Fig. 17) which is essential for the interaction with the EDAR-Associated Death Domain Protein (EDARADD) and the activation of downstream signaling pathways^57^. In humans, mutations in EDAR and in other members of the ectodysplasin pathway (i.e. EDA and EDARADD) are known to cause anhidrotic ectodermal dysplasia^58^ (AED, MIM: 305100, 129490, 224900, 614940 and 614941), a syndrome characterized by hypotrichosis (sparseness of hair), hypo- or an-hidrosis (reduced ability to sweat), and hypodontia (congenital absence of teeth). Here, we took advantage of the Charolais natural mating population which has a shorter generation interval than AI breeding schemes and mined the pedigree databases for consanguineous matings between descendants of “Invincible”. We estimated that 14 out of 344,564 Charolais calves born in 2013 were expected to be homozygous mutants. In agreement with this estimation, a survey enabled us to detect ten calves, born from unaffected parents, and showing symptoms of anhidrotic ectodermal dysplasia (see Methods, Fig. 6 and Supplementary Figs 18 and 19). Of note, affected calves grew normal horns suggesting that the ectodysplasin pathway, which plays a key role in the differentiation of skin appendages in vertebrates^58^, is not involved in horn ontogenesis. Thus, these animals represent an appealing model in evolutionary and developmental biology. Genotyping of seven affected calves using the Illumina BovineSNP50 array revealed that, at the genome scale, they were homozygous for a single shared haplotype of 26 Mb on chromosome 11, encompassing *EDAR*. Subsequent genotyping by PCR and Sanger sequencing confirmed that these seven animals were also homozygous for the EDAR p.P161RfsX97 mutation. Furthermore, screening of the French genomic selection database (8179 Charolais animals) revealed the existence of three unaffected animals which were also homozygous for the same 26-Mb IBD segment but only heterozygous for the *EDAR* mutation. Pedigree analysis demonstrated that one of the haplotypes of these individuals could be traced back to Invincible whereas the other haplotype originated from the sire of Invincible and not directly from him. These results confirm the causality of the mutation and validate our approach for anticipating the emergence of new recessive disorders.

Importantly, this strategy can easily be adapted to investigate the phenotypic consequences of *de novo* mutations affecting poorly or uncharacterized genes such as *FAM189A1* and *CSNK1G2* and thus improve the functional annotation of mammalian genomes.

## Conclusion

We have demonstrated that the availability of large databases in cattle combined with the typical structure of livestock populations facilitate the rapid detection and functional characterization of *de novo* deleterious mutations. The study of mutations underlying sporadic syndromes in cattle, which also occur in humans, offers an interesting alternative to laboratory animals for confirming the genetic aetiology of isolated clinical case reports and gaining insights into the molecular mechanisms involved. In addition, the study of mutations affecting poorly characterized genes increases the attractiveness of cattle and other farm animals to annotate their functions and become models for basic research. Finally, with the increasing sequencing of young elite bulls, the detection of *de novo* deleterious mutations in their genomes will enable the management of recessive defects in livestock populations before a large number of affected calves emerge.

## Methods

### Ethics statement

Blood and ear biopsies were collected by veterinarians or by agricultural technicians licensed by the French Departmental Breeding Establishments (Etablissements Départementaux de l’Elevage (EDE)) during routine ear tagging, sampling for annual prophylaxis, paternity testing and genotyping for genetic defects or genomic selection. Semen was provided by breeding organizations. All invasive procedures were performed *post-mortem* either on animals that had died of natural causes, or after euthanasia or slaughter for meat production, thus no ethical evaluation was required. All animals used in this study were kept according to Swiss or European laws for bovine production, and experiments reported in this work comply with the ethical guidelines of the French National Institute for Agricultural Research (INRA) and its research partners participating in the present study. All the samples and data analyzed were obtained with the permission of breeders, breeding organizations and contributing research groups.

### Animals, sampling and phenotyping (all the studies)

Details on animals, phenotypes and sample collection for each condition and each analysis are presented in Supplementary Note 1 and Supplementary Table 1.

### DNA extraction (all the studies)

Genomic DNA was extracted from blood, tissues or sperm using standard extraction protocols. For quantifying allelic dosage in white and black skin samples from revertant GEA animals using pyrosequencing, genomic DNA was extracted from cryocuts from ear biopsies (without hair or cartilage) with the Nucleospin tissue genomic DNA kit (Macherey-Nagel).

### Whole Genome Sequencing, read mapping, variant calling and filtering for heterozygous private deleterious mutations (all the studies)

Paired-end libraries with a 250-bp (for BD1, DR, GEA and NC) or a 400-bp (for BD2, OI, and the BD3 trio) insert size were generated using the TruSeq DNA Sample Prep Kit (Illumina; for DR and GEA) or the NEXTflex PCR-Free DNA Sequencing Kit (Biooscientific; for BD1, BD2, NC, OI, and the BD3 trio). Libraries were sequenced on one lane of Illumina HiSeq. 2000, 2500 or 3000 instruments each using 2 × 101 bp (for BD1, DR, GEA and NC) or 2 × 150 bp paired-end reads (for BD2, OI, and the BD3 trio). Reads were mapped on the UMD3.1 bovine sequence assembly using BWA-MEM^59^. Reads with multiple alignments were removed. PCR duplicates were filtered and variants were called using SAMtools rmdup and pileup options^5^. Only variants with a quality score (QUAL) of > = 30 and a mapping quality (MQ) score of > = 30 were considered with the exception of BD2 for which a less stringent quality threshold (QUAL > = 15) was applied due to the lower genome coverage (9 x) as compared with the other defects. For each genetic defect we assumed the causative mutation is dominant and occurred *de novo*. We therefore retained only heterozygous polymorphisms that were absent from analogous whole genome sequencing data from 1230 control animals (Supplementary Table 1). Analysis of pedigree information, demonstrated that none of the 1230 control animals were descended from any of the first mutant animals for the different conditions studied. Within the genome of each affected animal, heterozygous private variants supported by only one read on each strand were removed to avoid possible artefacts created by reading twice an error present on a fragment displaying an insert size that was too small. SNP and Indels were then annotated using Ensembl VEP^6^. Only private frameshift mutations, in-frame insertions or deletions, stop gain or loss variants, polymorphisms affecting splice donor or splice acceptor sites, and deleterious missense polymorphisms were retained. The reliability of the annotation was verified on the UCSC genome browser (http://genome.ucsc.edu/; accessed 2016/05/10) using the “RefSeq Genes”, “Non-cow RefSeq genes”, “Cow mRNAs from GenBank” and “Cow ESTs that have been spliced” tracks. Polymorphisms predicted to affect a transcript that was not supported by the alignment of bovine mRNAs or orthologous genes were considered as non-coding variants. Finally, to predict the phenotypic consequences of the remaining variants, we made a search for the corresponding genes in the Online Mendelian Inheritance in Man (OMIM; www.omim.org) and Mouse Genome Informatics (MGI; www.informatics.jax.org) databases.

The same approach was used to detect heterozygous private deleterious mutations in the genomes of healthy AI sires to anticipate the emergence of novel recessive disorders. For this pilot study we selected 43 bulls used in France (to benefit from the network of the French National Observatory of bovine Anomalies for subsequent verifications; http://www.onab.fr/) and born one to four generations before the animals currently in use (since consanguineous mating between descendants of a common ancestor are more likely to occur after the fifth generation). These 43 animals consisted of 24 Holstein, 11 Montbéliarde and five Normande bulls born in 1996 or after, and three Charolais bulls born in 1993 or after. Two generations of 6.5 or 7 years and two subsequent generations of 2.5 or 3.5 years were considered for dairy and beef breeds, respectively, to account for the reduction of generation interval recently allowed by genomic selection. In a first step, we selected the heterozygous deleterious variants that were found only in one of the 43 genomes and not in the other control genomes (Supplementary Table 1). These private deleterious variants consists either (i) in recent mutations that occurred on haplotypes that are also observed in their original form (i.e. without these mutations) in other genomes in the dataset or (ii) in rare (and predominantly ancient) polymorphisms located on haplotypes carried by a single animal in the dataset. To discriminate these two groups of variants, and to reduce as much as possible the proportion of false positive results in our screening for deleterious *de novo* mutations, we calculated the number of private mutations in a +/− 2.5 Mb interval around each private deleterious mutation in the genome of each carrier animal. We considered arbitrarily that private deleterious variants observed in animals having 10 private mutations or less in the investigated intervals were likely recent mutations that occurred on non-rare haplotypes. Therefore, we retained only these mutations for further studies.

Because the lack of large databases for structural variations precluded their efficient filtering, we did not systematically investigate these polymorphisms (which are expected to represent only 1% of the total genomic variation). However, we used Pindel^7^, DELLY^8^ and IGV^9^ to identify structural variants in the BD3 trio and in the GEA, DR and NC mapping intervals (see below).

### Mapping of the DR, GEA and NC defects

Animals were specifically sampled according to their phenotype or origin, or were extracted from the large French national data base of genotypes for genomic selection. Animals were genotyped with the Illumina BovineSNP50 or EuroG10K Beadchips (Details in Supplementary Table 1). The EuroG10K Beadchip is a custom version of the BovineLD v2.0 BeadChip (https://support.illumina.com/downloads/bovineld-v2-0-product-files.html) comprising the same core marker set of 7931 SNP and fluctuating sets of additional markers. All of these genotypes were included in the genomic selection procedure^60^ and were phased and imputed with the FImpute software^61^ considering large bovine reference populations (231,115 Holstein and 99,044 Montbéliarde animals). For DR, the whole French Holstein data base was investigated to find descendants of the founder female Surinam Sheik Rosabel-RED (HOLCAN000003541221). Animals homozygous for the recessive MC1R^e^ red allele were excluded. Genotypes at the MC1R locus are predicted in the routine genomic evaluation according to haplotype-based information (Capitan *et al*., unpublished; Supplementary Table 12). Finally, phenotypic information could be retrieved from the French breeding companies for 31 DR and 36 black descendants of Rosabel.

For each defect, 43801 autosomal markers which passed quality control for the current French genomic selection procedure were considered. Phenotypic segregations in the different pedigrees were analyzed and, for each case, only the (maternal or paternal) haplotype assumed to carry the causative mutation was conserved. Similarly, for control, only the haplotype assumed to carry the wild type allele was retained. The causative mutation was mapped by searching a haplotype shared by all the cases and absent or at a low frequency in the control group. Haplotypes were formed by considering a window of 10 consecutive markers (~500 kb) sliding over the whole genome.

### Screening for positional candidate variants in the DR, GEA and NC mapping intervals

All the private heterozygous variants found in affected animals within each of the associated intervals were considered. In addition, since for technical reasons structural variants have not been investigated in the complete dataset of whole genome sequences, we performed detection of this type of polymorphisms in the DR, GEA and NC mapping intervals to ensure that we did not miss any candidate mutation. We analyzed the genomes of one case and 20 control animals per interval using Pindel^7^, DELLY^8^, and visual examination of the whole-genome sequences with IGV^9^ Control animals were selected based on their genotypes for the SNP and small indels previously detected, according to the number of rare alleles shared with the case. The aim of this selection was to represent both haplotypes from affected animals within the mapping interval.

### Verification of the *de novo* nature of the heterozygous private variants (all the studies)

For BD3, the *de novo* nature of the candidate mutations was confirmed using whole genome sequencing data of the parents of the affected animal, and PCR and Sanger sequencing. Details on the animals and PCR primers used are presented in Supplementary Tables 1 and 11 respectively. PCR reactions were performed using the Go-Taq Flexi (Promega) according to the manufacturer’s instructions on a Mastercycler pro thermocycler (Eppendorf). The resulting amplicons were purified and bidirectionally sequenced by Eurofins MWG (Germany) using conventional Sanger sequencing. Polymorphisms were detected with the NovoSNP software^62^.

For BD1, BD2 and OI, the *de novo* nature of the candidate mutations was confirmed using PCR and Sanger sequencing in a panel of affected and unaffected animals, and DNA samples extracted from the semen (for BD2 and OI) and/or the blood (for BD1, BD2 and OI) of the mosaic sires.For all other candidate *de novo* variants, a panel of animals including cases and related controls with both DNA and Illumina BovineSNP50 Beadchip haplotype data available in the French Genomic Selection DNA bank and database was genotyped by PCR and Sanger sequencing. In a first step, the segregation of the putative *de novo* alleles and haplotypes of 100 markers (5 Mb) surrounding these mutations were studied among the descendants of putative first mutant animals. Haplotypes carrying these mutations were identified and, in a second step, ancestors of the putative primo-mutants were screened. Identification of carriers of the same identical-by-descent haplotypes but not of the mutant alleles was interpreted as a confirmation of the *de novo* nature of the mutation. For DR, since no ancestor of the first mutant heifer was available in our data set, we mined the French Genomic Selection database to identify AI bulls which (i) shared the same haplotype as DR animals over the whole DR interval but which (ii) were phenotypically black. Among them, JOCKO BESNE (HOLFRAM005694028588), which presented the longest haplotype (interval Chr3:7,928,589–30,728,145) in common with a DR animal (HOLCHEM120093681213), was selected as a control carrying the same IBD haplotype but without the DR mutation.

### Genotyping of the AED pedigree

Seven affected calves, as well as ten dams and four sires of affected calves were genotyped with the Illumina BovineSNP50 Beadchip. Haplotype phases were inferred as described above, using a reference set of 8179 Charolais animals. A homozygosity mapping approach^63, 64^ was applied to verify that the seven cases were homozygous for a unique shared IBD segment in their genomes encompassing the *EDAR* frameshift mutation. In addition, the Charolais database was screened for identifying healthy animals which (i) were also homozygous for the same 26-Mb IBD segment and (ii) descended from Invincible on only one side of their pedigree and from its sire on the other side (Invincible’s sire carries the same haplotype but without the *EDAR* frameshift mutation). DNA from three of these animals, as well as the affected calves and their parents were finally genotyped for the frameshift mutation by PCR and Sanger sequencing as previously described.

### Mapping of modifier loci for bovine CHARGE syndrome (NC study)

Because of the rapid death of the most severely affected animals, we were able to obtain DNA samples from only 7 of them. This low number is not high enough for mapping but can be used for confirmation. To map modifier loci, we used 89 non-carrier animals and 49 mildly affected animals that were still alive at the age of 6 months. All animals were descendants of the NC sire and of a homogeneous purebred Montbéliarde dam population. Considering that half of the 1058 progeny of this bull carried the mutation and that we phenotyped and genotyped most of the progeny still alive at the time of the study, these 49 mildly affected animals correspond approximately to the 10% least affected among carriers. The mapping method relied on a comparison of haplotype frequencies between both groups (mildly affected *vs* non affected) for sliding windows of 10 consecutive markers over the whole genome. For chromosome 14, which contains the CHD7 frameshift mutation, only maternal haplotypes were considered. A Fisher’s exact test was used and 10 000 permutations were computed to determine genome-wide and chromosome-wide empirical P values.

### Analysis of at risk matings and estimation of the number of AED affected calves per year

The expected number of affected animals was determined by analyzing the complete French Charolais pedigree (27 million animals) using the Pedig software^65^. In a first step, all descendants of the bull “Invincible” were identified and his contribution to their gene pools calculated. Then, calves born from consanguineous matings were identified and their risk of being homozygous for the *EDAR* frameshift mutation (which occurred *de novo* in Invincible) was computed as c_sire_ * c_dam_/4 (with c_sire_ and c_dam_ being the contribution of Invincible to the gene pool of the sire and the dam of the inbred calves). Finally, the number of affected cases per year was estimated by summing the risk probabilities for all the inbred calves born during the same period.

### Histological analyses (AED, BD3, DR and GEA studies)

For optic microscopy analyses, skin biopsy, bone section and eye specimens were fixed in 10% neutral buffered formalin for 24 hours and embedded in paraffin wax. Sections (4 µm) were stained with haematoxylin and eosin (HE) or with haematoxylin, eosin and safran (HES). Hairs samples from two bulls, one dominant red (MC1R^D/D^ & DR^DR/+^) and one recessive red (MC1R^e/e^ & DR^+/+^), were placed in a drop of lactophenol on a microscope slide and covered with a cover slip before examination. The number and shape (with or without tails) of eumelanin patches was evaluated at magnification × 400 on a total of ten hairs per animal sampled on the flank (n = 5) and on the back (n = 5). Photographs were taken with a Nikon camera device.

For immunohistochemical analysis, formalin-fixed, paraffin-embedded skin sections (4 µm) of dominant black, (MC1R^D/D^ & DR^+/+^), recessive red and dominant red animals were mounted on the same slide and deparaffinised. Antigen retrieval was done for 1 hour in 10 mM sodium citrate buffer pH6 using a decloaking chamber. Nonspecific binding sites were blocked by incubation with 2.5% horse serum (ImmPRESS kit, Vector Laboratories). The sections were incubated with 4 µg/mL of primary antibody overnight at 4 °C (polyclonal rabbit anti-NFI (H300), reference sc-5567 Santa Cruz Biotechnology). After rinsing with 1 mM PBS (pH 7.5) + 0.05% Tween20, the slides were incubated with the secondary antibody (HRP anti-rabbit IgG, ImmPRESS kit, Vector Laboratories), for 30 min at room temperature. The signal was revealed using HRP-Green kit (Zytomed systems), and the slides were counter stained with hematoxylin.

For electron microscopy analyses, samples were fixed with 2% glutaraldehyde, in 0.1 M sodium cacodylate buffer (pH 7.2) for 4 hours at room temperature. Then, they were postfixed with 1% osmium tetroxide containing 1.5% potassium cyanoferrate, gradually dehydrated in ethanol (30–100%) and embedded in Epon. Semi-thin sections (500 µm) and thin sections (70 nm) were cut with a microtome. Thin sections were collected onto 200 mesh cooper-Paladium grids. Sections were stained with lead citrate. Specimens were examined with a Hitachi HT7700 electron microscope operated at 80 kV. Microphotographs were acquired with an AMT charge-coupled device camera.

### Genomic DNA, total RNA extraction and cDNA preparation from skin biopsies (DR and GEA studies)

Tissues samples were stored at −80 °C and cryocuts without hair or cartilage were used to extract genomic DNA and total RNA from ear skin biopsies. Genomic DNA was purified using the “Genomic DNA from tissue Nucleospin kit” (Macherey Nagel), after homogenising 10 mg of cryocuts in lysis buffer. RNA extraction was performed with the RNeasy mini kit (Qiagen) for GEA sample series or with the NucleoSpin kit (Macherey-Nagel) for the Dominant Red series, and quality control checked on a Bionanalyzer. Only samples with a RIN > 6.8 were considered. Considering the one-exon structure of the MC1R gene, a DNAse treatment with the DNA-free^TM^ DNA removal kit (Life Technologies) was performed on the samples from the VR series. cDNA samples were prepared using the Superscript III First Strand cDNA Synthesis System (Life Technologies), with 100 ng to 1 µg total RNA as starting material, and an equimolar mixture of oligodT and random hexamers primers. Finally cDNA concentration was measured using a NanoDrop ND-1000 spectrophotometer (Thermo Fischer Scientific).

### Analysis of gene expression by qRT-PCR (DR and GEA studies)

In a final volume of 20 µL, 5 ng of cDNA were mixed with 10 µL of SYBR Green® mastermix (Applied Biosystems) and 300 nM of forward and reverse primers. The reaction was programmed on a QuantStudio^TM^ 12 K Flex Real-Time PCR System (Life Technologies) as follows: 50 °C for 2 min, 95 °C 5 min, followed by 40 cycles (95 °C for 15 sec and 60 °C for 1 min). The primer sequences are indicated in Supplementary Table 11. To ensure a reliable quantification of transcripts in bovine skin, the stability of three genes (*RPL32*, *GAPDH* and *β2*-*microglobulin*) was tested in a set of 11 samples (from two dominant black, two recessive red and seven dominant red Holstein animals). Their expression stability was calculated using the ExpressionSuite software (Life Technologies), and RPL32 was considered as the less variable gene (score 0.474) compared to GAPDH (score 0.497) and *β2*-*microglobulin* (score 0.643). The target transcripts were further quantified using RPL32 as a reference gene. The relative quantification of each transcript was calculated with the ΔΔCt method of the ExpressionSuite Software (Life Technologies). Transcript expression levels of transcripts in one black Holstein individual were set to a value of 1, and the other expression values were calculated relative to this reference.

### Quantification of allelic dosage using pyrosequencing (BD2 and GEA studies)

Primers were designed using PyroMark Assay Design 2.0 software (details in Supplementary Table 11). PCR samples were produced using PyroMark PCR Kit (Qiagen). Runs were analyzed by PyroMark Q24 instrument (Qiagen). For GEA, we used a range of DNA pools as a scale with the following proportions: 0, 25, 33, 50, 66, 75, 90 and 100% of the mutant allele. These pools were obtained by mixing different amounts of purified plasmids containing either a wild-type or a R217del fragment of MITF.

### Gene set enrichment analyses on RNA sequencing data (DR study)

The RNA sequencing data considered in our analyses were obtained from Dorshorst *et al*.^41^ and are available at the following: 10.1371/journal.pone.0128969.s004. Briefly, RNA was extracted from skin samples from three dominant red and three dominant black animals. cDNA libraries were generated and sequenced as paired-end 50 base pair reads on an Illumina HiSeq. 2000 instrument. Differential expression analyses were performed using DESeq. 2 package. For details see Dorshorst *et al*.^41^. In this study, a threshold of FDR < 0.05 was used to evaluate significant expression changes between dominant red and dominant black skin samples (Supplementary Data 1). Transcript annotation was updated using Ensembl release 88 and information from the UCSC Genome Browser (http://genome.ucsc.edu/; accessed 2017/04/01). We used the “Non-cow RefSeq genes,” “Cow mRNAs from GenBank” and “Cow ESTs that have been spliced” tracks to recover the name of protein-coding genes that are predicted to code “unknown protein” according to Ensembl. Gene set enrichment analyses were carried out with two software packages using different methods and sources of information. To provide a first overview of the over-represented groups of genes, we performed two analyses with Enrichr (http://amp.pharm.mssm.edu/Enrichr/)^46, 47^ using the list of genes that are significantly upregulated (FDR < 0.05, fold change >1) and downregulated (FDR < 0.05, fold change <1). We focused on Mammalian Phenotype ontology from Mouse Genome Informatics (MGI Mammalian Phenotype Level 3 and 4) to gain information on the possible phenotypic consequences associated with modification of gene expression in dominant red versus dominant black animals. Then we performed two analyses with Ingenuity Pathway Analysis (http://www.ingenuity.com/products/ipa/) using two list of genes (FDR < 0.05 and fold change >2 or <0.5, respectively) to identify canonical pathways that are markedly upregulated or downregulated. Results of gene set enrichment analyses are presented in Supplementary Tables 8 and 9.

### Data availability

WGS data from 1146 out of the 1230 individuals used for filtering are part of the 1000 Bull Genomes Run 4.0 (Bouwman *et al*., submitted). The remaining control genomes were deposited in the European Nucleotide Archive (ENA) (http://www.ebi.ac.uk/ena) under the accession numbers: PRJEB11962, PRJEB11963, PRJEB12092, PRJEB12093, PRJEB12094, PRJEB12095, PRJEB14604, PRJEB5435, PRJEB5965, PRJEB7527, PRJEB7528, PRJEB7707, PRJEB8226, and PRJEB18113. The genome sequences of the affected animals and of Invincible (CHAFRAM001893105503, carrier of a *de novo* mutation in EDAR) were also deposited in ENA. Sample accession numbers are: SAMEA104055783 for BD1, SAMEA3869562 for the DR bull MORSAN AFTERBURN (HOLCANM000009626893), SAMEA3869561 for GEA, SAMEA3869559 for NC, and SAMEA3869563 for Invincible in project PRJEB12703; SAMEA3706814 for BD3, SAMEA3706815 for the sire, and SAMEA3706816 for the dam in project PRJEB12092; SAMEA3723253 for OI in project PRJEB12324; and SAMEA3723308 for BD2 in project PRJEB12325.

## Electronic supplementary material

Supplementary material

Supplementray Data 1

## References

[1] Simmons D (2008). The Use of Animal Models in Studying Genetic Disease: Transgenesis and Induced Mutation. Nature Education.

[2] Rodríguez-Seguí S, Akerman I, Ferrer J (2012). GATA believe it: new essential regulators of pancreas development. J. Clin. Invest..

[3] Andersson L (2013). Molecular consequences of animal breeding. Curr. Opin. Genet. Dev..

[4] Daetwyler HD (2014). Whole-genome sequencing of 234 bulls facilitates mapping of monogenic and complex traits in cattle. Nat. Genet..

[5] Li H (2009). The Sequence Alignment/Map format and SAMtools. Bioinformatics.

[6] McLaren W (2010). Deriving the consequences of genomic variants with the Ensembl API and SNP Effect Predictor. Bioinformatics.

[7] Ye K, Schulz MH, Long Q, Apweiler R, Ning Z (2009). Pindel: a pattern growth approach to detect break points of large deletions and medium sized insertions from paired-end short reads. Bioinformatics.

[8] Rausch T (2012). DELLY: structural variant discovery by integrated paired-end and split-read analysis. Bioinformatics.

[9] Thorvaldsdóttir H (2013). Integrative Genomics Viewer (IGV): high-performance genomics data visualization and exploration. Brief Bioinform..

[10] Vissing H (1989). Glycine to serine substitution in the triple helical domain of pro-alpha 1 (II) collagen results in a lethal perinatal form of short-limbed dwarfism. J. Biol. Chem..

[11] Hanley KP (2008). Ectopic SOX9 mediates extracellular matrix deposition characteristic of organ fibrosis. J. Biol. Chem..

[12] Shi S, Kirk M, Kahn AJ (1996). The role of type I collagen in the regulation of the osteoblast phenotype. J. Bone Miner. Res..

[13] Levy C, Khaled M, Fisher DE (2006). MITF: master regulator of melanocyte development and melanoma oncogene. Trends Mol. Med..

[14] Takebayashi K (1996). The recessive phenotype displayed by a dominant negative microphthalmia-associated transcription factor mutant is a result of impaired nucleation potential. Mol. Cell. Biol..

[15] Hodgkinson CA (1993). Mutations at the mouse microphthalmia locus are associated with defects in a gene encoding a novel basic-helix-loop-helix-zipper protein. Cell.

[16] Steingrímsson E (1994). Molecular basis of mouse microphthalmia (mi) mutations helps explain their developmental and phenotypic consequences. Nat. Genet..

[17] Hansdottir AG (2004). The novel mouse microphthalmia mutations Mitfmi-enu5 and Mitfmi-bcc2 produce dominant negative Mitf proteins. Genomics.

[18] Philipp U (2011). A MITF mutation associated with a dominant white phenotype and bilateral deafness in German Fleckvieh cattle. PLoS One.

[19] Hauswirth R (2012). Mutations in MITF and PAX3 cause “splashed white” and other white spotting phenotypes in horses. PLoS Genet..

[20] Léger S (2012). Novel and recurrent non-truncating mutations of the MITF basic domain: genotypic and phenotypic variations in Waardenburg and Tietz syndromes. Eur. J. Hum. Genet..

[21] Bajpai R (2010). CHD7 cooperates with PBAF to control multipotent neural crest formation. Nature.

[22] Pagon RA, Graham JM, Zonana J, Yong SL (1981). Coloboma, congenital heart disease, and choanal atresia with multiple anomalies: CHARGE association. J. Pediatr..

[23] Blake KD (1998). CHARGE association: an update and review for the primary pediatrician. Clin. Pediatr. (Phila.).

[24] Amiel J (2001). Temporal bone anomaly proposed as a major criteria for diagnosis of CHARGE syndrome. Am. J. Med Genet..

[25] Verloes A (2005). Updated diagnostic criteria for CHARGE syndrome: a proposal. Am. J. Med. Genet..

[26] Jongmans MC (2006). CHARGE syndrome: the phenotypic spectrum of mutations in the CHD7 gene. J. Med. Genet..

[27] Lalani SR (2006). Spectrum of CHD7 mutations in 110 individuals with CHARGE syndrome and genotype-phenotype correlation. Am. J. Hum. Genet..

[28] Vuorela P (2007). Molecular analysis of the CHD7 gene in CHARGE syndrome: identification of 22 novel mutations and evidence for a low contribution of large CHD7 deletions. Genet. Med..

[29] Delahaye A (2007). Familial CHARGE syndrome because of CHD7 mutation: clinical intra- and interfamilial variability. Clin. Genet..

[30] Vuorela PE (2008). A familial CHARGE syndrome with a CHD7 nonsense mutation and new clinical features. Clin. Dysmorphol..

[31] Hughes SS, Welsh HI, Safina NP, Bejaoui K, Ardinger HH (2014). Family history and clefting as major criteria for CHARGE syndrome. Am. J. Med. Genet..

[32] Lachmann A (2010). ChEA: transcription factor regulation inferred from integrating genome-wide ChIP-X experiments. Bioinformatics.

[33] Rouillard, A.D. *et al*. The harmonizome: a collection of processed datasets gathered to serve and mine knowledge about genes and proteins. *Database* (2016). 10.1093/database/baw100 PMC4930834 27374120

[34] Radice GL (1997). Developmental defects in mouse embryos lacking N-cadherin. Dev. Biol..

[35] Kadowaki M (2007). N-cadherin mediates cortical organization in the mouse brain. Dev. Biol..

[36] Li J (2008). N-cadherin haploinsufficiency affects cardiac gap junctions and arrhythmic susceptibility. J Mol. Cell. Cardiol..

[37] Letourneur F (1994). Coatomer is essential for retrieval of dilysine-tagged proteins to the endoplasmic reticulum. Cell.

[38] Jackson LP (2012). Molecular basis for recognition of dilysine trafficking motifs by COPI. Dev. Cell.

[39] Coutinho P (2004). Differential requirements for COPI transport during vertebrate early development. Dev. Cell.

[40] Xu X (2010). Mutation in archain 1, a subunit of COPI coatomer complex, causes diluted coat color and Purkinje cell degeneration. PLoS Genet..

[41] Dorshorst B (2015). Dominant Red coat color in Holstein cattle is associated with a missense mutation in the Coatomer Protein Complex, subunit Alpha (COPA) gene. PLoS One.

[42] Capitan, A. *et al*. Rapid discovery of mutations responsible for sporadic dominant genetic defects in livestock using genome sequence data: Enhancing the value of farm animals as model species. *Proc. 10th World Congr. Genet. Appl. Livest. Sci. Commun*. **182**, Oral presentation available at https://asas.confex.com/asas/WCGALP14/flvgateway.cgi/id/5159?recordingid=5159 (2014).

[43] Watkin LB (2015). COPA mutations impair ER-Golgi transport and cause hereditary autoimmune-mediated lung disease and arthritis. Nat. Genet..

[44] Vece TJ (2016). Copa syndrome: a novel autosomal dominant immune dysregulatory disease. J. Clin. Immunol..

[45] Northrop, R. B., Connor, A. N. In Neuman, M. R. (ed.) Introduction to Molecular Biology, Genomics and Proteomics for Biomedical Engineers, p. 37. CRC Press Taylor and Francis Group, Boca Raton (2009).

[46] Chen EY (2013). Enrichr: interactive and collaborative HTML5 gene list enrichment analysis tool. BMC Bioinformatics.

[47] Kuleshov MV (2016). Enrichr: a comprehensive gene set enrichment analysis web server 2016 update. Nucleic Acids Res..

[48] Baguma-Nibasheka M, Kablar B (2008). Pulmonary hypoplasia in the connective tissue growth factor (Ctgf) null mouse. Dev. Dyn..

[49] Gründer (2002). Nuclear factor I-B (Nfib) deficient mice have severe lung hypoplasia. Mech. Dev..

[50] Ramirez MI (2003). T1alpha, a lung type I cell differentiation gene, is required for normal lung cell proliferation and alveolus formation at birth. Dev. Biol..

[51] Saadoun D, Terrier B, Cacoub P (2011). Interleukin-25: key regulator of inflammatory and autoimmune diseases. Curr. Pharm. Des..

[52] Molofsky AB, Savage AK, Locksley RM (2015). Interleukin-33 in tissue homeostasis, injury, and inflammation. Immunity.

[53] Parks WC, Wilson CL, López-Boado YS (2004). Matrix metalloproteinases as modulators of inflammation and innate immunity. Nat. Rev. Immunol..

[54] Burkly L (1995). Expression of relB is required for the development of thymic medulla and dendritic cells. Nature.

[55] Valéro R (2002). A defective NF-kappa B/RelB pathway in autoimmune-prone New Zealand black mice is associated with inefficient expansion of thymocyte and dendritic cells. J. Immunol..

[56] Chang CY (2013). NFIB is a governor of epithelial-melanocyte stem cell behaviour in a shared niche. Nature.

[57] Masui Y (2011). A missense mutation in the death domain of EDAR abolishes the interaction with EDARADD and underlies hypohidrotic ectodermal dysplasia. Dermatology.

[58] Sadier A, Viriot L, Pantalacci S, Laudet V (2014). The ectodysplasin pathway: from diseases to adaptations. Trends Genet..

[59] Li H, Durbin R (2009). Fast and accurate short read alignment with Burrows-Wheeler Transform. Bioinformatics.

[60] Boichard D (2012). Genomic Selection in French Dairy Cattle. Anim. Prod. Sci..

[61] Sargolzaei M, Chesnais JP, Schenkel FS (2014). A new approach for efficient genotype imputation using information from relatives. BMC Genomics.

[62] Weckx S (2005). NovoSNP, a novel computational tool for sequence variation discovery. Genome Res..

[63] Lander ES, Botstein D (1987). Homozygosity mapping: a way to map human recessive traits with the DNA of inbred children. Science.

[64] Charlier C (2008). Highly effective SNP-based association mapping and management of recessive defects in livestock. Nat. Genet..

[65] Boichard, D. Pedig: a Fortran package for pedigree analysis suited to large populations. 7th World Congress on Genetics Applied to Livestock Production, Montpellier, August 19–23 2002, paper 28-13 (2002).

[66] Joerg H, Fries HR, Meijerink E, Stranzinger GF (1996). Red coat color in Holstein cattle is associated with a deletion in the MSHR gene. Mamm. Genome.

