# Supplementary material for: Rapid Discovery of De Novo Deleterious Mutations in Cattle Enhances the Value of Livestock as Model Species
Source: Sci Rep. 2017 Sep 13;7:11466. doi: 10.1038/s41598-017-11523-3 (PMC5597596; doi:10.1038/s41598-017-11523-3)
Supplement: Supplementary file 1 — Supplementary material [file 41598_2017_11523_MOESM1_ESM.pdf]

Supplementary Material

Rapid Discovery of De Novo Deleterious Mutations in Cattle Enhances the Value of Livestock as Model Species

E. Bourneuf[1,2,§], P. Otz[3,§], H. Pausch[4], V. Jagannathan[5], P. Michot[1,6], C. Grohs[1], G. Piton[1,2], S. Ammermüller[4], M.-C. Deloche[1,6], S. Fritz[1,6], H Leclerc[1,7], C. Péchoux[1,8], A. Boukadiri[1], C. Hozé[1,6], R. Saintilan[1,6], F. Créchet[1,2], M Mosca[9], D. Segelke[10], F. Guillaume[1], S. Bouet[1], A. Baur[1,6], A. Vasilescu[11], L. Genestout[11], A. Thomas[12], A. Allais-Bonnet[1,6], D. Rocha[1], M.-A. Colle[13,14], C. Klopp[15], D. Esquerré[16], C. Wurmser[4], K. Flisikowski[17], H. Schwarzenbacher[18], J. Burgstaller[19], M. Brügmann[20], E. Dietschi[5], N. Rudolph[21], M. Freick[22], S. Barbey[23], G. Fayolle[24], C. Danchin-Burge[7], L. Schibler[6], B. Bed'Hom[1], B.J. Hayes[25,26], H. D. Daetwyler[25,27], R. Fries[4], D. Boichard[1], D. Pin[9], C. Drögemüller[5], A. Capitan[1,6,*]

Table of contents

Supplementary Fig.1	3-4
Supplementary Fig.2	5-6
Supplementary Fig.3	7-8
Supplementary Fig.4	9-10
Supplementary Fig.5	11-12
Supplementary Fig.6	13
Supplementary Fig.7	14
Supplementary Fig.8	15-16
Supplementary Fig.9	17-18
Supplementary Fig.10	19
Supplementary Fig.11	20
Supplementary Fig.12	21
Supplementary Fig.13	22
Supplementary Fig.14	23
Supplementary Fig.15	24
Supplementary Fig.16	25
Supplementary Fig.17	26
Supplementary Fig.18	27
Supplementary Fig.19	28
Supplementary Table 1	29
Supplementary Table 2	30-33
Supplementary Table 3	34
Supplementary Table 4	34
Supplementary Table 5	36
Supplementary Table 6	36
Supplementary Table 7	36
Supplementary Table 8	37
Supplementary Table 9	38
Supplementary Table 10	39
Supplementary Table 11	40
Supplementary Table 12	41
Supplementary Note 1	42-44
Supplementary Note 2	45-46
Supplementary Data 1 (legend only)	46
References	47-49

Chr22 g.31746506_31746508del

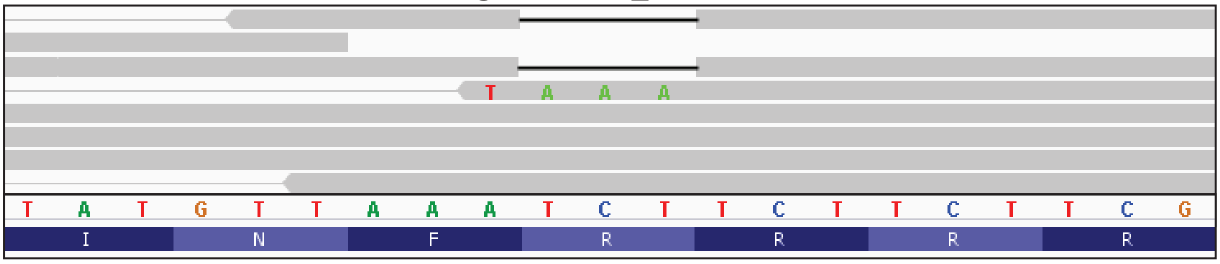

a

MITF p.R211del

Chr3. g.9479761C>T

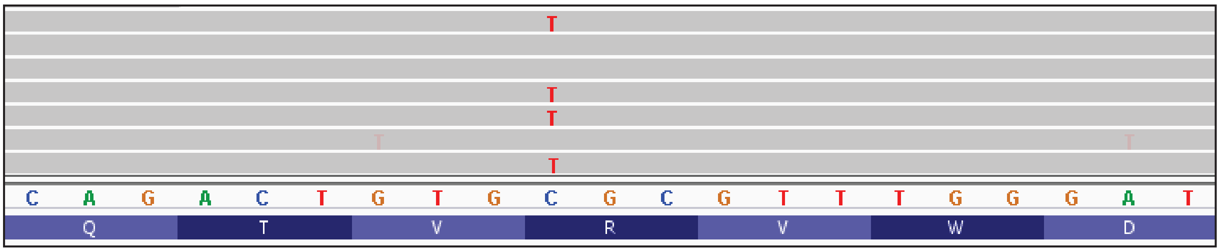

b

COPA p.R160C

Chr14 g.28085731_28085735del

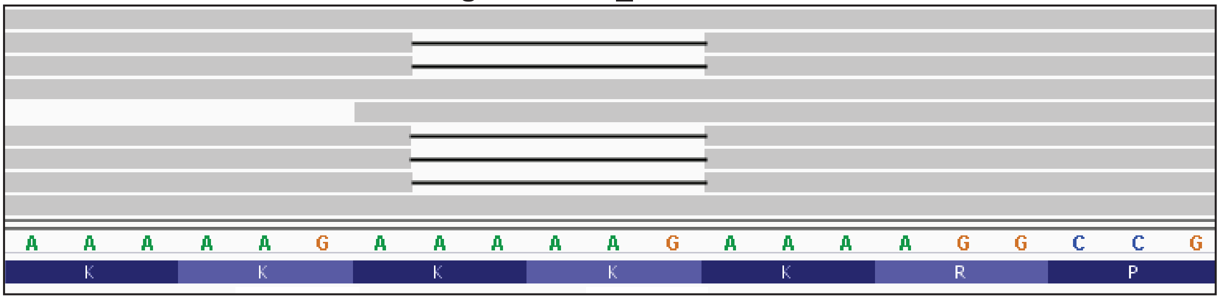

c

CHD7 p.K594AfsX29

Chr19 g.37101299_37101302delinsT

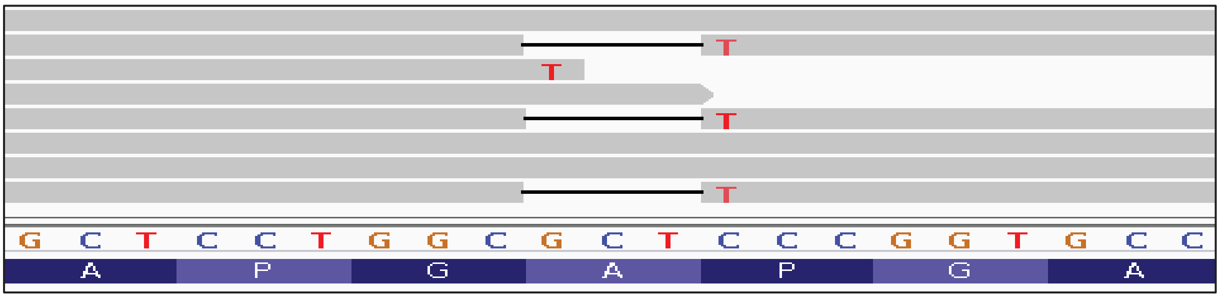

d

COL1A1 p.A1049_P1050DelInsS

Chr5 g.32469820G>A

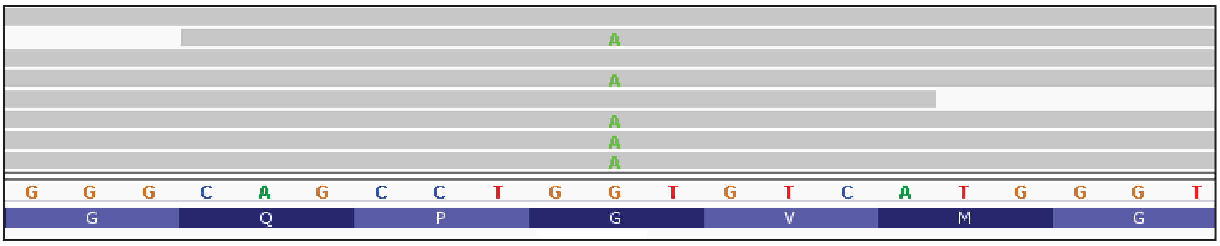

e

COL2A1 p.G600D

Chr5 g.32476082G>A

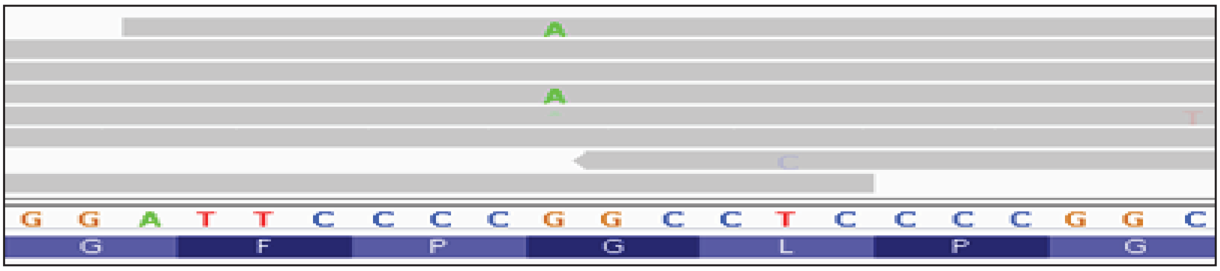

f

COL2A1 p.G996S

Chr5 g. 32471813G>A

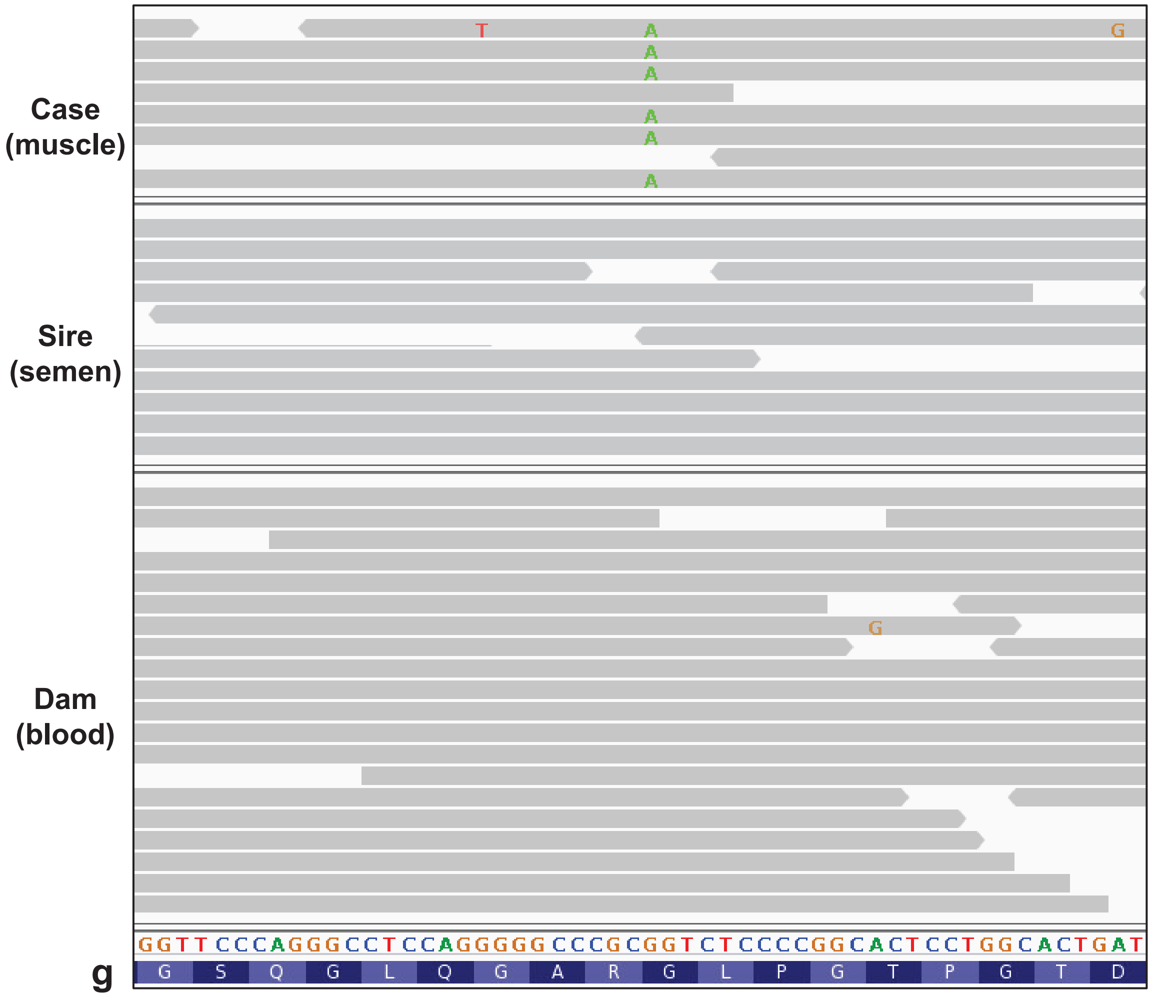

g

COL2A1 p.G720S

Supplementary Fig . 1: IGV snapshot showing the candidate mutations for the seven dominant conditions studied.

Candidate mutations for glass-eyed albino (GEA, **a**), dominant red (DR, **b**), a neurocristopathy (NC, **c**), osteogenesis imperfect (OI, **d**) and the three bulldog calf syndromes (BD1, **e**; BD2, **f**; BD3, **g**) are presented together with their predicted consequences at the protein level. Note that candidate mutations for GEA and NC affect repeated motifs (TCT and AAAAAG/AAAAG respectively) which are known to display a higher mutation rate than the average. In **g**) the tissue of origin of the DNA samples is detailed between bracket.

a

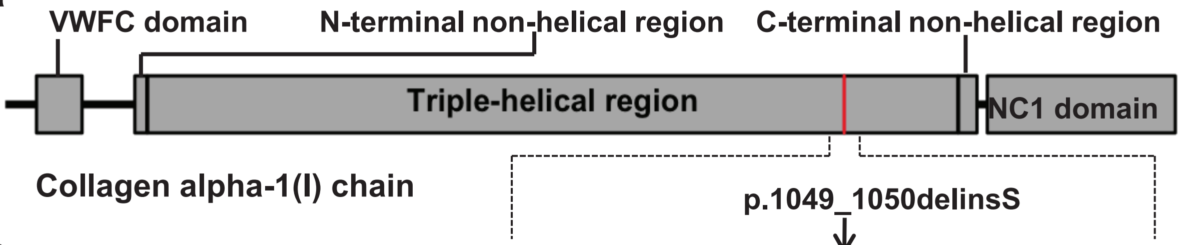

b

```
Bos taurus              GDRGETGPAGPPGAPGAPGAPGPVGPAGKSGDR
Homo sapiens            GDRGETGPAGPPGAPGAPGAPGPVGPAGKSGDR
Anolis carolinensis     GDRGETGPAGPPGAPGAPGAPGPIGPAGKNGDR
Xenopus tropicalis      GDRGEGGPAGPPGAPGAPGAPGPVGPAGKSGDR
Danio rerio             GDRGETGPSGTPGAPGPPGAAGPIGPAGKTGDR
                        ***** **:* ***** *** **:*****.***
```

c

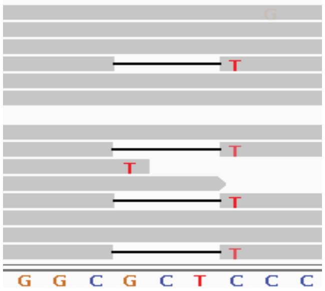

d

Phenotype	Number
Stillborn or euthanized	140 (31.67%)
Normal	302 (68.33%)
Total	442

e

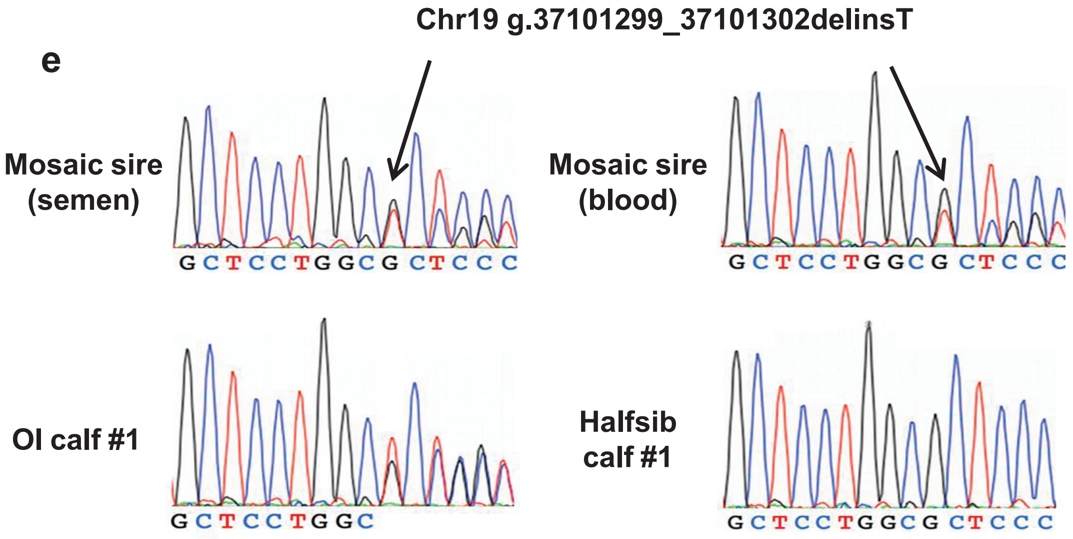

f

Animals	N	g.37101299_ 37101302delinsT
Sire	1	Mosaic
OI calves	7	GCTC/**T---**
Dam of OI calves	2	GCTC/GCTC
Wt halfsib calves	19	GCTC/GCTC
Wt halfsib calf #12	1	Chimeric

Supplementary Fig. 2: Molecular characterization of the candidate mutation for an osteogenesis imperfecta (OI) syndrome in bovine.

Supplementary Fig. 2: Molecular characterization of the candidate mutation for an osteogenesis imperfecta (OI) syndrome in bovine.

(**a**) Domain and region information for the α1 chain of type I collagen obtained from the UniProt database (http://www.uniprot.org/; accession number: P02453). (**b**) Multispecies alignment of the COL1A1 proteins showing a high conservation of residues A1049 and P1050 among vertebrates. Protein sequence accession numbers (from NCBI) for each species are NP_001029211.1 (Bos taurus), NP_000079.2 (Homo sapiens), NP_001011005.1 (Xenopus tropicalis), XP_003222687.1 (Anolis carolinensis) and NP_954684.1 (Danio rerio). Note that the replacement of two amino acids (alanine and proline) by a single one (serine) breaks the repetition of the Gly-x-y triplets, which is typical of triple-helical regions from collagen proteins. (**c**) IGV snapshot showing the Chr19 g.37101299_37101302delinsT mutation in Halvar PP. This sire was sequenced to an average read depth of 20.7 x. The proportion of reads carrying the g.37101299_37101302delinsT variant (5/14 =35.7%) is close to the proportion of stillborn or euthanized calves (31.7%) in its progeny (**d**) and supports somatic mosaicism in the bull. (**e**) Electrophoregrams showing somatic mosaicism in Halvar PP as compared with one OI affected and one unaffected progeny. DNA from Halvar PP was extracted from blood and semen samples. (**f**) Results of the genotyping by PCR and Sanger sequencing of seven affected (OI) calves, twenty unaffected (Wt) paternal halfsibs, their unaffected sire (Halvar PP) and the dam of an OI calf for the candidate polymorphism (Chr19 g.37101299_37101302delinsT). The deletion variant which is predicted to cause the COL1A1 p. 1049_1050delinsS mutation showed a perfect genotype phenotype correlation among Halvar's progeny with the exception of one unaffected calf which was found to be a chimera (**Supplementary Fig. 8 and 9**).

a

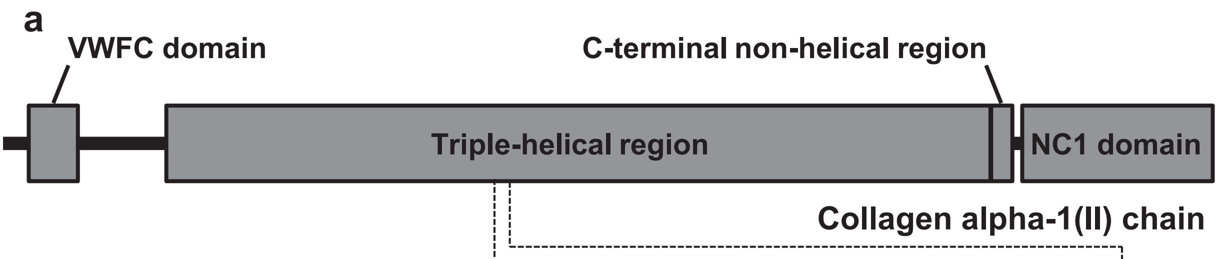

b

p.G600D

Bos taurus	GRPGPPGPQGARGQP**G**VMGFPGPKGANGEPGKA
Homo sapiens	GRPGPPGPQGARGQP**G**VMGFPGPKGANGEPGKA
Gallus gallus	GRPGPPGPQGARGQP**G**VMGFPGPKGANGEPGKA
Anolis carolinensis	GRPGPPGPQGARGQP**G**VMGFPGPKGANGEAGKA
Xenopus tropicalis	GRPGPPGPQGARGQP**G**VMGFPGPKGANGEPGKA
Danio rerio	GRPGPPGPLGARGQP**G**VMGFPGPKGANGEPGKP
	******** ********************.**.

c

Animal	g.32469660C>G	g.32469820G>A
Sire	C/G	G/G
BD calf #1	C/G	G/**A**
Dam of calf #1	C/G	G/G
BD calf #2	G/G	G/**A**
Dam of calf #2	G/G	G/G
Wt halfsib calf #3	C/C	G/G
Dam of calf #3	C/G	G/G
Wt halfsib calf #4	C/G	G/G
Dam of calf #4	G/G	G/G
Wt halfsib calf #5	C/G	G/G
Dam of calf #5	C/G	G/G
Wt halfsib calf #6	G/G	G/G
Dam of calf #6	G/G	G/G
Wt halfsib calf #7	C/G	G/G
Dam of calf #7	C/C	G/G
Wt halfsib calf #8	G/G	G/G
Dam of calf #8	G/G	G/G

e

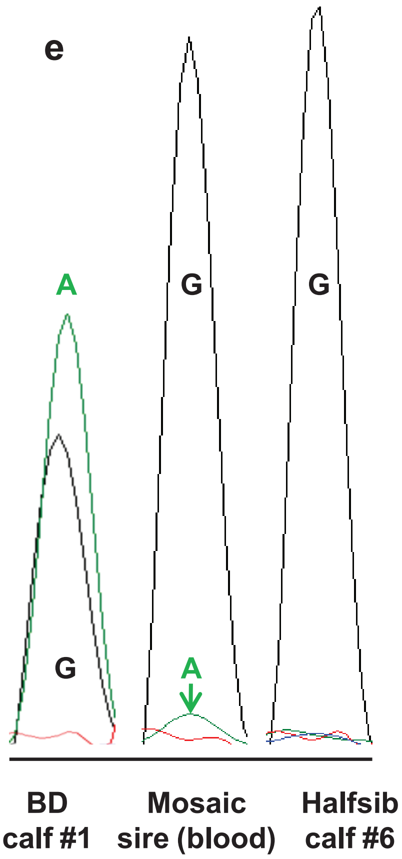

d

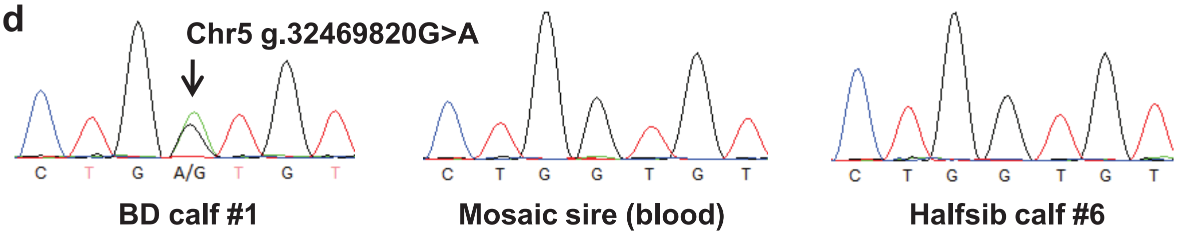

Supplementary Fig. 3: Molecular characterization of the candidate mutation for an achondrogenesis type 2 syndrome in bovine (Bulldog calf syndrome #1).

Supplementary Fig. 3: Molecular characterization of the candidate mutation for an achondrogenesis type 2 syndrome in bovine (Bulldog calf syndrome #1).

(**a**) Domain and region information for the α1 chain of type II collagen obtained from the UniProt database (http://www.uniprot.org/; accession number: P02459). (**b**) Multispecies alignment of the COL2A1 proteins from different species showing a complete conservation of residue G600 among vertebrates. Protein sequences accession numbers in Ensembl for each species are ENSBTAP00000017505 (Bos taurus), ENSGALP00000035064 (Gallus gallus), ENSXETP00000043834 (Xenopus tropicalis), ENSACAP00000006225 (Anolis carolinensis), ENSDARP00000091007 (Danio rerio) and ENSP00000369889 (Homo sapiens). Note the repetition of Gly-x-y triplet which is typical of triple-helical regions from collagen proteins. (**c**) Results of the genotyping by PCR and Sanger sequencing of two affected (BD) calves, six unaffected (Wt) paternal halfsibs, their unaffected dams and their unaffected sire for the candidate polymorphism (Chr5 g.32469820A>G) and for a neighbouring polymorphism (Chr5 g.32469660C>G). Allele g.32469820A, which is predicted to cause the deleterious COL2A1 p.G600D substitution, is carried only by the two affected calves and none of their parents suggesting that (i) the mutation occurred de novo in the sires's germline or (ii) that the sire was affected by somatic mosaicism. Analysis of genotyping data from both BD calves reveals that allele g.32469820A is associated with allele g.32469660G. The fact that several of their halfsibs (e.g. calves #6 and #8) received the same paternal chromosome but without the g.32469820A *de novo* mutation, and are unaffected further support the causality of the mutation. (d) Electrophoregrams of BD calf #2, its unaffected halfsib calf #6 and their sire. (e) Magnification of nucleotide g.32469820 revealing mosaicism in DNA extracted from the blood of the sire (i.e. somatic mosaïcism). Remarkably the ratio "size of pike A/(size of pike A + size of pike G)" is equal to 4,5% which is very close to the proportion of affected calves observed in its progeny (5/114).

a

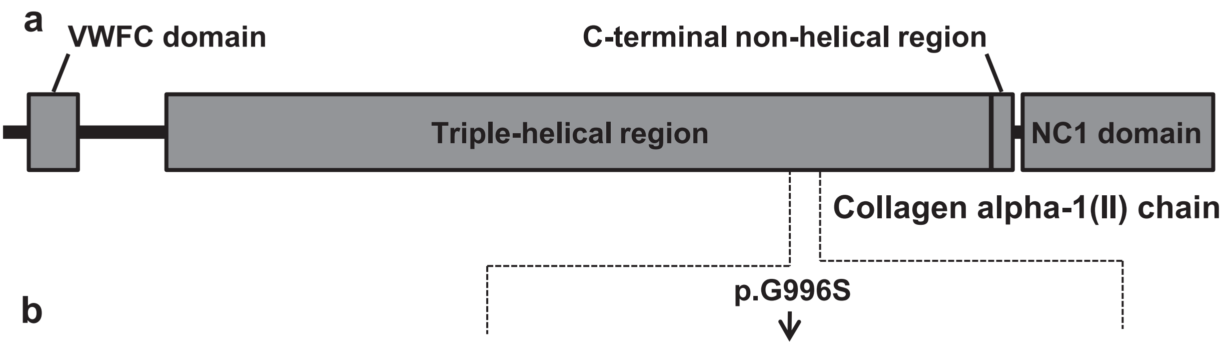

b

```
Bos taurus             GIVGLPGQRGERGFPGLPGPSGEPGKQGAPGAS
Homo sapiens           GIVGLPGQRGERGFPGLPGPSGEPGKQGAPGAS
Gallus gallus          GIVGLPGQRGERGFPGLPGPSGEPGKQGAPGSA
Anolis carolinensis    GIVGLPGQRGERGFPGLPGPSGEPGKQGASGGP
Xenopus tropicalis     GIVGLPGQRGERGFPGLPGPSGEPGKQGAPGGS
Danio rerio            GIVGLPGQRGERGFPGLPGPSGEPGKQGAPGGS
                       ***************************** *.
```

c

Animal	g.32476082G>A
Paternal grandsire	G/G
Sire (Energy P)	G/G

Progeny	N	G/G	G/A
Bulldog	10	-	10
Normal	58	58	-

d

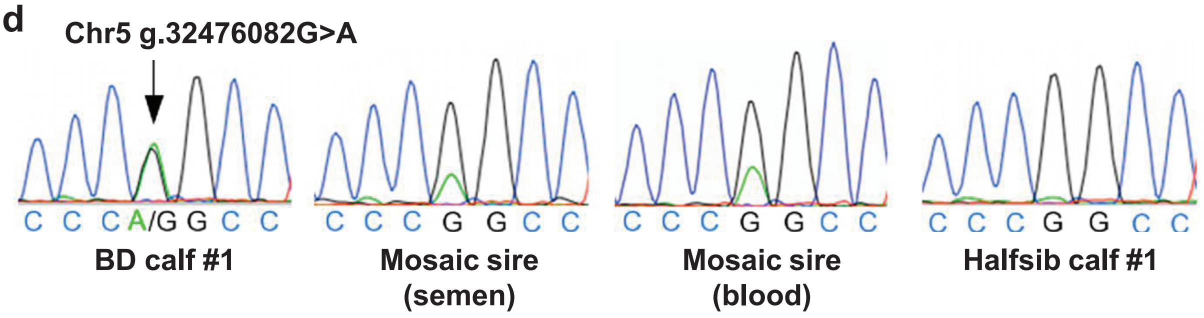

e

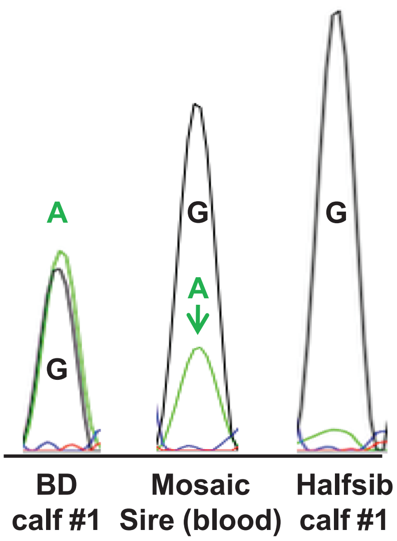

f

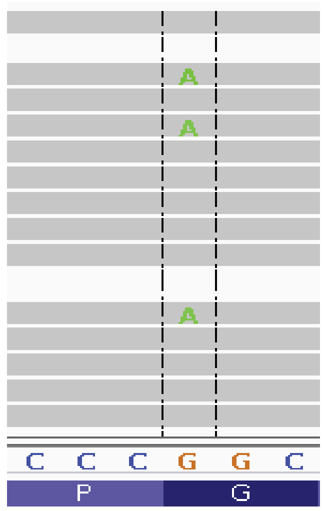

g

Progeny	Number
Stillborn	57 (20.7%)
Normal	218 (79.3%)
Total	275

Supplementary Fig. 4: Molecular characterization of the candidate mutation for an achondrogenesis type 2 syndrome in bovine (Bulldog calf syndrome #2).

Supplementary Fig. 4: Molecular characterization of the candidate mutation for an achondrogenesis type 2 syndrome in bovine (Bulldog calf syndrome #2).
(**a**) Domain and region information for the α1 chain of type II collagen obtained from the UniProt database (http://www.uniprot.org/; accession number: P02459). (**b**) Multispecies alignment of the COL2A1 proteins showing a complete conservation of residue G996 among vertebrates. Protein sequences accession numbers in Ensembl for each species are ENSBTAP00000017505 (*Bos taurus*), ENSGALP00000035064 (*Gallus gallus*), ENSXETP00000043834 (*Xenopus tropicalis*), ENSACAP00000006225 (*Anolis carolinensis*), ENSDARP00000091007 (*Danio rerio*) and ENSP00000369889 (*Homo sapiens*). Note the repetition of the Gly-x-y triplet, which is typical of triple-helical regions from collagen proteins. (**c**) Results of the genotyping by PCR and Sanger sequencing of ten affected (BD) calves, 58 unaffected (Wt) paternal halfsibs, their unaffected sire (Energy P) and paternal grandsire (Earnhardt P) for the candidate polymorphism (Chr5 g.32476082G>A). Allele g.32476082A, which is predicted to cause the COL2A1 p.G996S substitution, is carried only by the ten affected calves suggesting that the sire was affected by somatic mosaicism. (**d**) Electropherograms of BD calf #1, its unaffected halfsib calf #1 and their mosaic sire Energy P. DNA from Energy P was extracted from blood and semen samples. (**e**) Magnification of nucleotide g.32476082 revealing mosaicism in DNA extracted from the blood of the sire (*i.e.* somatic mosaicism). (**f**) IGV snapshot showing the g.32476082-mutation in the mosaic sire. The mosaic sire was sequenced to an average read depth of 20.0 x. The proportion of reads carrying the g.32476082A-variant (21.4%) is very close to the proportion of stillborn progeny (20.7%) (**g**). Allele quantification using pyrosequencing revealed a frequency of the mutant A allele of 22% and 32% in DNA extracted from semen and blood, respectively.

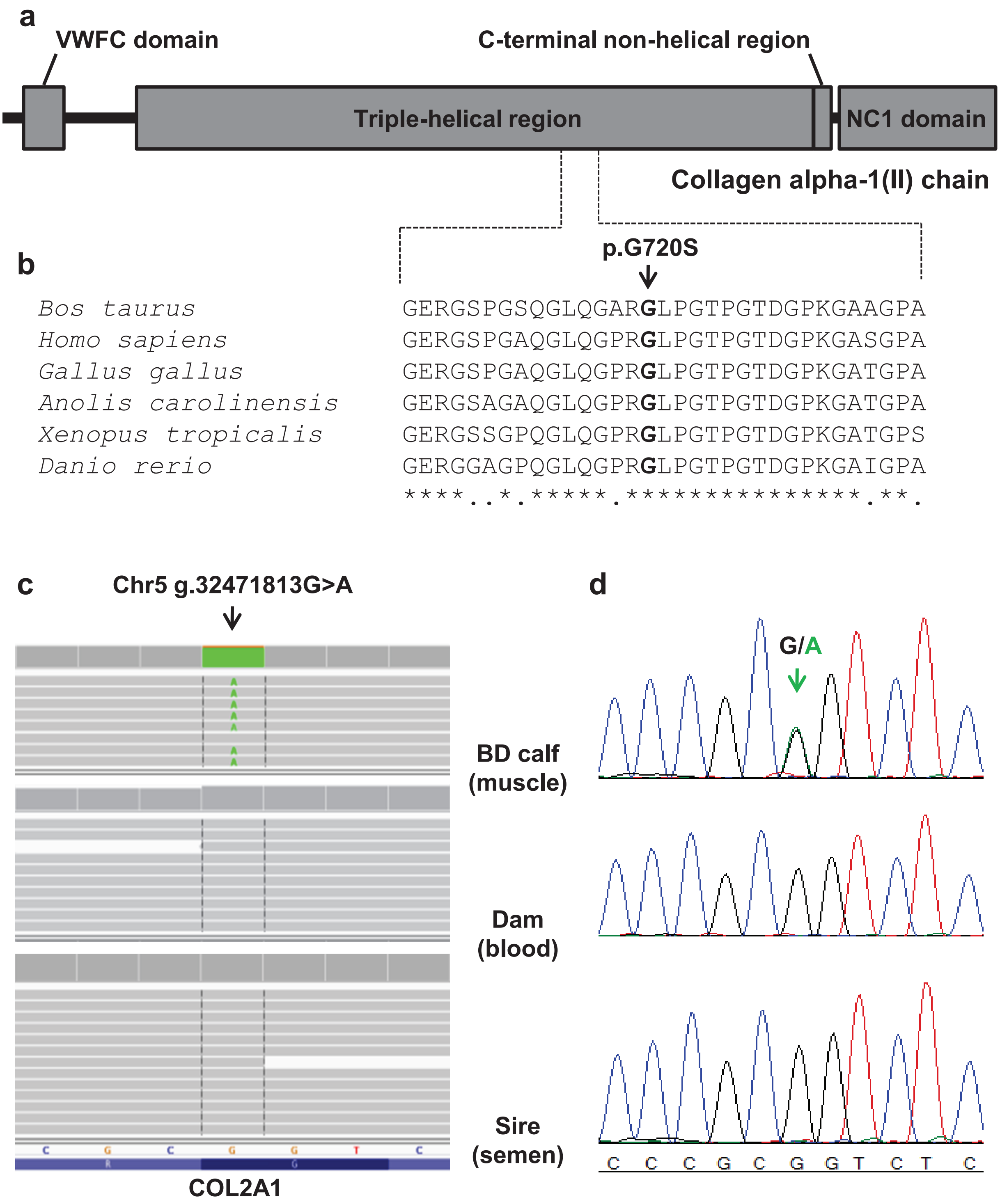

Supplementary Fig. 5: Molecular characterization of the candidate mutation for an achondrogenesis type 2 syndrome in bovine (Bulldog calf syndrome #3).

Supplementary Fig. 5: Molecular characterization of the candidate mutation for an achondrogenesis type 2 syndrome in bovine (Bulldog calf syndrome #3).

(**a**) Domain and region information for the α1 chain of type II collagen obtained from the UniProt database (http://www.uniprot.org/; accession number: P02459). (**b**) Multispecies alignment of the COL2A1 proteins from different species showing a complete conservation of residue G720 among vertebrates. Protein sequences accession numbers in Ensembl are ENSBTAP00000017505, ENSP00000369889, ENSGALP00000035064, ENSACAP00000006225, ENSXETP00000043834 and ENSDARP00000091007. Note the repetition of Gly-x-y triplet which is typical of triple-helical regions from collagen proteins. (**c**) IGV snapshot showing the g.32471813G>A mutation in the affected calf. The three genomes were sequenced to an average read depth of 15.1x (BD affected calf), 15.8x (sire), and 16.2x (dam). Note that in both parents all sequence reads carry the wild type G-allele. (**d**) Electropherograms of the BD affected calf and its sire and dam. Note that there is no indication for somatic mosaicism in DNA extracted from the semen of the sire and the blood of the dam which suggests that the mutation affected a very limited population of germ cells from one of the parents.

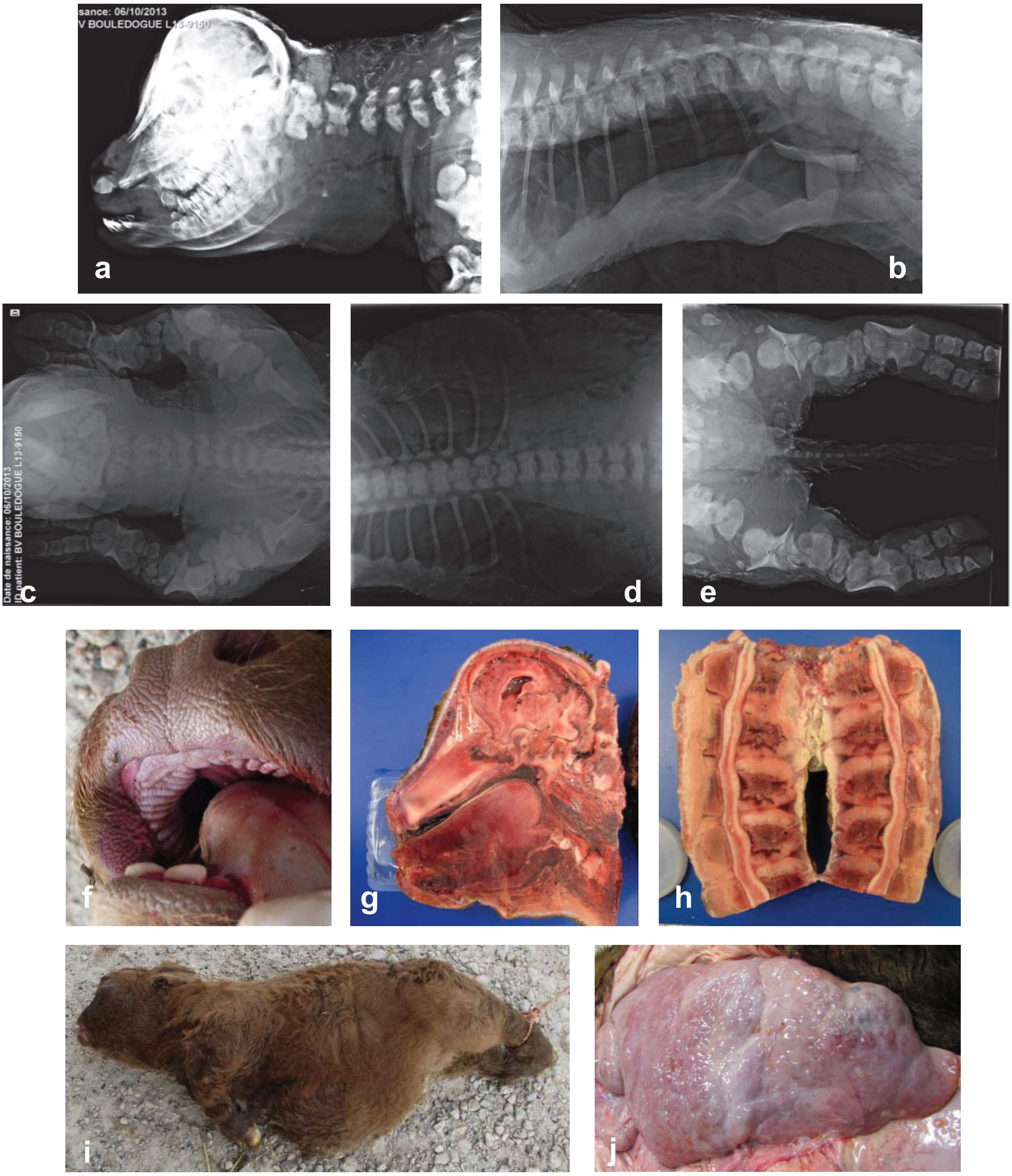

Supplementary Fig. 6: Phenotypic characteristics of an achondrogenesis type 2 syndrome in bovine caused by a COL2A1 p.G600D substitution (Bulldog calf syndrome #1).

(**a**, **b**, **c**, **d**, **e**) Radiographs of a BD calf heterozygous for a COL2A1 p.G600D mutation showing symptoms similar to calves heterozygous for a COL2A1 p.G960R mutation[4] and, on a general manner, to humans affected by achondrogenesis type 2. (**f**) Picture of an affected calf with cleft palate. (**g**) Longitudinal section of the head showing a dysplastic splanchnocranium (**h**) Longitudinal section of the spinal cord in the lumbar region showing medullary canal stenosis. (**i**) External appearance of the calf which was necropsied. Note the belly distended by the accumulation of ascite in this particular case. (**j**) External view of its fibrotic liver.

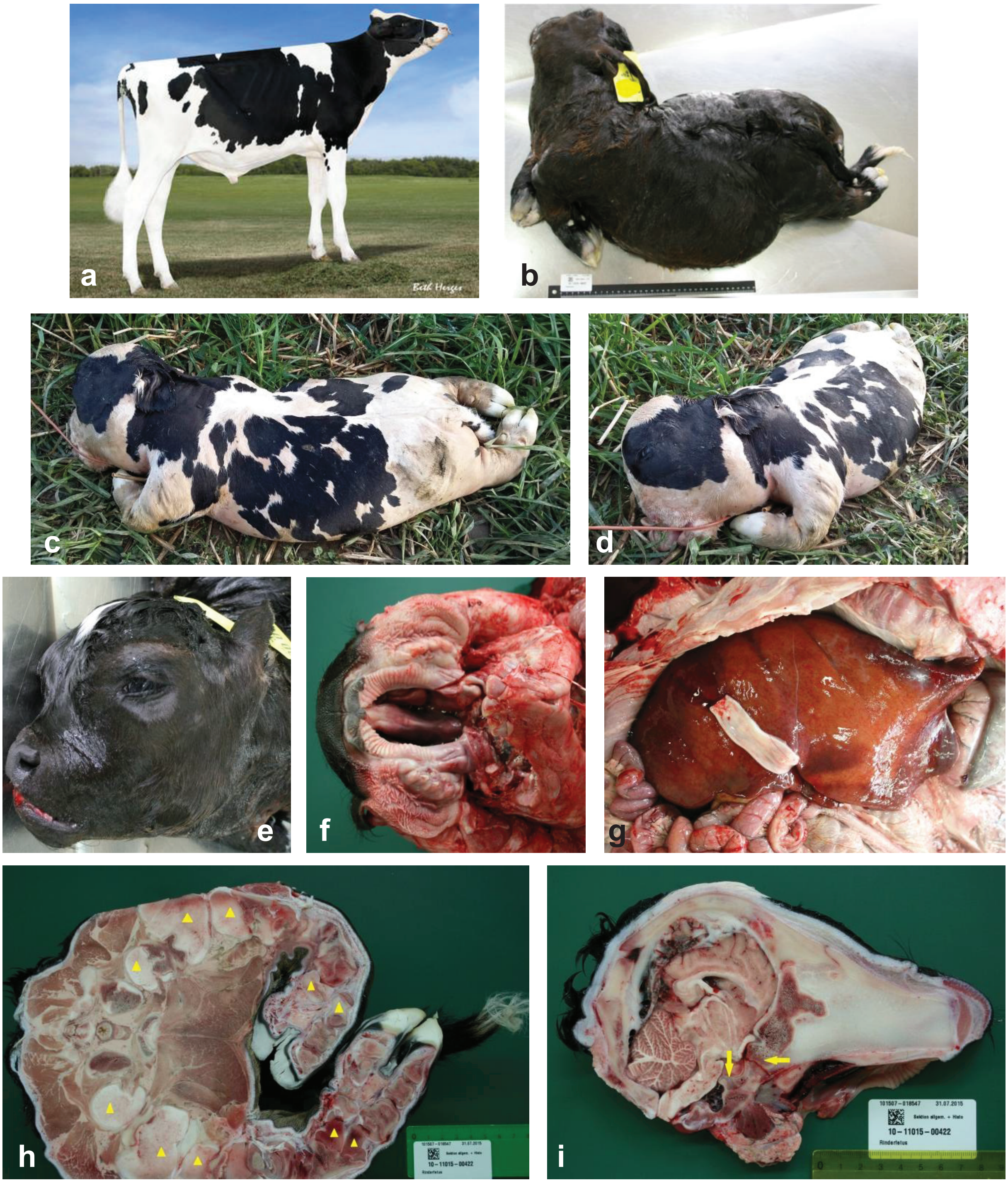

Supplementary Fig. 7: Phenotypic characteristics of an achondrogenesis type 2 syndrome in bovine caused by a COL2A1 p.G996S substitution (Bulldog calf syndrome #2).

(**a**) Photograph of Energy P (mosaic sire) at the age of eleven months. (**b**,**c**,**d**,**e**) Two progeny of Energy P with chondrodysplasia. Note the extremely short limbs and wide heads of both calves. (**f**) Cleft palate (palatoschisis) of an affected animal. (**g**) External view of the liver indicating congenital liver fibrosis (mottled liver). (**h**) Cross-section of the pelvic and hind limbs of an affected calf. Arrowheads indicate the irregular columnar cartilage proliferation resulting in an impaired bone growth in length. (**i**) Longitudinal section of the head. Arrows indicate pathological aberrations of the epiphyseal plates of the skull head.. Figures **a**,**c**,**d** were kindly provided by Masterrind GmbH, Verden.

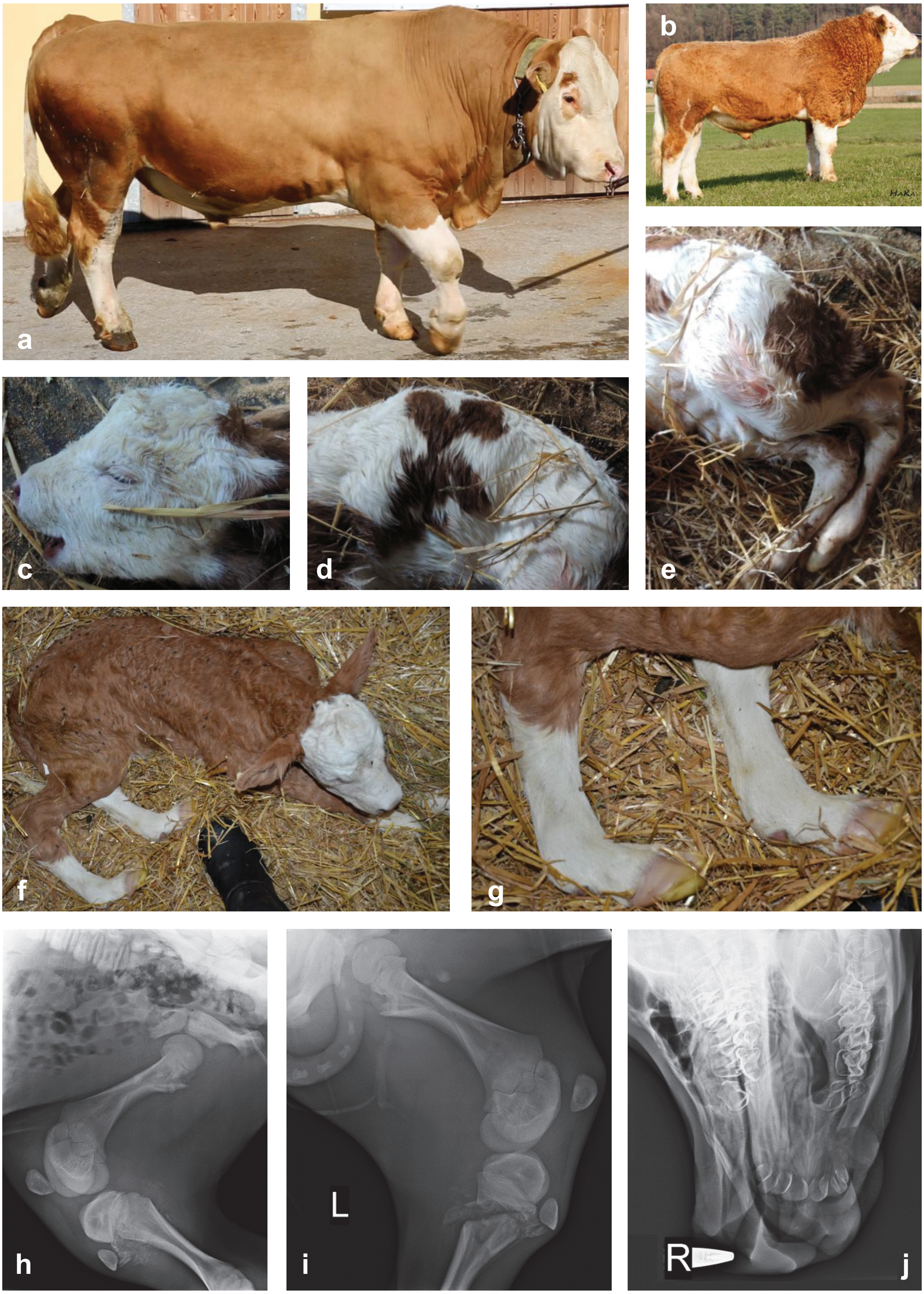

Supplementary Fig. 8: Phenotypic characteristics of osteogenesis imperfecta caused by the COL1A1 p. 1049_1050delinsS mutation.

Supplementary Fig. 8: Phenotypic characteristics of osteogenesis imperfecta caused by the COL1A1 p. 1049_1050delinsS mutation.
Photographs of Halvar PP (mosaic sire) at the age of 3.5 (**a**) and 1.5 (**b**) years, respectively. (**c,d,e**) External views of a calf that was heterozygous for the g.37101299_37101302delinsT variant on Chromosome 19 causing the COL1A1 p. 1049_1050delinsS mutation. Pathological examination revealed multiple fractures of the ribs and front and hind limbs as well as in the diastema. Bone callus formation at some fractures indicated that the injuries occurred intrauterine. Brachygnatia was observed (**c**) as well as scoliosis of the spinal column (**d**) and a severe joint laxity of the hind limbs (**e**). (**f,g**) A progeny of Halvar PP with severe hind limb malformations. (**h,i,j**) Radiographs of another calf of Halvar PP with osteogenesis imperfecta that were kindly provided by the clinical unit of diagnostic imaging of the Veterinary University Vienna. (**h**) Medio-lateral view of the proximal right hind limb. Note the several bony fragments surrounding the fracture zone and the slight displacement between the proximal tibial epiphysis and the tibial metaphysis at the level of the proximal epiphyseal growth plate of the tibia. (**i**) Medio-lateral view of the proximal left hind limb showing several bony fragments surrounding the fracture zone. (**j**) Oblique dorso-ventral view of mandibular fractures in the left and right diastema showing the fracture zone of the left diastema with diffuse coarse grained bone particles and absent bone contours. Figures **a**-**e** were kindly provided by Genostar Rinderbesamung GmbH, Gleisdorf.

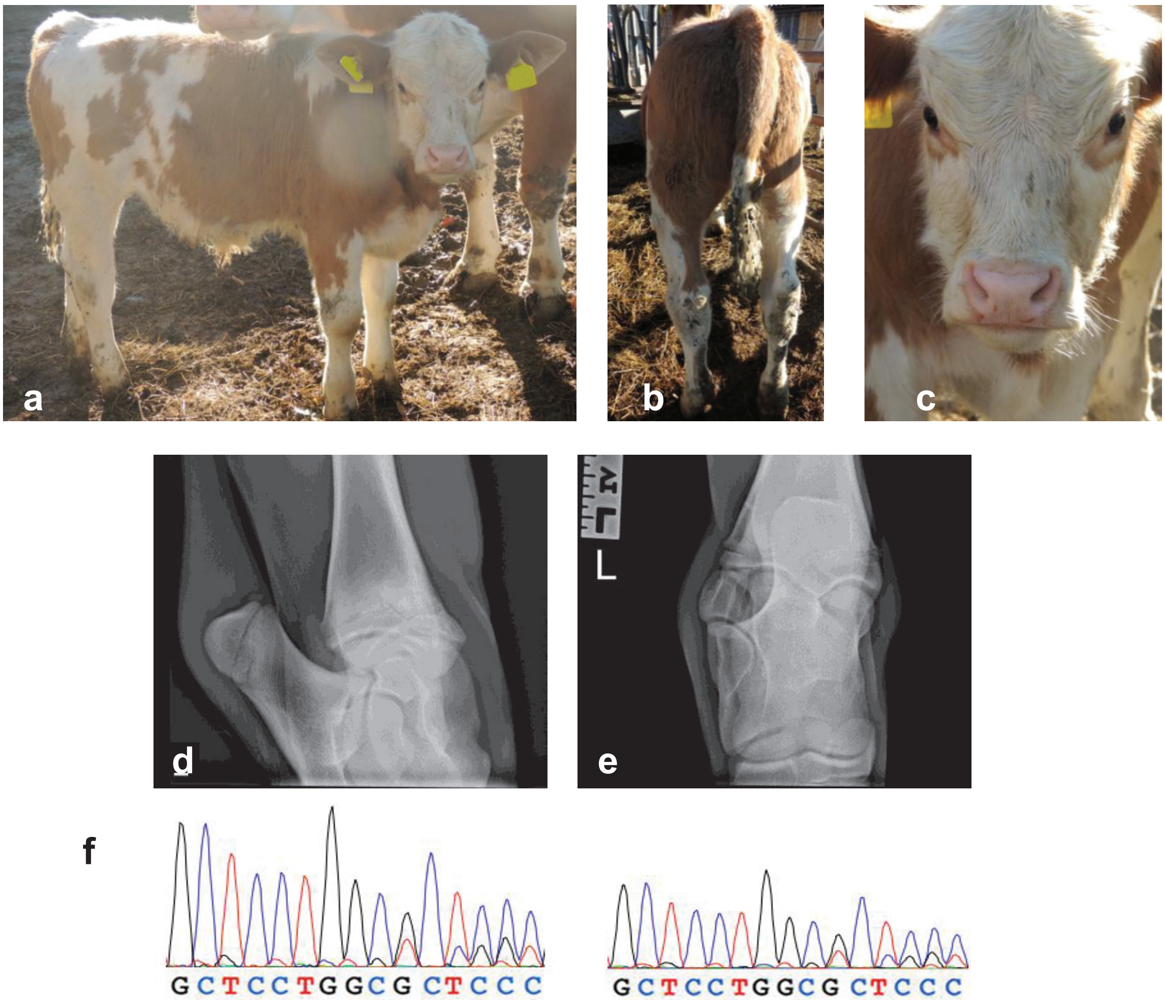

g

Sex	53K autosomal SNPs		1135 X-chromosomal SNPs	
	Call rate	Heterozygosity	Call rate	Heterozygosity
Male (N=4)	0.996	0.284	0.991	0.033
Female (N=8)	0.996	0.283	0.991	0.270
Chimera	0.856	0.309	0.915	0.183

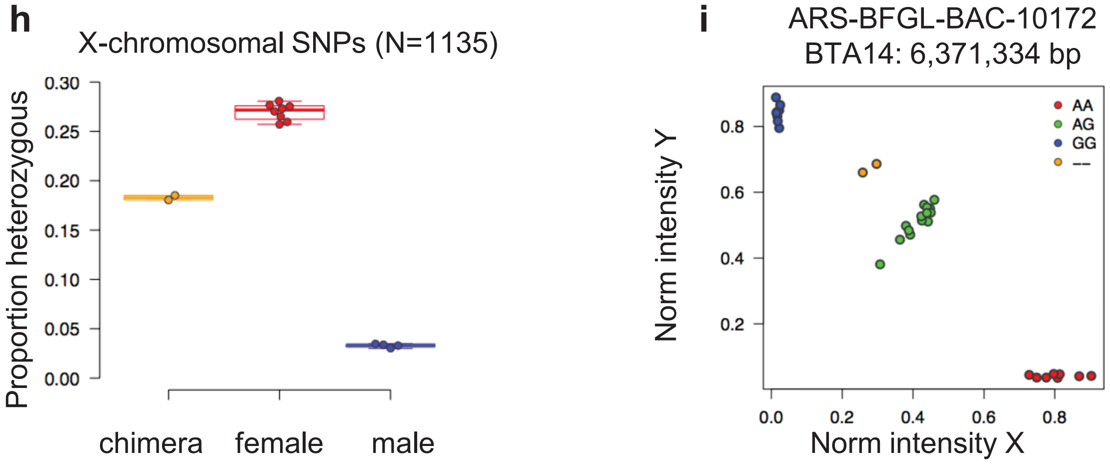

Supplementary Fig. 9: Genetic and phenotypic characterization of a chimera between wildtype and COL1A1 p. 1049_1050delinsS/+ heterozygous mutant cells.

Supplementary Fig. 9: Genetic and phenotypic characterization of a chimera between wildtype and COL1A1 p. 1049_1050delinsS/+ heterozygous mutant cells.
(**a-c**) Pictures of a 3-month old male Fleckvieh calf which was asymptomatic while carrying the g.37101299_37101302delinsT mutation on chromosome 19 responsible for the COL1A1 p. 1049_1050delinsS insertion-deletion. (**d,e**) Radiographs of the same calf showing no indication of microfracture or decreased bone density. Radiographs were kindly provided by the clinical unit of diagnostic imaging of the Veterinary University Vienna. (**f**) Electropherograms of the same calf showing superimposed sequences. Sanger sequencing was performed with DNA extracted from two blood samples that had been collected at two separate visits. In contrast with other heterozygous mutant animals (**Supplementary Fig. 2**), the intensity of the reference allele is approximately three-fold higher than the mutant allele suggesting either somatic reversion or blood cell chimaerism, although there was no evidence in pedigree records that the calf resulted from twin pregnancy. Both DNA samples were subsequently genotyped with the Illumina bovineSNP50 genotyping array to interrogates genotypes at 53K autosomal and 1135 X chromosomal SNPs (**g-i**). Compared to male and female relatives genotyped with the same array, the call rate of the calf was very low for both autosomal and X-chromosomal SNPs (**g**). Heterozygosity was clearly higher than in other male animals particularly for X-chromosomal SNPs (**h**). Analysis of the calf's raw genotyping data revealed that the signal intensity of SNPs with missing genotypes clustered in between homozygous and heterozygous genotypes (e.g. **i**). These results are compatible with blood cell chimaerism and suggest that the calf resulted from the fusion, at an early stage of gestation, between two male embryos showing different genotypes for the g.37101299_37101302delinsT variant on chromosome 19.

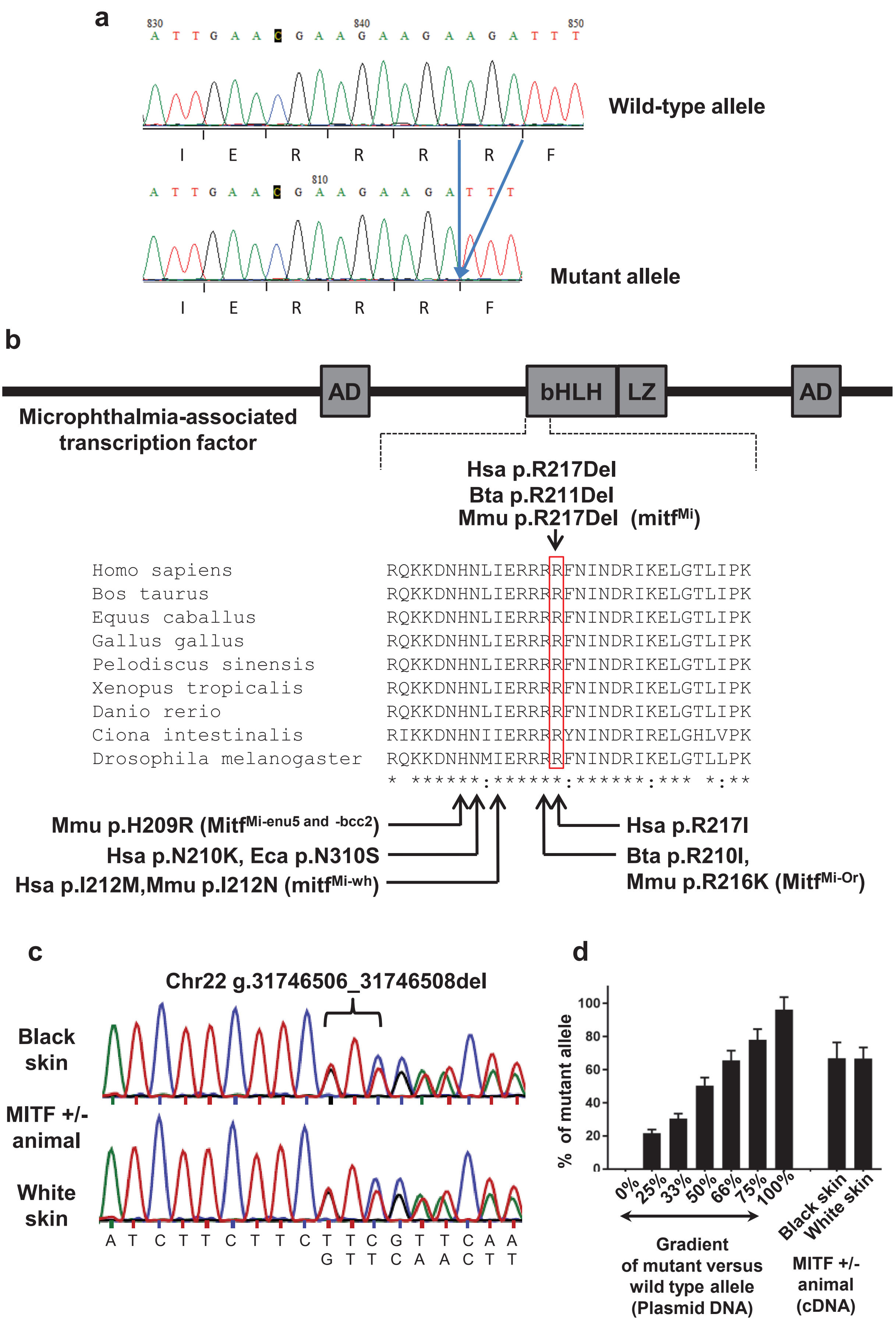

Supplementary Fig. 10: Molecular characterization of the candidate mutation for the Glass-Eyed Albino in Holstein cattle.

Supplementary Fig. 10: Molecular characterization of the candidate mutation for the Glass-Eyed Albino in Holstein cattle.

(a) Electropherogram of wild-type and mutant alleles. Sequences were obtained by cloning and sequencing MITF exons 6 to 8 in one heterozygous affected individual. The corresponding amino acid sequence is presented below, and the arginine deletion indicated by arrows. (**b**) Domain and region information for the MITF protein (AD: transactivation domain, bHLH : basic Helix-Loop-Helix, LZ : Leucine zipper). The DNA-binding domain is located in the basic part of the bHLH domain. Part of its sequence is outlined in the multispecies alignment of the MITF proteins from different species showing a complete conservation of the residue R217. Protein sequences accession numbers in Ensembl are ENSP00000295600, ENSBTAP00000008789, ENSECAP00000004487, ENSGALP00000042425, ENSMUSP00000044938, ENSXETP00000000313, ENSPSIP00000013650, FBpp0298327, ENSCINP00000009728 and ENSDARP00000056456. Arrows indicate the positions of mutations causing Tietz syndrome in humans (reviewed in Grill *et al.*[67]) and of mutations affecting the DNA-binding domain of MITF in non-human mammals[15-19]. (**c**) Electrophoregrams showing similar proportion of mutant and wild type allele in genomic DNA from black and white skin samples from the same MITF p.R211del+/- revertant animal. (**d**) Quantification of mutant allele expression in black and white skin samples from MITF p.R211del+/- revertant animals (n=3) using pyrosequencing. The y-axis represents the percentage of mutant allele in each sample. Results obtained with pools of plasmid DNA presenting different proportions of the mutant allele are presented for comparison. Note the slight overexpression of the mutant allele in both black and white skin samples from revertant animals.

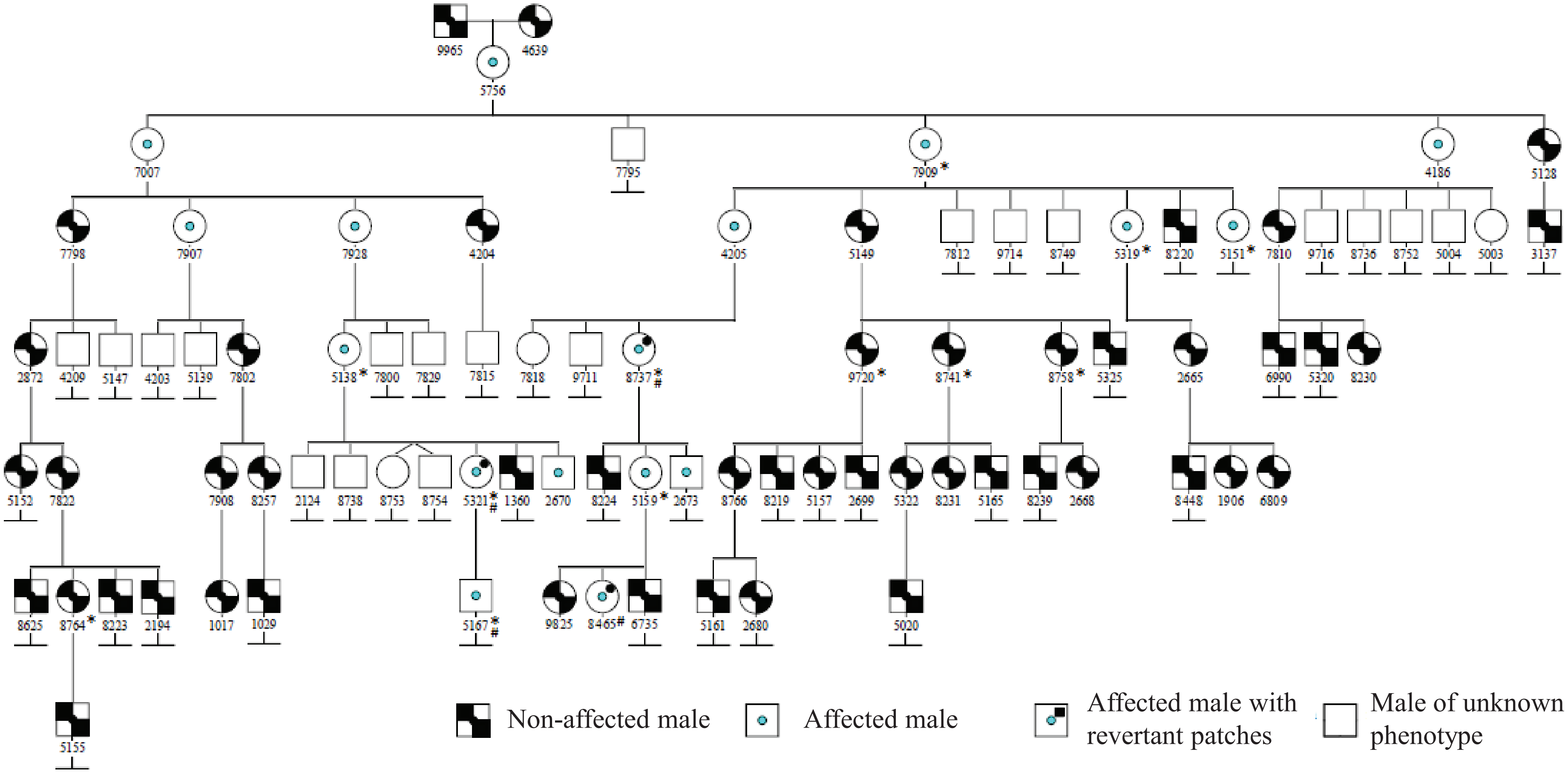

Supplementary Fig. 11: Pedigree of the GEA family
Except for the first mutant heifer, the sires which are all wild type Holstein artificial insemination bulls (based on picture information) have not been represented for reason of clarity. * Animals used for DNA analysis. # Animals used for gene expression analysis.
Note the relatively high proportion of males with unknown phenotype. This is essentially due to a lack of recollection regarding the phenotype of bull calves that were sold at three weeks of age several years before the beginning of the study.

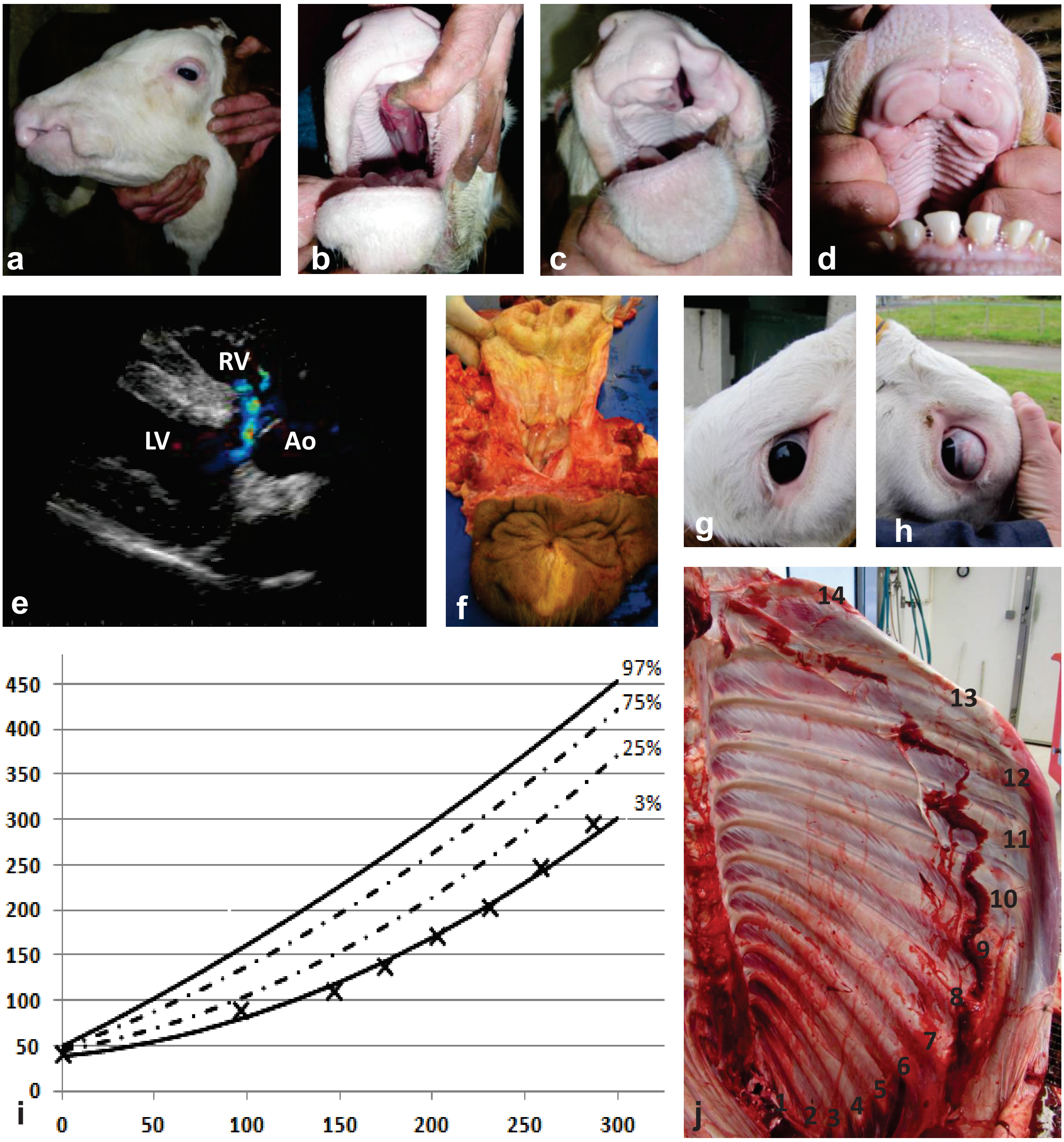

Supplementary Fig. 12: Additional clinical characterization our bovine model for CHARGE syndrome.

(**a**,**b**,**c**,**d**) Illustration of the variability of facial malformations observed in bovine and human affected individuals. Individual (d) presents two small bilateral holes in the palate. (**e**) Doppler echocardiography of a 2-year-old heifer affected by tetralogy of Fallot. LV: left ventricle; RV: right ventricle; Ao: aorta. (**f**) Dissection of the reproductive tract of a 18-month-old heifer possessing two cervix instead of one (and a unique uterus; not shown). (**g**; **h**) pictures of both eyes of a 18-month-old heifer after rapid lifting of the head. Note the abnormal position of the right eye in (g) which remains still as compared to the left eye (h). (**i**) Growth curve of the CHARGE sire Etsar in kg per day. For comparison the curves of the third, 25th, 75th and 97th percentile in a population of 467 young bulls raised in the same breeding center of Etsar and in the same conditions are displayed. (**j**) Detail of the skeleton of a 2-year-old heifer showing an additional pair of ribs (n=14).

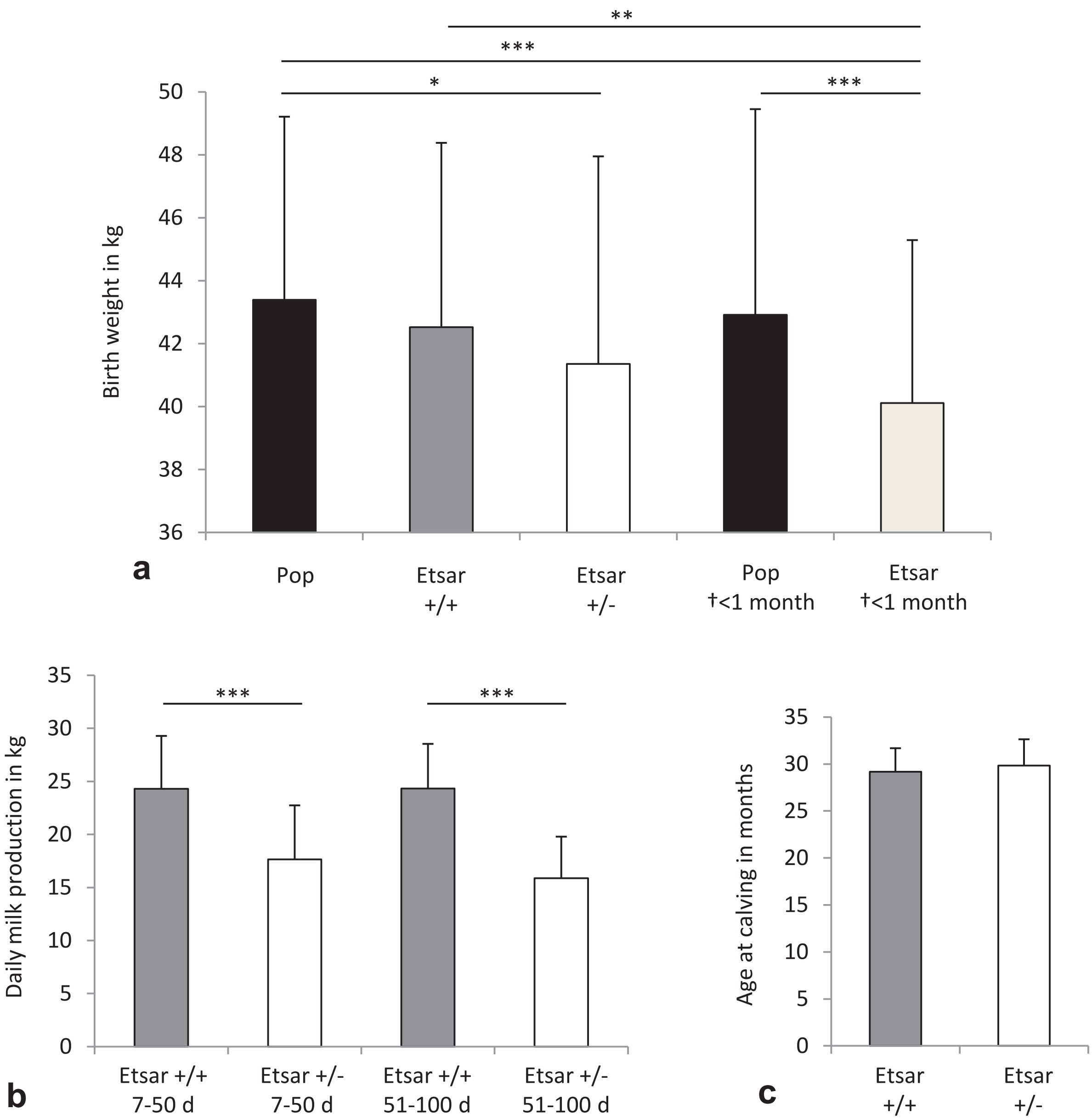

Supplementary Fig. 13. Birth weight for different groups of female calves among the Montbéliarde population and the descendants of Etsar.

a) Birth weight for herdmates of the descendants of Etsar (Pop; n=9375); homozygous wild type descendants of Etsar (Etsar +/+; n=106); descendants of Etsar that are heterozygous for the CHD7 frameshift mutation (Etsar +/-; n=39); herdmates who died before 1 month of age (Pop†<1 month; n=721) ; and descendant of Etsar who died before 1 month (Etsar†<1 month; n=56). **b**) Age at calving for homozygous wild type descendants of Etsar (Etsar +/+; n=61) and descendants of Etsar that are heterozygous for the CHD7 frameshift mutation (Etsar +/-; n=10). **c**) Daily milk production measured between seven and 50 days (7-50 d) and between 51 and 100 days (51-100 d) for homozygous wild type descendants of Etsar (Etsar +/+; n=58 and 55 respectively); and descendants of Etsar that are heterozygous for the CHD7 frameshift mutation (Etsar +/-; n=10 and n=7 respectively). *p<0.05, **p<0.01 and ***p<0.001 (T-test). Error bars represent standard deviation.

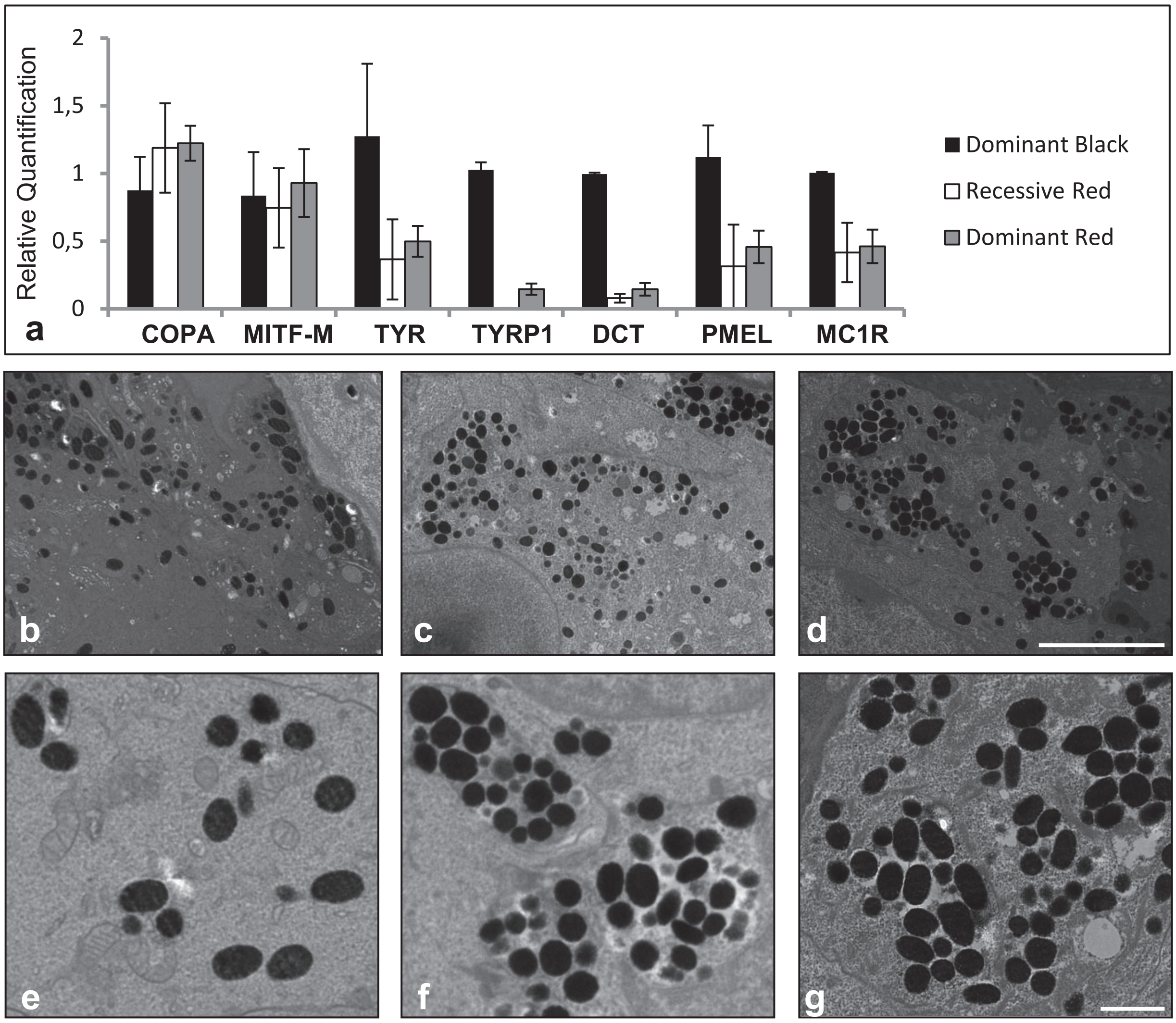

Supplementary Fig. 14: Analysis of gene expression in skin samples from dominant red and control animals and histological analysis under electron microscopy

(**a**) Relative expression quantification of genes involved in melanogenesis in skin biopsies from dominant black (MC1RD/D, DR+/+), recessive red (MC1Re/e, DR+/+), and dominant red (MC1RD/D, DRDR/+) Holstein animals. Each bar represents the mean relative quantification of the gene expression in the three groups analysed (n=2, 2 and 7, respectively). Error bars represent confidence intervals (95%). Larger confidence intervals are observed for dominant black and recessive red individuals due to the small number of samples analysed. However, the results obtained for these two groups are consistent with previous studies[68,69]. Expressions of MITF-M, the regulator of melanogenic genes transcription, and of COPA show similar patterns in the different groups. All the melanogenic genes, including MC1R, are downregulated in red animals compared to black ones. This downregulation is comparable between recessive and dominant red animals, although the mutation associated with the dominant red phenotype is not suspected to have an effect on gene regulation. (**b-g**) Transmission Electronic Microscopy pictures of hair bulb cells from dominant black (**b**,**e**), recessive red (**c**,**f**), and dominant red (**d**,**g**) Holstein animals. Note the absence of notable morphological differences between melanosomes from recessive and dominant red animals. A majority of them are spherical in contrast to the eumelanosomes from the dominant black animal which are more elliptical in shape. This observation is concordant with the presence of pheomelanosomes in recessive and dominant red samples (see **Supplementary Fig. 15**). Scale bars represent 5 µm in (**b-d**); and 1 µm in (**e-g**).

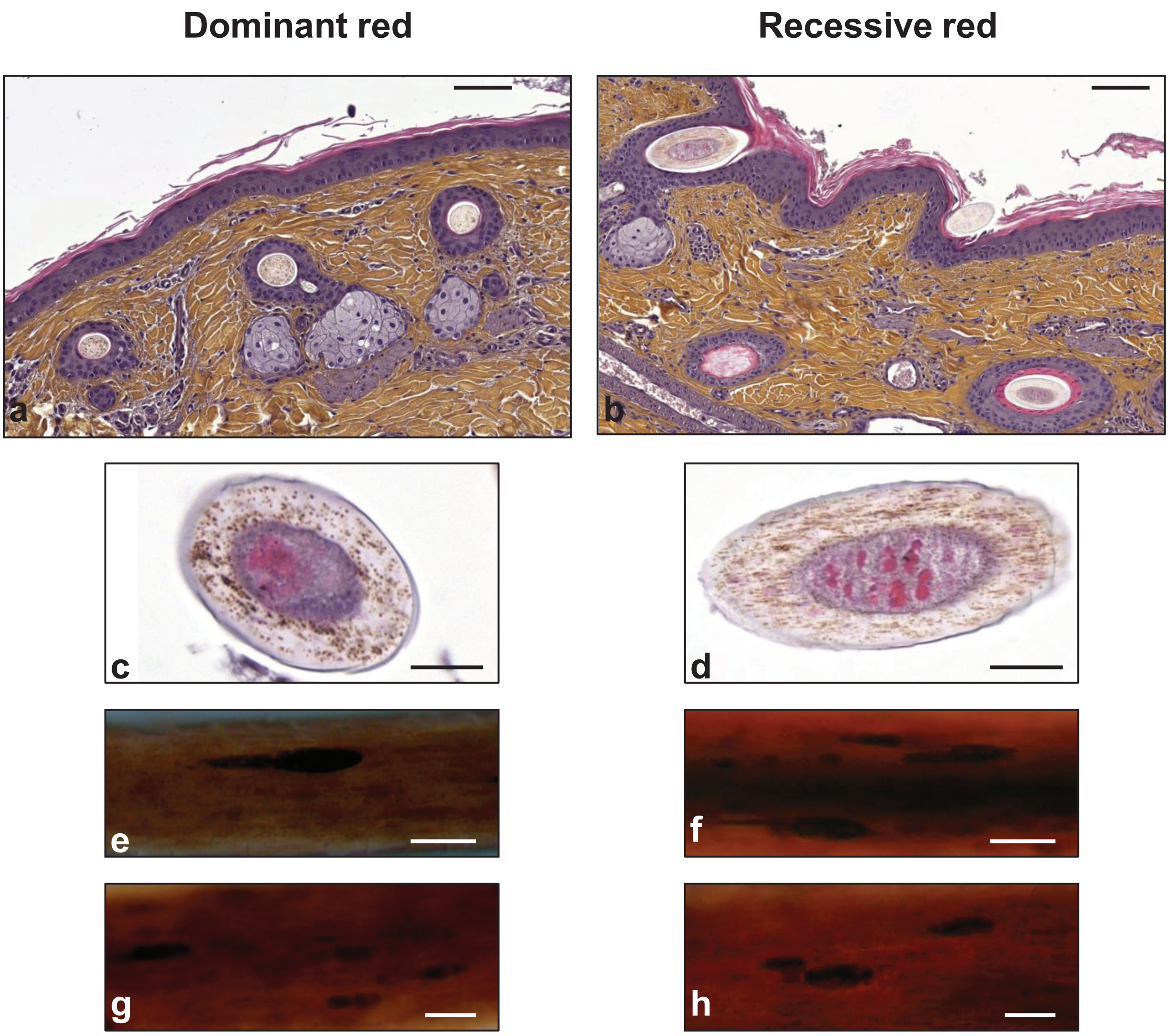

Supplementary Fig. 15: Histological analysis by optical microscopy of skin biopsies and hair samples from dominant and recessive red animals.
(**a-d**) HES staining of skin biopsies sampled in dominant red ($MC1R^{D/D}$ & $DR^{DR/+}$; **a**,**c**) and recessive red ($MC1R^{e/e}$ & $DR^{+/+}$; **b**,**d**) individuals. The overall skin structure (**a**,**b**) as well as a more detailed view on the hair sections (**c**,**d**) are comparable between the two phenotypes. (**e-h**) Hair samples of dominant red (**e**,**g**) and recessive red (**f**,**h**) individuals placed in a drop of lactophenol. The number and shape of pigment depositions in hair of both genotypes are similar. Scale bars correspond to 100µm (**a**,**b**) and 20µm (**c-h**).

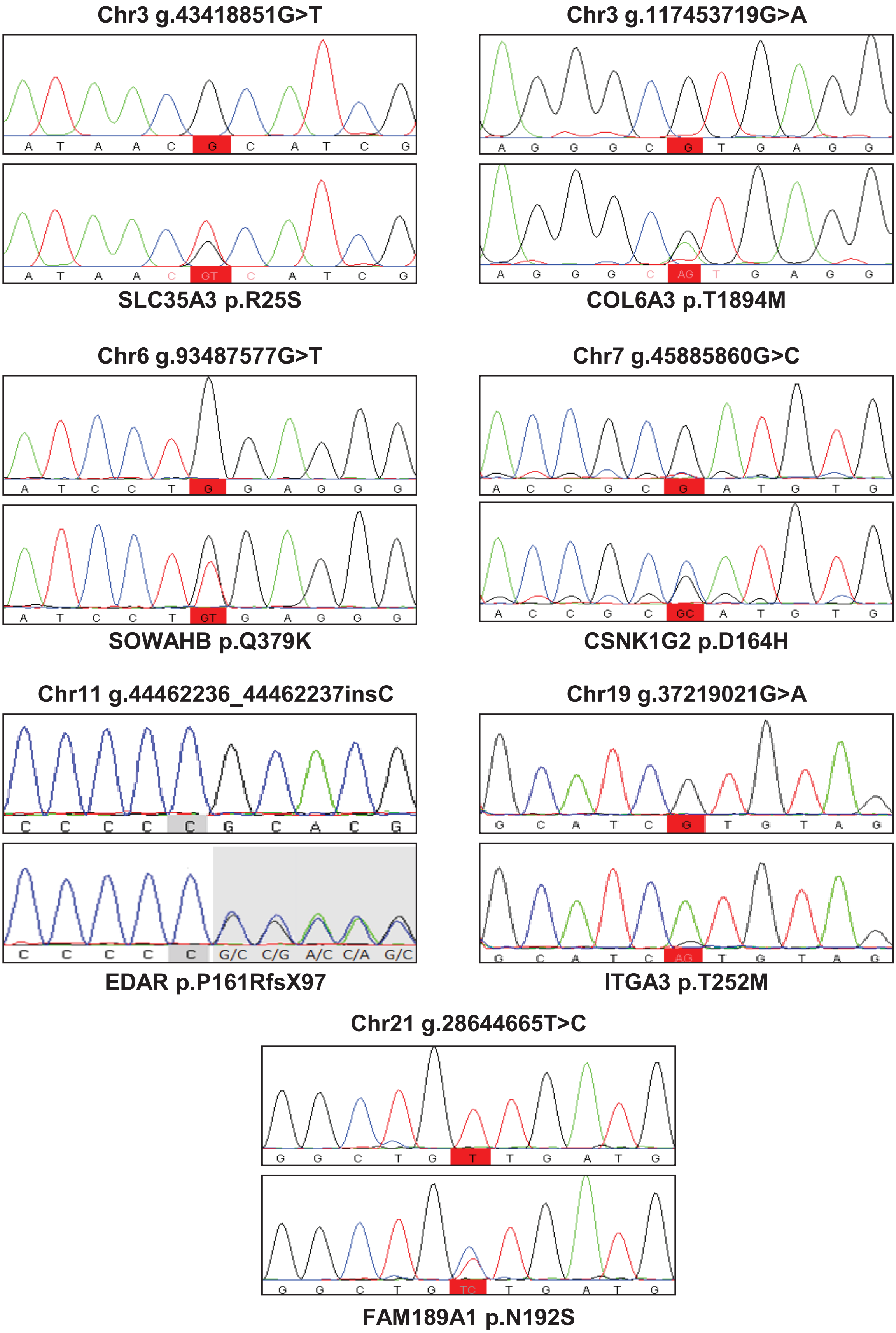

Supplementary Fig. 16: Electrophoregrams of homozygous wild type (up) and heterozygous (down) animals for seven private variants that have been shown to be *de novo* mutations.

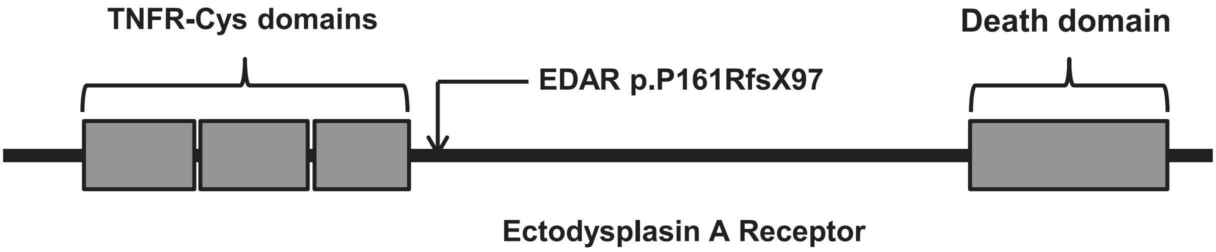

Supplementary Fig. 17: Domain information for the bovine Ectodysplasin A Receptor and localization of the EDAR p.P161RfsX97 mutation

Domain information for the bovine protein was deduced from information available for the human and mouse ortholog proteins in the UniProt database (http://www.uniprot.org/) . Accession numbers are E1BBS7, Q9UNE0 and Q9R187, for the bovine, human and mouse proteins, respectively.

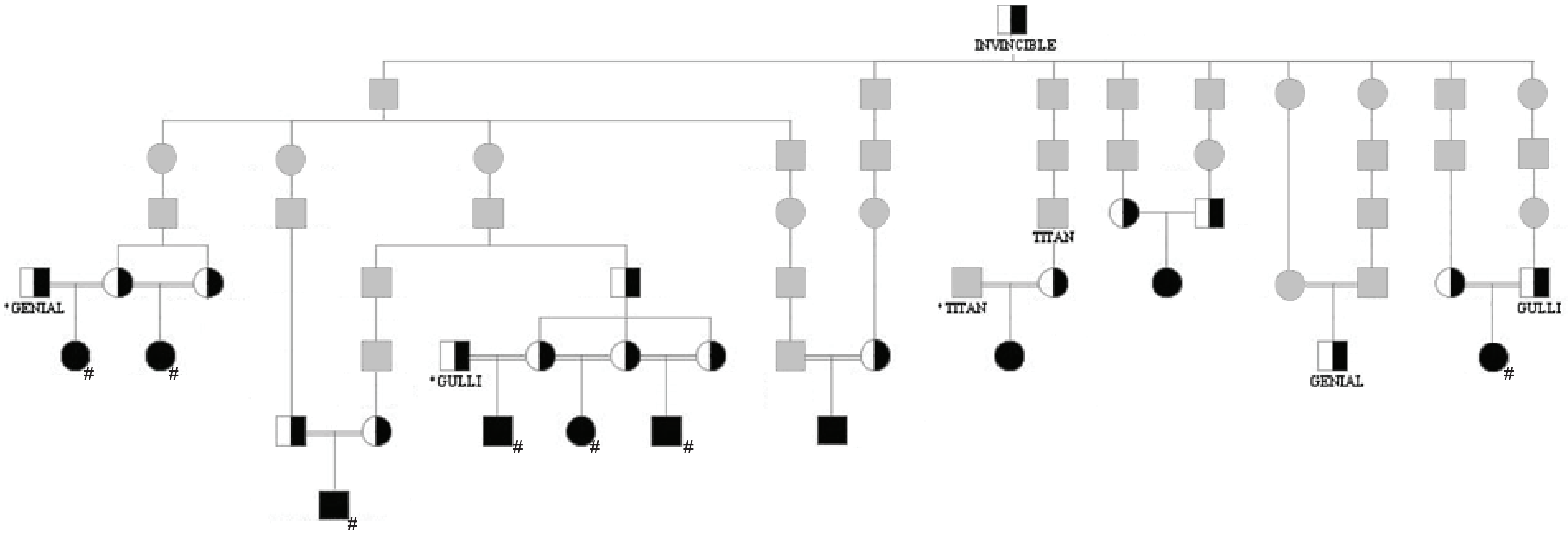

Supplementary Fig. 18: pedigree of the AED calves

Solid black squares and circles indicate respectively males and females affected calves which, for seven of them (#), have been confirmed to be homozygous for a frameshift mutation in *EDAR* and and a 26-Mb surrounding haplotype. Interestingly among the French genomic selection database we found three unaffected Charolais animals which (i) were also homozygous for the same 26-Mb IBD segment, (ii) descended from Invincible only one side of their pedigree and from its sire, which carries the same haplotype but without the *EDAR* frameshift mutation, on the other side, thus confirming the causality of the mutation. Black and white squares and circle indicate animals which have been genotyped as heterozygous carriers of the *EDAR* frameshift mutation. Grey squares and circle indicate individuals which were not available for genotyping.

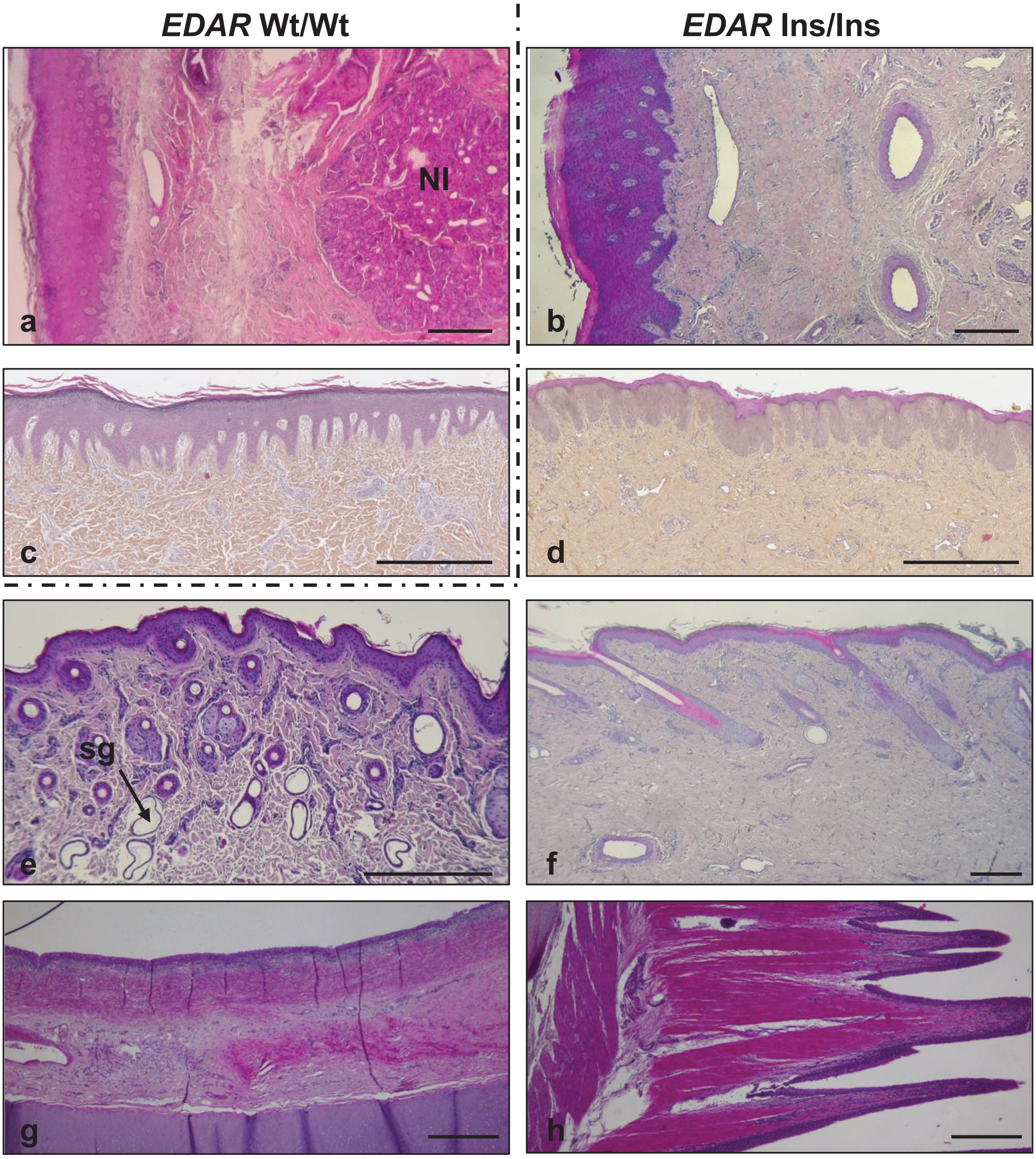

Supplementary Fig. 19: Additional histological analysis of skin biopsies from control and Anhidrotic Ectodermal Dysplasia-affected calves.

(**a**,**b**) Sections of muzzle showing a total absence of nasolabial glands (Nl) in a one-month-old AED calf (*EDAR* Ins/Ins, **b**) as compared with a matched control calf (Wt/Wt, **a**). These multilobular, tubuloalveolar, seromucoid glands, specialized apocrine sweat glands, are normally abundantly located in the deep portion of the muzzle. (**c**,**d**) Sections of horn bud from the same control (**c**) and AED (**d**) calves showing no notable morphological differences. (**e**) Section of eyelid from the same AED calf showing hypoplastic sweat glands (sg). (**f**) Section of skin from the fetlock. Whereas limb extremities present longer hairs in AED animals as compared with other regions of the body, skin biopsies reveal similar morphological defects (see **Fig. 6-j**). (**g**, **h**) Sections showing a total absence of mucous glands in tracheal (**g**) and bronchial mucosae (**h**). Scale bars represent 500 μM.

Supplementary Table 1: Details on some animals and designs used in the present study.

Analysis	Animals	Material/Methods/Data
**WGS approach for identifying the causative mutations for sporadic dominant syndromes**	One genome (for BD1, BD2, DR, GEA and NC) or one trio of genomes (for BD3) per condition as well as genomes of 1230 unaffected animals consisting of 345 Holstein, 220 Simmental, 140 Angus, 61 Jersey, 63 Brown Swiss, 34 Gelbvieh, 48 Charolais, 31 Hereford, 33 Limousin, 30 Guelph Composite, 29 Beef Booster, 28 Alberta Composite, 28 Montbeliarde, 25 Ayrshire Finnish, 24 Normande, 16 Swedish Red, 18 Danish Red, 16 Other Crosses, 10 Belgian Blue, 5 Angler, 5 Piedmontese, 4 Romagnola, 4 Salers, 2 Eringer, 2 Galloway, 2 Scottish Highland, 2 Unknown, 1 Blonde d'Aquitaine, 1 Cika, 1 Chianina, 1 Pezzata Rossa Italiana, 1 Tyrolean Grey	Whole Genome Sequencing data Illumina and SOLiD technologies<br><br>Screening for heterozygous deleterious mutations which are present in the genomes of the case group and which are absent from the genomes of their parents and from the control group
**WGS approach for identifying putative *de novo* mutations with recessive effects in the genome of healthy animals**	Forty-three French bulls (24 Holstein, 11 Montbéliarde, five Normande and three Charolais born from one to four generations) out of the 1230 control genomes above mentioned which were compared to the rest of the control genomes	
**Mapping of DR**	Thirty-one cases and 36 control relatives	Ilumina BovineSNP50 Beadchip phased data
**Mapping of GEA**	Eight cases, four control maternal cousins, their sires, and between 35 and 2340 unaffected paternal half sibs	
**Mapping of NC**	Etsar, 71 unaffected and 42 affected female progeny	Ilumina BovineSNP50 Beadchip phased data generated with FImpute from Ilumina BovineSNP50 or EuroG10K Beadchip genotyping data
**Mapping of modifier loci for NC**	Etsar, 89 unaffected, 49 mildly affected and 7 severely affected progeny	
**Confirmation of the de novo nature of candidate mutations for GEA, NC and DR**	Eight GEA animals and the sire of the first mutant which carries the ancestral version of the haplotype without the mutation; the NC bull Etsar and its parents; seven DR animals, and a black bull JOCKO BESNE (HOLFRAM005694028588) which presented the longest haplotype (interval Chr3:7,928,589-30,728,145) in common with a DR animal (HOLCHEM120093681213) and which was thus considered as a control carrying the same IBD haplotype but without the DR mutation.	Ilumina BovineSNP50 Beadchip phased data<br><br>PCR and Sanger sequencing
**Confirmation of the de novo nature of mutations detected in the genomes of healthy animals**	Three progeny of the sequenced sires, the sires, their own sires, maternal grand sires and great-grand sires	

Supplementary Table 2: List of deleterious private heterozygous polymorphisms identified in the genomes sequenced for each of the seven dominant conditions.

Defect	Chr.	Del. Priv. Het. Variant	Score	MQ	Summary of VEP annotations
GEA	3	g.20459207_ 20459208insAGG	50	60	ANP32E|ENSBTAG00000016730|ENSBTAT00000022237|inframe_insertion|p.D175delinsEG|
GEA	10	g.85657824G>A	136	60	PTGR2|ENSBTAG00000003747|ENSBTAT00000004878|missense_variant&splice_region_variant|p.R53H|deleterious(0)
**GEA**	**22**	**g.31746506_ 31746508del**	**42**	**58**	**MITF|ENSBTAG00000006679|ENSBTAT00000008789|inframe_deletion|p.R211del|**
**DR**	**3**	**g.9479761C>T**	**147**	**60**	**COPA|ENSBTAG00000004333|ENSBTAT00000005672|missense_variant|p.R160C|deleterious(0)**
DR	19	g.24911214G>A	40	60	TRPV1|ENSBTAG00000018880|ENSBTAT00000025131|missense_variant|p.R211W|deleterious(0)
DR	25	g.18641507_ 18641517del	93.5	45	ACSM3|ENSBTAG00000006447|ENSBTAT00000008455|frameshift_variant&feature_truncation|p.R367GfsX10|
NC	1	g.178569A>T	83	56	CLIC6|ENSBTAG00000001753|ENSBTAT00000002299|missense_variant|p.L382Q|deleterious(0.03)
NC	1	g.178741C>A	38	50	CLIC6|ENSBTAG00000001753|ENSBTAT00000002299|missense_variant|p.D325Y|deleterious(0.03)
NC	1	g.71028308_ 71028309insAAG	40.5	56	KIAA0226|ENSBTAG00000001890|ENSBTAT00000002461|inframe_insertion|p.E496_E497insK|
NC	1	g.72092618_ 72092635del	48.5	45	MFI2|ENSBTAG00000002998|ENSBTAT00000046533|splice_acceptor_variant&intron_variant||
NC	2	g.129402258T>C	69	60	MYOM3|ENSBTAG00000014885|ENSBTAT00000019817|missense_variant|p.L316P|deleterious(0)
NC	4	g.89008704_ 89008705insCAG	170	55	SPAM1|ENSBTAG00000004640|ENSBTAT00000006089|inframe_insertion|p.Q482_E482insQ|
NC	7	g.98596610del	42.5	60	ERAP1|ENSBTAG00000013557|ENSBTAT00000018026|frameshift_variant|p.G553AfsX16|
NC	8	g.25137158del	144	60	PLIN2|ENSBTAG00000005718|ENSBTAT00000047728|frameshift_variant&feature_truncation|p.V356CfsX26|
NC	10	g.38507021del	75.5	60	TMEM62|ENSBTAG00000013031|ENSBTAT00000017324|frameshift_variant|p.L620SfsX8|
NC	13	g.3815641del	116	58	SLX4IP|ENSBTAG00000034991|ENSBTAT00000049465|frameshift_variant&feature_elongation|p.M364NfsX4|
**NC**	**14**	**g.28085731_ 28085735del**	**213**	**58**	**CHD7|ENSBTAG00000021841|ENSBTAT00000026607|frameshift_variant|p.K594AfsX29|**
NC	14	g.4183085_ 4183086insACT	137	53	CHRAC1|ENSBTAG00000020226|ENSBTAT00000026936|stop_gained&inframe_insertion|p.E114X|
NC	22	g.58753732_58753733insGGG	110	60	CHCHD4|ENSBTAG00000015529|ENSBTAT00000020635|inframe_insertion|p.E113_E114insG|
NC	23	g.28503204G>A	70	47	BOLA|ENSBTAG00000002069|ENSBTAT00000031167|missense_variant|p.R340W|deleterious(0.01)
NC	29	g.49192201_ 49192202insCCCTCAGCG	30.5	29	Unknown transcript|ENSBTAG00000039129|ENSBTAT00000054963|inframe_insertion|p.I180_V181insALS|
OI*	2	g.124851135C>T	94	46	PTPRU|ENSBTAG00000012848|ENSBTAT00000017076|missense_variant|p.D819N|deleterious(0.05)
OI*	3	g.110776952G>T	103	48	TFAP2E|ENSBTAG00000001959|ENSBTAT00000002546|missense_variant|p.P37Q|deleterious(0.01)
OI*	6	g.7166112-7166125del	217	50	USP53|ENSBTAG00000010350|ENSBTAT00000049054|splice_acceptor_variant
OI*	6	g.108820884C>T	96	48	PDE6B|ENSBTAG00000017480|ENSBTAT00000052174|missense_variant|p.T172M|deleterious(0)

Defect	Chr.	Del. Priv. Het. Variant	Score	MQ	Summary of VEP annotations
OI*	10	g.5410636G>A	143	48	HRH2|ENSBTAG00000044071|ENSBTAT00000061359|missense_variant|p.A42V|deleterious(0.04)
OI*	11	g.75037927G>A	121	48	TP53I3|ENSBTAG00000003405|ENSBTAT00000004413|missense_variant|p.D232N|deleterious(0)
OI*	15	g.80485549G>A	130	47	|ENSBTAG00000035985|ENSBTAT00000020412|missense_variant|p.R299K|deleterious_low_confidence(0.01)
OI*	17	g.74529866C>T	85	49	SCARF2|ENSBTAG00000019375|ENSBTAT00000025815|missense_variant|p.P172L|deleterious(0.01)
OI*	18	g.48749390G>A	65	46	CAPN12|ENSBTAG00000013239|ENSBTAT00000044705|missense_variant|p.R526C|deleterious(0.01)
OI*	18	g.60459424C>A	224	47	|ENSBTAG00000030444|ENSBTAT00000017634|missense_variant|p.D488Y|deleterious(0)
**OI***	**19**	**g.37101299_37101302delinsT**	**118**	**50**	**COL1A1|ENSBTAG00000013103|ENSBTAT00000017420|protein_altering_variant|p.A1049_P1050delinsS**
OI*	23	g.32906331del	101	50	KIAA0319|ENSBTAG00000021903|ENSBTAT00000029204|frameshift_variant|p.E266RfsX3
BD1	3	g.14335856C>A	138	60	IQGAP3|ENSBTAG00000006882|ENSBTAT00000046733|stop_gained|p.S936X|
BD1	4	g.103372653C>T	102	60	KIAA1549|ENSBTAG00000021073|ENSBTAT00000028068|missense_variant|p.V1030M|deleterious(0)
BD1	4	g.40277950C>T	85	56	SEMA3C|ENSBTAG00000006138|ENSBTAT00000008076|missense_variant|p.S291L|deleterious(0)
BD1	4	g.89822822A>C	118	60	GPR37|ENSBTAG00000013732|ENSBTAT00000018241|missense_variant|p.F146C|deleterious(0.02)
BD1	5	g.111467981C>T	138	60	RPS19BP1|ENSBTAG00000017463|ENSBTAT00000023214|missense_variant|p.R101H|deleterious(0)
BD1	5	g.120924860C>T	83	53	ALG12|ENSBTAG00000046173|ENSBTAT00000064352|missense_variant|p.R406H|deleterious(0)
**BD1**	**5**	**g.32469820G>A**	**179**	**58**	**COL2A1|ENSBTAG00000013155|ENSBTAT00000017505|missense_variant|p.G600D|deleterious(0)**
BD1	5	g.35110549C>T	48	55	ANO6|ENSBTAG00000002902|ENSBTAT00000003770|missense_variant|p.G293S|deleterious(0.04)
BD1	7	g.20699614A>G	66	60	TNFAIP8L1|ENSBTAG00000037765|ENSBTAT00000055062|missense_variant|p.L170P|deleterious(0)
BD1	8	g.10863766C>A	107	35	ESCO2|ENSBTAG00000006551|ENSBTAT00000008606|missense_variant|p.D535Y|deleterious(0.03)
BD1	9	g.92870885C>T	106	60	SCAF8|ENSBTAG00000031917|ENSBTAT00000045257|missense_variant|p.T761M|deleterious(0.04)
BD1	10	g.28965990T>C	49	60	RYR3|ENSBTAG00000025642|ENSBTAT00000064357|missense_variant|p.Y1607C|deleterious(0)
BD1	11	g.103935960C>A	54	57	INPP5E|ENSBTAG00000001354|ENSBTAT00000001784|missense_variant|p.G104C|deleterious(0)
BD1	11	g.19824024C>T	92	60	QPCT|ENSBTAG00000013923|ENSBTAT00000018493|missense_variant|p.P82L|deleterious(0.02)
BD1	14	g.79340446G>A	42	60	LOC100196897|ENSBTAG00000046121|ENSBTAT00000063321|splice_donor_variant||

Defect	Chr.	Del. Priv. Het. Variant	Score	MQ	Summary of VEP annotations
BD1	15	g.32693584C>T	201	60	SORL1|ENSBTAG00000014611|ENSBTAT00000019457|missense_variant|p.R948W|deleterious(0.02)
BD1	16	g.56188164_ 56188165del	102	60	LOC615316|ENSBTAG00000012158|ENSBTAT00000055912|frameshift_variant&feature_elongation [illegible]
BD1	17	g.74715545T>C	69	60	C17H22orf39|ENSBTAG00000019894|ENSBTAT00000043931|missense_variant|p.E57G|deleterious(0.02)
BD1	18	g.25583688G>A	99	56	CCDC102A|ENSBTAG00000000462|ENSBTAT00000000588|missense_variant|p.R487W|deleterious(0)
BD1	18	g.57440224A>T	166	60	KLK9|ENSBTAG00000040177|ENSBTAT00000053012|missense_variant|p.C175S|deleterious(0)
BD1	18	g.62827815T>G	79	60	KIR2DL5A|ENSBTAG00000039215|ENSBTAT00000028845|splice_donor_variant||
BD1	18	g.62863037A>C	129	60	NCR1|ENSBTAG00000045529|ENSBTAT00000064403|missense_variant|p.K158T|deleterious(0)
BD1	23	g.23941623T>C	144	60	PKHD1|ENSBTAG00000011237|ENSBTAT00000032221|missense_variant|p.I2845V|deleterious(0.02)
BD1	23	g.6218736_ 6218739del	222	56	MLIP|ENSBTAG00000014581|ENSBTAT00000019407|frameshift_variant&splice_region_variant|p.I829KfsX31|
BD1	24	g.23515929_ 23515930del	47	56	NOL4|ENSBTAG00000010299|ENSBTAT00000013612|frameshift_variant&feature_truncation|p.Q449PfsX3|
BD1	25	g.1353746G>A	52	60	EME2|ENSBTAG00000016553|ENSBTAT00000022021|missense_variant|p.R295H|deleterious(0)
BD1	25	g.1592928G>A	148	60	NTHL1|ENSBTAG00000006272|ENSBTAT00000049780|missense_variant|p.A215V|deleterious(0)
BD1	25	g.2692336_ 2692337insACA[illegible]	159	41	MEFV|ENSBTAG00000019123|ENSBTAT00000025458|splice_acceptor_variant||
BD1	28	g.31320020_ 31320021insGGC	161	54	ZNF503|ENSBTAG00000014506|ENSBTAT00000019284|inframe_insertion|p.G20_G21insR|
BD2**	1	g.2781566G>A	17.1	47	HUNK|ENSBTAG00000020762|ENSBTAT00000027668|missense_variant|p.L608F|deleterious_low_confidence(0.0[illegible]
BD2**	2	g.4836164_ 4836165del	25.5	50	MYO7B|ENSBTAG00000039803|ENSBTAT00000061382|frameshift_variant|p.P1406LfsX56
BD2**	2	g.18394005T>C	20	49	PLEKHA3|ENSBTAG00000001098|ENSBTAT00000001453|missense_variant|p.I167V|deleterious(0.01)
BD2**	2	g.34607299C>T	23	49	SLC4A10|ENSBTAG00000015317|ENSBTAT00000020366|missense_variant|p.G921S|deleterious(0)
BD2**	3	g.42995273G>C	17.1	49	CDC14A|ENSBTAG00000031439|ENSBTAT00000065355|missense_variant|p.P194A|deleterious(0)
**BD2****	**5**	**g.32476082G>A**	**16.1**	**48**	**COL2A1|ENSBTAG00000013155|ENSBTAT00000017505|missense_variant|p.G996S**
BD2**	5	g.76106700C>T	17.1	49	CYTH4|ENSBTAG00000014237|ENSBTAT00000030432|missense_variant|p.T280I|deleterious(0)
BD2**	6	g.16814983G>A	46	48	CFI|ENSBTAG00000034501|ENSBTAT00000048867|missense_variant|p.G561E|deleterious(0)
BD2**	8	g.72455373G>A	26	48	ADAM28|ENSBTAG00000037929|ENSBTAT00000055272|missense_variant|p.G491S|deleterious(0)

Defect	Chr.	Del. Priv. Het. Variant	Score	MQ	Summary of VEP annotations
BD2**	8	g.78898280G>A	33	45	SLC28A3|ENSBTAG00000018280|ENSBTAT00000024328|stop_gained|p.Q242X
BD2**	11	g.93243947A>G	18.1	48	PTGS1|ENSBTAG00000006716|ENSBTAT00000008833|missense_variant|p.E492G|deleterious(0)
BD2**	14	g.56996294G>T	17.1	48	SYBU|ENSBTAG00000032548|ENSBTAT00000065047|missense_variant|p.D380Y|deleterious(0)
BD2**	15	g.29702674T>A	20	49	KMT2A|ENSBTAG00000018093|ENSBTAT00000024084|missense_variant|p.L3397M
BD2**	15	g.65527859C>T	17.1	50	NAT10|ENSBTAG00000016747|ENSBTAT00000022271|stop_gained|p.Q614X
BD2**	15	g.82925013C>G	17.1	48	OR5B17|ENSBTAG00000021233|ENSBTAT00000019371|missense_variant|p.L113F|deleterious_low_confidence(0)
BD2**	17	g.45536087C>T	17.1	48	POLE|ENSBTAG00000000590|ENSBTAT00000009882|missense_variant|p.P856S|deleterious(0.04)
BD2**	17	g.54514005G>A	20	49	SBNO1|ENSBTAG00000020505|ENSBTAT00000027325|missense_variant|p.A486T|deleterious(0.01)
BD2**	17	g.70827402C>T	26	44	NEFH|ENSBTAG00000013147|ENSBTAT00000029508|missense_variant|p.P809S
BD2**	17	g.71893085_71893093del	17.5	50	OSBP2|ENSBTAG00000019146|ENSBTAT00000025485|inframe_deletion|p.A839_G842del
BD2**	18	g.34998568-34998569del	35.5	50	SLC9A5|ENSBTAG00000039190|ENSBTAT00000054801|frameshift_variant|p.L138HfsX30
BD2**	18	g.44305960T>C	17.1	49	CHST8|ENSBTAG00000047670|ENSBTAT00000064164|missense_variant|p.L31P|deleterious(0)
BD2**	18	g.54080361G>A	22	48	PNMAL1|ENSBTAG00000009337|ENSBTAT00000012291|missense_variant|p.A66T|deleterious(0.02)
BD2**	19	g.45690566del	20.5	50	ARHGAP27|ENSBTAG00000000571|ENSBTAT00000010092|frameshift_variant|Q326GfsX8
BD2**	21	g.29654682del	16.6	49	PCSK6|ENSBTAG00000006675|ENSBTAT00000008785|frameshift_variant|p.P589LfsX27
BD2**	25	g.29393235A>G	17.1	48	WBSCR17|ENSBTAG00000008718|ENSBTAT00000029707|missense_variant|p.V571A|deleterious(0)
BD2**	27	g.16702723A>G	26	48	TRIML2|ENSBTAG00000008637|ENSBTAT00000011391|missense_variant|p.C420R|deleterious(0)
BD2**	29	g.41579474C>T	26	48	AHNAK|ENSBTAG00000013468|ENSBTAT00000052103|missense_variant|p.E4624K
BD2**	29	g.45509497T>A	20	49	PC|ENSBTAG00000019700|ENSBTAT00000026258|missense_variant|p.Y1013F|deleterious(0.05)
**BD3**	**5**	**g.32471813G>A**	**137**	**60**	**COL2A1|ENSBTAG00000013155|ENSBTAT00000017505|missense_variant|p.G720S|deleterious(0)**

Del. Priv. Het. Variant: deleterious private heterozygous variant. A total of 1172 genomes were used as control for filtering (see methods). *) The sequenced animal was the mosaic sire. **) To identify mutations compatible with BD2 syndrome in Holstein cattle, we applied a less stringent quality threshold (quality score = 15) compared to the other defects due to the lower genome coverage (9.0x) of the BD2 sequence data. Causative mutations are highlighted in bold.

Supplementary Table 3: Mapping of the DR, GEA and NC intervals

Defects	Animals	Mapping interval	Size of interval
DR	31cases and 36 control relatives	Chr3:7,906,099-12,475,989	4.6 Mb
GEA	8 cases and 4 control maternal cousins	Chr:22:31,446,714-33,076,318	1.6 Mb
NC	42 cases and 71 control half sibs	Chr14:27,916,840-28,625,324	0.7 Mb

See online methods for details on the mapping procedure. Note that the mapping of the DR locus has not needed to genotype any animal since BovineSNP50 Beadchip genotyping data from a large number of descendents of the mutant cow Surinam Sheik Rosabel-RED (HOLCAN000003541221) were available from the French Holstein genomic selection database (193,791 animals). Note also that for each of the animals studied, phasing was facilitated by the presence of their AI sires and of numerous of their half-sibs in the genomic selection databases.

Supplementary Table 4: List of private heterozygous polymorphisms identified in the genomes sequenced for the DR, GEA and NC conditions and located in the mapping intervals of the corresponding loci

Defect	Chr.	Private heterozygous variant	Score	MQ	Summary of VEP annotations
DR	3	**g.9479761C>T**	**147**	**60**	**COPA|ENSBTAG00000004333|ENSBTAT00000005672|missense_variant|p.R160C|deleterious(0)**
		g.10258823T>C	155	60	Intergenic_variant
		g.10397809_10397811del	209	57	Uncharacterized_protein|ENSBTAG00000019618|ENSBTAT00000054917|downstream_gene_variant
		g.11272722G>A	71	53	Olfactory_receptor|ENSBTAG00000038362|ENSBTAT00000056611|upstream_gene_variant
GEA	22	**g.31746506_31746508del**	**42**	**58**	**MITF|ENSBTAG00000006679|ENSBTAT00000008789|inframe_deletion|p.R211del|**
		g.32449448T>C	104	60	FRMD4B|ENSBTAG00000007624|ENSBTAT00000010028| intron_variant
		g.32500242T>A	109	60	FRMD4B|ENSBTAG00000007624|ENSBTAT00000010028|downstream_gene_variant
NC	14	**g.28085731_28085735del**	**213**	**58**	**CHD7|ENSBTAG00000021841|ENSBTAT00000026607|frameshift_variant|p.K594AfsX29|**
		g.28616159C>T	174	42	Intergenic_variant

Within the DR, GEA and NC mapping intervals (**Supplementary Table 3**), the average sequence coverage and the percentage of the UMD3.1 bovine sequence assembly that is not covered by sequence reads are 12.5 x and 2.8 %, 10.4 x and 0.2 %, and 12.2 x and 1.35 %, respectively. In addition to the detection of SNP and small indels across the whole genome performed with SAMtools pileup option[5], detection of structural variants has been undertaken in the DR, GEA, and NC mapping intervals using Pindel[7], DELLY[8].and IGV[9]. As a consequence this table presents the exhaustive list of private heterozygous variants located in these intervals on the UMD3.1 version of the bovine genome assembly.

Supplementary Table 5: Results of the verification of the *de novo* nature of the private heterozygous variants located in the DR, GEA and NC mapping intervals.

<table>
<tr><th>Defect</th><th>Private heterozygous variant</th><th>Confirmed as de novo ?</th><th>Comment</th></tr>
<tr><td rowspan="4">DR</td><td>Chr3. g.9479761C>T
COPA p.R160C</td><td>Yes</td><td>Associated with the DR haplotype and absent from a 22.8-Mb IBD haplotype (Chr3:7,928,589-30,728,145) encompassing the DR locus which also segregates in the Holstein population without the DR phenotype</td></tr>
<tr><td>Chr3. g.10258823T>C</td><td rowspan="3">No</td><td rowspan="3">Not associated with the DR haplotype</td></tr>
<tr><td>Chr3. g.10397809_ 10397811del</td></tr>
<tr><td>Chr3. g.11272722G>A</td></tr>
<tr><td rowspan="3">GEA</td><td>Chr22 g.31746506_ 31746508del
MITF p.R211del</td><td>Yes</td><td>Associated with the GEA haplotype and absent from the unaffected sire of the first mutant heifer which possesses the same haplotype without the mutation</td></tr>
<tr><td>Chr22 g.32449448T>C</td><td rowspan="2">No</td><td rowspan="2">Associated with the GEA haplotype but also present in the unaffected sire of the first mutant heifer which possesses the same haplotype without the mutation</td></tr>
<tr><td>Chr22 g.32500242T>A</td></tr>
<tr><td rowspan="2">NC</td><td>Chr14 g.28085731_ 28085735del
CHD7 p.K594AfsX29</td><td>Yes</td><td>Associated with the NC haplotype and absent from the unaffected dam of the NC sire which possesses the same haplotype without the mutation</td></tr>
<tr><td>Chr14 g.28616159C>T</td><td>No</td><td>Not associated with the NC haplotype</td></tr>
</table>

Each of these candidate variants were genotyped by PCR and Sanger sequencing on a panel of cases and when necessary on a panel of ancestors or related controls carrying the same IBD haplotype but without the mutation (see online methods and Supplementary Table 1 for details on animals).

Supplementary Table 6: Evaluation of the NC (i.e. CHARGE) sire for four semen production traits

	Volume (mL)	Concentration (millions/mL)	Sperm motility after thawing (score)	Percentage of live sperm after thawing
CHARGE sire	1.8 ± 0.2 ***	933.6 ± 112.8 *	3.3 ± 0.4 ***	62.1 ± 8.9 **
78 control sires	3.5 ± 0.7	1007.0 ± 288.2	2.8 ± 0.6	53.5 ± 14.8
Rank of the CHARGE sire	79/79	47/79	8/79	16/79

Values expressed as means ± standard deviation. The four traits were recorded in 12 different semen collection sessions for the CHARGE sire and in 1077 sessions for the control group. *$p<0.05$, **$p<0.01$ and ***$p<0.001$ versus control group (Student t-test). Sperm motility score range from 0 (bad) to 5 (excellent). A rank of 79/79 corresponds to the worst average performance among the average performances of each individual bull.

Supplementary Table 7: Mortality rate among the progeny of the bovine CHARGE bull Etsar and in the Montbéliarde breed.

Mortality (in %)	Sex	1st month	2nd month	3rd month	4th month	5th month	6th month	Summ
Progeny of Etsar (real data)	♂	27.1	2.3	2.7	0.8	1.6	0.4	34.9
	♀	21.0	4.3	2.6	2.6	0.7	1.0	32.2
Breed reference values	♂	13.7	1.7	1.1	0.9	0.8	0.6	18.8
	♀	9.5	1.2	0.7	0.6	0.4	0.3	12.7
Progeny of Etsar (estimated per genotype)	+/- ♂	40.5	2.9	4.3	0.7	2.4	0.2	51.0
	+/- ♀	32.5	7.4	4.5	4.6	1.0	1.7	51.7

To estimate the Mortality of the heterozygous carriers of the *CHD7* frameshift mutation we used the following equation: Mortality of Estar's progeny = 0.5*Mortality of carriers of the mutation + 0.5 Mortality of non carriers which simplifies in: Mortality of carriers of the mutation =2*Mortality of Etsar's progeny – Mortality of non carriers. We assumed that the mortality of non carriers was equal to the breed reference values.

Supplementary Table 8: Results of gene set enrichment analyses on Top canonical pathways using Ingenuity Pathway Analysis.

Analysis	Ingenuity Canonical Pathways	p-value	Genes
FC≥2	Unfolded protein response	4.3x10-5	ERN1,HSPH1,HSPA1A/ HSPA1B,HSPA6
	Protein Ubiquitination Pathway	3.2x10-4	USP31,HSPH1,HSPA1A/HSPA1B ,HSPA6,USP37,HSPA4L
	Aldosterone Signaling in Epithelial Cells	3.3x10-4	PIK3R3,HSPH1,HSPA1A/ HSPA1B,HSPA6,HSPA4L
	eNOS Signaling	2.4x10-3	PIK3R3,ADCY1,HSPA1A/ HSPA1B,HSPA6
	Taurine Biosynthesis	7.2x10-3	CDO1
FC≤0.5	Bile Acid Biosynthesis, Neutral Pathway	5.1x10-4	CYP3A4,CYP8B1
	Inhibition of Angiogenesis by TSP1	3.5x10-3	TGFB1,MMP9
	Atherosclerosis Signaling	4.5x10-3	ALOX15B,TGFB1,MMP9
	Hepatic Cholestasis	8.3x10-3	TGFB1,IL25,CYP8B1
	Role of Cytokines in Mediating Communication between Immune Cells	8.7x10-3	TGFB1,IL25

Results are presented for two different analyses using the list of genes that are significantly upregulated (FDR<0.05, fold change >2) and downregulated (FDR<0.05, fold change <0.5) in skin samples from dominant red versus dominant black animals (Supplementary Data 1) according to the RNA sequencing data produced by Dorshorst *et al.* [41]. The p-value is adjusted for multiple testing. Canonical pathways with adjusted p-value >0.01 are not presented.

Supplementary Table 9: Results of gene set enrichment analyses on MGI Mammalian Phenotype Level 3 and 4 using EnrichR.

Analysis	Name	Adjusted p-value	Genes
FDR<0.05 FC>0, MGI MP level 3	MP0010769 Abnormal survival	2.0x10-2	FOXA, PGAP1, BMPR2, LDB1, SETD8, MAML1, IRS1, AVIL, RORA, PRDM2, IRS2, AFF1, SYNE1, GLS, ICAM1, AMOT, COL4A3BP, SERP1, HERC2, SPTLC2, CAPN7, AP1G1, CREB3L2, EP300, CASP2, SOX6, CDON, DDX17, RBM15, APAF1, NCOA6, NCOA3, SLC35C1, SLC30A4, ZFR, DYRK1A, TET2, ARID5B, LMTK2, TANC2, TGFBR3, ERN1, ATRN, EHHADH, DNAJC5, NUMB, COL4A5, IPPK, SLC26A2, PRKDC, MYCBP2, PLA2G3, PPM1K, MST1R, ACVR1B, ATXN7, PLAGL1, ADRBK1, RDH10, HMOX1, PLAGL2, STBXBP5, SOCS7, SPEN, MAP3K3, NFYA, HIPK1, KLF3, PBX1, PTPRB, RYBP, ERCC4, ATG16L1, NEDD4, DLC1, ASXL2, ABI1, EIF2C2, FGFR3
FC>0, MGI MP level 4	MP0005621 Abnormal cell physiology	6.8x10-3	SLC26A2,SETD8,NCOA6,IRS1,NCOA3,ARID5B,IRS2,ACVR1B,KLF3,ICAM1,ERN1,COL4A3BP,CLCN5,ATG16L1,DLC1,EP300,SLC12A6,HSPA1A
	MP0005384 Cellular phenotype	6.8x10-3	SLC26A2,SETD8,NCOA6,IRS1,PRKDC
	MP0005076 Abnormal cell differentiation	1.6x10-2	IRS1,NCOA3,IRS2,ACVR1B,KLF3,NCOA3,ARID5B,IRS2,ACVR1B,KLF3,ICAM1,ERN1,COL4A3BP,CLCN5,ATG16L1,ERCC4,PLAGL1,DLC1,EHHADH,EP300,SLC12A6,HSPA1A
FC<0, MGI MP level 3	MP0010678 Abnormal skin adnexa	3.6x10-2	KRT71,DLX3,TGFB1,LEF1,ARID4A,OXT,BARX2,CST6,ASS1,RELB,GJB2,GJA1,IFT88,KRT17,LHX2,KRT25,SGK3,SLC39A2,CTSC
	MP0010680 Abnormal skin adnexa	4.3x10-2	GJA1,DLX3,KRT17,LHX2,SGK3,OXT,BARX2
	MP0002138 Abnormal hepatobiliary system	4.3x10-2	OCA2,TGFB1,RDX,GATA6,ADK,ARID4A,LSR,PTN,CXCL4,RELB,GNMT,IFT88,RAD50,MYCN,LHX2,CD49,PDCD1,SLC27A5
	MP0002060 Abnormal skin morphology	4.3x10-2	KRT71,HSD3B7,DLX3,LEF1,CST6,LSR,MMP9,ASS1,RELB,LMF1,NFKBIA,GJB2,GJA1,LHX2,PDPN,SGK3,CACNA15,SLC39A2
	MP0002133 Abnormal respiratory system	4.3x10-2	IL33,EGR2,TGFB1,IL25,ECEL1,LTBP4,ADK,ARID4A,MMP9,RELB,CTGF,LMF1,RAD50,GJA1,MYCN,NFIB,PDPN,PTGDR
FC<0, MGI MP level 4	None	-	None

Results are presented for different analyses using the list of genes that are significantly upregulated (FDR<0, fold change >1) and downregulated (FDR<0.05, fold change <1) in skin samples from dominant red versus dominant black animals (**Supplementary Data 1**) according to the RNA sequencing data produced by Dorshorst *et al.* [41]. The p-value is adjusted for multiple testing. Canonical pathways with adjusted p-value >0.05 are not presented.

Supplementary Table 10: Results of the verification of the *de novo* nature of the heterozygous private variants located in the genome of 43 AI sires.

Bull's ID	YoB	Mutation	Confirmed as de novo ?	Comment
NORFRAM 00729XXX	1997	Chr3 g.67614411A>C AK5 p.L10R	No	Carried by the bull's sire
MONFRAM 00259XXX	1998	Chr16 g.553427C>G PPFIA4 p.R65G		
MONFRAM 00712XXX	2000	Chr3 g.106051353G>A NFYC p.P256S		Carried by the maternal grand dam's sire of the bull
NORFRAM 00509XXX	1997	Chr25 g.30046676_30046677insCGG AUTS2 p.A701_D702insA		Carried by the dam's sire and maternal grand dam's sire of the bull
HOLFRAM 00214XXX	2006	Chr2 g.125113169G>A EPB41 p.P15L		Absent in the bull and its progeny according to Sanger sequencing
HOLFRAM 00329XXX	1997	Chr18 g.35737927G>A ESRP2 p.R252C		
NORFRAM 00724XXX	1999	Chr25 g.41186609_41186610insACAG AMZ1 (splice acceptor variant)		
NORFRAM 00539XXX	1997	Chr26 g.18519611G>A PGAM1 p.G234R		
NORFRAM 00729XXX	1997	Chr1 g.154103401C>T SH3BP5 p.D39N	Unknown	No carrier of the haplotype associated with the mutant allele among sire, dam's sire and maternal grand dam's sire of the bull.
NORFRAM 00539XXX	1997	Chr10 g.57907600G>A FAM214A p.M977I		
MONFRAM 00380XXX	1999	Chr19 g.21774636C>T EFCAB5 p.R1135X		
MONFRAM 00712XXX	2000	Chr3 g.43418851G>T SLC35A3 p.R25S	Yes	Occurred de novo on the paternal chromosome
CHAFRAM 00650XXX	2000	Chr6 g.93487577G>T SOWAHB p.Q379K		
CHAFRAM 001893105503	1993	Chr11 g.44462236_44462237insC EDAR p.P161RfsX97		
MONFRAM 00254XXX	1999	Chr21 g.28644665T>C FAM189A1 p.N192S		
HOLFRAM 00443XXX	2007	Chr3 g.117453719G>A COL6A3 p.T1894M		Found on the maternal chromosome. Maternal grand sire is carrier of the associated haplotype but not of the mutation
HOLFRAM 00589XXX	1996	Chr19 g.37219021G>A ITGA3 p.T252M		
MONFRAM 00252XXX	2000	Chr7 g.45885860G>C CSNK1G2 p.D164H		Found on the maternal chromosome. Maternal grand dam's sire is carrier of the associated haplotype but not of the mutation

Yob: Year of birth. Note that except for Invincible (CHAFRAM001893105503), the international ID of the bulls have been partially anonymized upon the request of the breeding companies. For each mutation, a panel of seven animals with phased Illumina bovine SNP50 genotyping data available and consisting in three progeny of the sequenced sire, the sire, its own sire, maternal grand sire and grand dam's sire was genotyped by PCR and Sanger sequencing (see methods). Among the 18 private deleterious mutations, only seven were confirmed to have occurred *de novo*. Except for *FAM189A1* and *CSNK1G2* for which, to our knowledge, no homozygous mutant has been reported in the literature, the five other mutations affecting *COL6A3*, *EDAR*, *ITGA3*, *SLC35A3* and *SOWAHB* are expected to cause severe recessive conditions. Homozygous mutations in these genes can cause Bethlem myopathy 1 (MIM: 158810), dystonia 27 (MIM: 616411) or Ullrich congenital muscular dystrophy 1 (MIM: 254090) in humans for *COL6A3*; anhidrotic ectodermal dysplasia in humans (MIM: 224900) for *EDAR*; interstitial lung disease, nephrotic syndrome, and epidermolysis bullosa (MIM: 614748) in humans for *ITGA3*; complex vertebral malformation in cattle[70] for *SLC35A3*; and exencephaly and abnormal neural tube in mouse[71] (http://www.informatics.jax.org/marker/MGI:1925338) for *SOWAHB*.

Supplementary Table 11: Details on primers used in this study.

Gene or variant	Forward/Reverse primers	Purpose
B2-microglobulin	AGACACCCACCAGAAGATGG/TCCCCATTCTTCAGCAAATC	RT-qPCR
COPA	ACCCTACTATGCCACTCATT/ TATCAACTTCCCATGCTTTT	
DCT	ACTCTTTTTAACCGCAGACCAACT/TGAGAGCACTGTGGTCCAATCT	
GAPDH	CCAACGTGTCTGTTGTGGATCTGA/GAGCTTGACAAAGTGGTCGTTGAG	
MC1R	CAGCCTGCTCTTCATCACCT/AGCATGTGGACGTAGAGGAC	
MITF-M	TCACTATCAGGTGCAGACCCAC/CAGGACTTGGTTGGCATGTTTA	
PMEL	GGATTGTGTTCTGTATCGCTATG/CACTCTCAATACCCTGGACAATGT	
RPL32	CTGCTGATGTGCAACAAATCTTACT/ATGGCCTTGCGGTTCTTG	
TYR	CCTACAAGATTCAGAACCGGACAT/GATTCGTTGCGCTTGTTCTAAGT	
TYRP1	GAAATGTTTGTTACTGCTCCAGACA/CTCCGACTTGGCCATTGAA	
CHD7 p.K594AfsX29	GTGCCGGATATGACTCAGGT/TACTATTTGGTGGCGGGTGT	Genotyping of candidate mutations for the dominant syndromes
COL1A1 p.A1049_P1050delinsS	TCCTTGGCTGATGTTCACCT/GGCCAAGCAAAGAGAATGGT	
COL2A1 p.G600D	GATGAGTCCCGTGTGTGATG/GGATCAGCGCCTCTCTTATG	
COL2A1 p.G720S	CTGTGAATCTGCAGCGTGTT/TGAGATGGAGGGATGTGTCA	
COL2A1 p.G996S	GAAGGGAGAGCCTGGAGATG/CGACAAGGAGGGTGAGTGT	
COPA p.R160C	CGGCCTCTGATTTGGTGTTG/AAGCCCTGTTTCCCTGTACT	
MITF p.R211del	TTCCCACCTCCAAAGCTGAA/TGGAGGATCAGGGTCGAGTA	
Chr3 g.10258823T>C	TTGCTCCTTTCTGTCTGCTG/ATGTGGTGGTCTCTCTCTTGA	Genotyping of other private heterozygous mutation located in GEA, DR and NC mapping intervals
Chr3 g.10397809_ 10397811del	CCAAAAAGGGATATCACCAA/CCCATATCATCACTCAGATGTCA	
Chr3 g.11272722G>A	GGCCTCATCCAAGGAGCTAT/TGAATGTCTTTTTCCTAGCCGTA	
Chr22 g.32449448T>C	CAGGAAGCGGTAGGGAAGTA/CACTGGGCTGTGTCAAATGT	
Chr22 g.32500242T>A	AGAGTGGCATTATGGCGTTT/TTCTCAGCACGTGTGTCTCC	
Chr22 g. 28616159C>T	ATCAACTGCCCCTCATTCAC/GATCGGCAAAAATGTGGAGT	
AK5	AGGCTAGCAAGCTTCTGCAA/GAGCGCTGAGACCACAGTC	Genotyping of putative de novo deleterious recessive mutations
AMZ1	CCAGGTGTCCCCATGAAG/GAGCTCTGACCGTTCCTCAC	
AUTS2	ATCATGCGAGTCCTGTCCA/AGATCAGGGGGCATTTGAG	
COL6A3	CCCCACCAAACATGAAGAAC/GCTTGCCTGTACGTTGAAA	
CSNK1G2	AGGACCTGTTCGACTTGTGC/TGCGTGTTGATGCTCATGTA	
EDAR	TCTTGGACTGAGCAGGTGTG/GGACTCTGAAGGTGCCTGAG	
EFCAB5	GGCATCATTGTGGCTCAAA/AGAAGCAGAAGCTGGCAGTC	
EPB41	TCTTTGCCTTCCTCCTCAGA/TGATTGAGGGTTTTGCCTTC	
ESRP2	TTGAGGCACAGTGCTACACC/TCCTTTTTGCAGAGCTTGGT	
FAM189A1	CAGACAGGGCTGAGACATCA/ACCAGTATCCTTTGAGAGCAGT	
FAM214A	TCCTTCAAACTGAATTATATCCACTG/TTCCAAGGGGCACTTGTCTA	
ITGA3	CCCTGGATTCTTACCCATCA/CCCAGAGGCAGAGTCAAAAC	
NFYC	TCGGATAAAGCCCATCTGTC/TTTCCGAGTTGGCTTCTTTG	
PGAM1	AAGCTGGAGAGGTCTGTGGA/GGGAGTGGGTGCAAGAGATA	
PPFIA4	TGTGAAGTGATGCCCACAAT/TTCTCCAGGTGAAGCCAGTT	
SH3BP5	CCCTTTCCTCTTTGTTGCAC/CGCTCGGAGCTTCTCTCC	
SLC35A3	AATGCAATAGTGTTCCAATGTGT/TCTGCAGTCTCTAGCCAGTT	
SOWAHB	AGCTGGCCAGGATAAAGGTT/CGTGCATTACTCCACTCTGC	
MITF (PCR)	AGAGTCTGAAGCGAGAGCATTG/AGTCCACGGATGCTTTTAAAATG (biotinylated)/	Allele quantification using pyrosequencing
MITF (Sequencing)	CAATCACAACTTGATTG (sequencing)	
COL2A1-BD2 (PCR)	TGGTCAGAGGGGCATCGT/GGCGTCCCCACTTACCGA (biotinylated)	
COL2A1-BD2 (Sequencing)	GGTGAGCGAGGATTC	

Supplementary Table 12: Information on the haplotype test for determining MC1R alleles in Holstein cattle

Haplotype	MC1R allele	Frequency in percent
AAAGGGGGAAAAAAGGAGAAGGGACGGGGA	$MC1R^{D}$	12.1
AAAGGGGGAAAAAAGGAGAAGGGACGAAAC	$MC1R^{D}$	10.6
GAAAGGGGAAAAAAGGGAGAAAGGAGGGGC	$MC1R^{D}$	10.1
AAAAGGGGAAAAAAGGGGGAAAGGAGGAAC	$MC1R^{D}$	6.8
GAAGGGGGAAAAAAGAAGAAGGGACGAGGC	$MC1R^{D}$	5.7
GGGAGGGAAAAAAAGGGGGAGGGGCGGGGC	$MC1R^{D}$	5.3
AAAGGAGGGGAAAAAAGAGAGGAGAGGGGA	$MC1R^{D}$	4.7
GAAAAGGGAAAAAAGGGAGAGGGACGAAAC	$MC1R^{D}$	4.3
AAAGGGGGAAAAAAGGAGAAGGGGAGGGGC	$MC1R^{D}$	3.8
GGGGGGGAAAGGCAGAGGAAAAAACGAAAC	$MC1R^{e}$	3.2
GAAGGGGGAAAAAAGGGGGAGGGACGAAAC	$MC1R^{D}$	3.0
AAAGGAGGGGAAAAAAGAGAGGAGAGAAAC	$MC1R^{D}$	2.8
GGGGAAAGGGAAAAGGGAGAGAGACGAAAC	$MC1R^{D}$	1.8
AAAGGAGGGGAAAAAAGAGAGGAACAGAAC	$MC1R^{D}$	1.6
GAAAGGAGGGAAAAGGGAGAGGAGCAAAAC	$MC1R^{D}$	1.6
AAAGGAGGGGAAAAGAGAGAGGAGAGGGGC	$MC1R^{D}$	1.4
GAAAGGGAAAAAAAGAGAGAAAAACGAAAC	$MC1R^{D}$	1.4
GGGGAGGGAAAAAAGGGAGAGGGGAAGAAC	$MC1R^{e}$	1.3
GGGAGGGAAAAAAAGAAGAAAAAACGAAAC	$MC1R^{D}$	1.3
AGGGGGGAAAAAAAGAGAGAAAGGAGGGAC	$MC1R^{+}$	1.0
GAAGGGGGAAAAAAGGGAGAAAGACGGGGC	$MC1R^{D}$	0.8
GAAGGGGGAAAAAAGGGGGAGGAACAGAAC	$MC1R^{D}$	0.7
AGGGAAGGAAAAAAGGAGGAGGAGAGAAAC	$MC1R^{D}$	0.6
AGGGGGGGAAAAAAGGAGAAGGGACGAAAC	$MC1R^{D}$	0.6
GAAGGGAGGGAAAAGGGAGAAAGGAAAGGA	$MC1R^{D}$	0.6
GGGAGGGAAAAAAAGAGAGAAAGACGGAAC	$MC1R^{D}$	0.5

Details on MC1R alleles associated with haplotypes of 30 consecutive SNP from the Illumina BovineSNP50 Beadchip ranging from marker ARS-BFGL-NGS-23632 to marker ARS-BFGL-NGS-38398 (position 13,974,114 bp to 15,931,700 bp on chromosome 18). Alleles for each marker are presented in the“TOP“ format. Haplotypes with a frequency of less than 0.5 percent in 231,115 Holstein cattle genotyped for genomic selection are not presented. This haplotype test was developped in 2011 by Capitan *et al.* (unpublished data) using Illumina BovineSNP50 genotypes from 2317 animals that had been also genotyped for $MC1R^{e}$ and $MC1R^{D}$ mutations. Haplotypes that are neither associated with $MC1R^{e}$ nor with $MC1R^{D}$ mutations are by default considered as associated with allele $MC1R^{+}$.

Supplementary Note 1: Details on animals, phenotypes and sample collection

Tietz syndrome in bovine (Glass-eyed albino; GEA)

From October 2010 to August 2014, we were able to progressively collect samples from a total of nine cases (eight females and one male) which comprised direct and indirect descendants across five generations of the first mutant heifer which was born in 1994 (pedigree available in **Supplementary Fig. 11**). According to the breeder and to our observations, since 2010, there was no distortion of the sex ratio among cases at birth. The limited number of male versus female cases available for sampling is the consequence of the sale of male calves at 3 weeks of age within the dairy system. One of these animals was bought and raised in the INRA Experimental farm at Le Pin-au-Haras under normal husbandry conditions. Deafness was evidenced by lack of response to various auditory stimuli (hand clapping, whistling, clanking of steel bars), and since its development and behavior appeared normal, no further investigations were performed. Its semen was collected on the farm using the normal procedure and deposited in the French national Cryobank for conservation purpose. The animal was finally slaughtered at 18 months and the eye globes, inner ears and different regions of the skin were sampled *post mortem* in the slaughterhouse for histological analyses. At the same time, samples from control Holstein animals were also collected. In addition, ear biopsies from revertant patches and surrounding unpigmented area were progressively obtained from 3 affected animals. In total DNA samples from eight out of the nine cases were available at the time of the genetic study. We were also able to obtain DNA samples from four related female controls (different degrees of maternal cousins), as well as phased Illumina BovineSNP50 Beadchip genotyping data from the wild type sires of the case and controls which had all been previously genotyped together with tens to thousands of their wild type progeny for genomic selection. Finally, DNA and phased Illumina BovineSNP50 Beadchip genotyping data from the sire, maternal grand sire and maternal grand dam's sire of the primo-mutant heifer were also available.

Dominant Red (DR)

Dominant Red also called Variant Red (VR) is distinct from the traditional recessive red allele of the Melanocortin-1 receptor gene ($MC1R^{e}$) found in Holsteins[66]. It emerged in 1980 with the birth of Surinam Sheik Rosabel (HOCANF3541221), a red mutant heifer ($MC1R^{D}/^{D}$ & $DR^{DR/+}$) from parents which were homozygous for the dominant black allele ($MC1R^{D}/^{D}$ & $DR^{+/+}$) and displayed a black coat color. Segregation analysis of the descendants of Rosabel confirmed the dominant inheritance of this phenotype[72]. Because of the emphasis on breeding for red animals in Holstein, DR was favorably selected in North America and more recently in Europe. Ear skin biopsies were sampled on seven dominant red animals, two recessive red and two dominant black, aged between one and 18 months old, which were also genotyped for genomic selection. Hair samples from one dominant red, one recessive red and one dominant black bull, all aged 18 months were plucked on the flank.

CHARGE syndrome in bovine (Novel Neurochristopathy; NC)

In the fall of 2012, the breeding company Umotest reported numerous cases of malformed calves in the progeny of the Montbeliarde bull Etsar (MONFRAM002528725202) which itself intriguingly showed stunted growth. The breeding career of the bull was stopped and samples from four affected animals were sent to the French National observatory of bovine genetic defects. The genome of one of them was sequenced and the causative mutation identified in November 2013. It was only at this stage that the unique scientific interest of this pedigree was revealed. National databases were mined to identify all the offspring of Etsar (n=1058, 543 males and 515 females) and their breeders (n=879). The latter were made aware of the genetic defect and asked a series of questions about the phenotype and behavior of their animals.

To characterize the phenotypes associated with CHARGE syndrome in cattle, we started a systematic examination and sampling of all females still alive at that time. Among them 109 were examined by artificial insemination technicians and 74 by veterinarians.

During the examination of anamnesis records, we focused on age, sex, physiological stage and any problems which could have occurred in the lifetime, especially during the first days of life. Owners were questioned about the ability of their animals to swallow, eat and drink normally.

We then conducted a complete clinical examination. Body condition was scored visually using a 9-point scale (where 1 is emaciated and 9 is overfat) and weight was recorded using a weight measuring tape. Facial symmetry, ear symmetry and shape, tongue size and mobility, and palate integrity were checked. The presence of extra loose ribs was evaluated by palpation. The general behavior, head posture and balance of the animals as well as their posture and gait while walking, trotting and turning quickly in both directions were visually evaluated. In case of ataxia, putative changes in the intensity of the signs were checked while walking on a sloping floor. The functional integrity of cranial nerves I, II, III, IV, VI, VII were evaluated more specifically: sense of smell was tested using a swab impregnated of alcohol; sight was assessed through menace response and pupillary light reflexes (direct and indirect, with a pen torch); hearing was evaluated in response to a loud noise

(clapping); and position of the eyeballs and movements of globes were checked while moving the head, especially up. Pupil size and symmetry were evaluated using a bright pen torch. Disc and retina were inspected by veterinarians (only) using an ophthalmoscope. Ultrasonography of the genital tract was performed by veterinarians on heifers older than 15 months using an ESAOTE Tringa linear VET ®, probe 5-7.5 Mhz machine. Finally, a stethoscope was used to detect abnormal heart sounds and potential heart malformations.
Three heifers aged between 1.5 and two years old presenting with a severe heart murmur were echocardiographied at VetAgro Sup using an ALOKA Alpha 10®, probe 2.5-5 MHz machine. Two of them were euthanized with the agreement of the breeders because of poor health condition, and necropsied. Finally, an additional 1.5-year old heifer and Etsar itself (aged four years) were also examined after slaughter in a conventional slaughterhouse.

In addition we analyzed a number of phenotypes routinely collected in test stations and dairy farms.
To characterize the morphology of Etsar, data from 467 young bulls recorded in the test station of Ceyzeriat (France) were used. Data included morphology scores for 14 traits, birth weight, and four to eight live weights recorded between 50 and 300 days. For morphology traits, data were adjusted for batch and age with GLM procedure (SAS) and Etsar's percentile was identified. For growth, weights at 13 standardized ages from birth to 300 days (by 25-day classes) were obtained by fitting a polynomial growth curve. Then these weights were adjusted for batch effect and, as before, Etsar's percentile was identified.
To evaluate the reproductive performance of Etsar, the volume and concentration of the ejaculate as well as the percentage of live sperm and sperm motility after thawing were analyzed for 79 young bulls collected on average 14 times between 12 and 16 months of age in the test station of Ceyzeriat. Data were adjusted for batch and age with GLM procedure (SAS). Then the performances of Etsar were compared with the performances of the other bulls using a Student t-test. The average performance of each animal was also calculated for each trait and the rank of Etsar determined. In addition, we analyzed the estimated sire conception rate of 2747 bulls which were calculated in the framework of the routine genetic evaluation of the female fertility in the Montbéliarde breed and which are adjusted for a number of effects (herd, date, inseminator, age and calving rank of the cow, and, percentage of inbreeding of the mating).
The birth weight, birth condition score and daily milk production of Etsar's daughters and their herdmates born in 2012 were also extracted from the French national database (Système National d'Information Génétique des Bovins) and analyzed. Different groups were constituted and the distributions of their performances or their average performances were compared using a Pearson Chi-square or a Student t-test, respectively. Finally mortality rate per sex and per month was also calculated among the progeny of Etsar and compared to the Montbéliarde breed reference (Michel Douguet, Institut de l'Elevage, pers. comm.)
For mapping the NC locus, 42 affected and 71 unaffected animals among the 183 females examined by artificial insemination technicians or veterinarians, were genotyped with the Illumina BovineSNP50 Beadchip or with the Illumina custom EuroG10K Beadchip. DNA and genotyping data were also available for 31 additional offspring of Etsar (2 females and 29 males) which had been genotyped in their first week of life for genomic selection and which were not available for clinical examination. These were genotyped for the causative mutation to distinguish the cases and controls. Then, the life duration of the cases, their cause of death and testimonies from the breeders were taken into account to classify them as mildly or severely affected. In total 49 mildly affected, 7 severely affected and 89 control half sibs were used to map modifier loci. Finally DNA and Illumina BovineSNP50 Beadchip genotyping data from Etsar's dam and sire were also available for verifying the *de novo* nature of the CHD7 frameshift mutation.

Osteogenesis imperfecta type 2 in bovine (OI)
Austrian farmers reported numerous calves with multiple bone fractures at birth and a high perinatal mortality among the descendants of the Fleckvieh bull "Halvar PP" (SIMAUTM000070213719). Of 442 paternal halfsibs, 107 (24.2%; 43 males, 64 females) were stillborn or died within 48 hours after birth, which is five times higher than the average perinatal mortality in Fleckvieh cattle[73]. Another 33 calves perished during rearing. Affected calves were born after normal gestation length. The clinical and pathological findings were compatible with a diagnosis of bovine osteogenesis imperfecta (OI) type 2. There were no phenotypic abnormalities detected in the sire and similar disorders had not been reported in the Fleckvieh cattle breed before. Such findings were compatible with the presence of an autosomal dominant de novo mutation for which the sire would be germline mosaic. Two affected calves were clinically and pathologically examined. The sire, seven affected and twenty unaffected halfsibs, as well as two dams were available for genetic studies.

Achondrogenesis type 2 in bovine: (Bulldog calf syndrome; BD1, BD2 and BD3)
Bulldog calves were reported in three unrelated pedigrees and referred as BD1, BD2 and BD3.

BD1: in total, five affected calves (four males, one female), which were all stillborn were reported among the 114 descendants of the Charolais bull “Farceur” (CHAFRAM007121570439) and Salers cows. Among them, only the last case, a male, was available for necropsy. Frozen brain samples from another male case, which had been previously sent to the veterinary laboratory of the Loire department for epidemiological tests, were also available for DNA extraction. Finally, the sire, the dams of these two cases, six paternal halfsibs and their dams, which all presented a normal phenotype, were sampled for genetic studies.
BD2: German cattle breeders reported a large number of stillborn calves with lethal congenital malformations among 275 descendants of the Holstein bull “Energy P” (HOLCANM000011696813). One affected animal was subjected to necropsy. The sire, ten affected and fifty-eight unaffected halfsibs were available for genetic studies.
BD3: one isolated case of bulldog calf was reported in a Swiss Holstein herd. According to the breeding company no similar affected offspring was noticed among the numerous progeny of its AI sire. The dam was a primiparous cow which was not closely related to the sire. The calf was subjected to necropsy and the trio was available for genetic studies.
In addition to the routine examination, particular attention was paid to the skeleton. BD calves were radiographed using different instruments. Longitudinal sections of bones were also performed on specimens frozen at - 20°C and histological analyses were conducted on BD3.

Recessive Anhidrotic Ectodermal Dysplasia in bovine (AED)
Shortly after the identification of a frameshift mutation in *EDAR* in the genome of the Charolais bull “Invincible” (CHAFRAM001893105503), French veterinarians were requested to submit AED calves for clinical examination via an announcement made on the Vetofocus website. National databases were mined to confirm that Invincible was present on both sides of the pedigree of the ten affected calves that were reported to us. Among them three were already dead at the time of the study. The seven remaining calves were subjected to visual clinical evaluation on the farm. Particular attention was paid to the oral cavity, the size and repartition of hair, as well as teats, horns and hoofs. Horn growth was evaluated on a young male from one month to its death at five-and-a-half months. A one-month-old affected calf, which was euthanized upon the request of its breeders who had already lost previous affected calves from hypothermia and lung infections, was subject to necropsy examination. Biopsies in different area of the body were sampled for histological analyses. Its skull was radiographed using a GIERTH HF 80 ML Ultra Leicht machine coupled with a PCR Eleva Radiological Image Processing System. Finally the upper molars were extracted from the jaw bone and subjected to visual examination. Seven affected calves, the ten dams and four sires were available for genetic studies.

Supplementary Note 2: A large bovine pedigree provide insights into the variable clinical expression of CHARGE syndrome

The frameshift mutation responsible for a novel Neurocristopathy in a Montbéliarde pedigree is predicted to produce a truncated protein lacking functional domains (CHD7 p.K594AfsX29; Fig. X). In humans, CHD7 haploinsufficiency causes a variable combination of congenital malformations referred as CHARGE syndrome[22,74,75] (MIM: 214800). Clinical examination of the heterozygote bull and 109 out of its 1057 descendants enabled us to retrieve (i) each of the most common symptoms of CHARGE syndrome, (ii) some rare symptoms and (iii) the intra-familial variability observed in humans (**Fig. 4; Supplementary Fig. 12**).

In addition, the mining of phenotypic records which are routinely collected in Artificial Insemination centers enabled us to further characterize this syndrome.

While it was moderately affected in comparison with some of its progeny, the primo-mutant sire Etsar showed (**Fig. 4.e**, **Supplementary Fig. 12.i**) marked growth retardation as compared with a panel of 467 young bulls raised in the same test station. He was in the third percentile in term of body weight (**Supplementary Fig. 12.i**) and in the fifth percentile or below for eight of 14 body measures (**Fig. 4.e**). Etsar did not show evidences of abnormal genital development (e.g. hypospadia which is sometimes reported in male CHARGE patients). It displayed normal fertility (effect of -1% compared to the average sire conception rate; 32th percentile out of 2747 AI bulls) and normal or improved performances for semen production traits with the exception of sperm–volume which can be correlated with a general reduction of its body size (**Supplementary Tab. 6**).

A high postnatal mortality was observed among the descendants of Etsar in both sexes and especially within the first month of life (**Supplementary Tab. 7**). We estimated that as much as 40.5% of males and 32.5% of females carrying the *CHD7* frameshift mutation died during this period. Based on retrospective breeders' and veterinarians' testimonies these deaths were mainly due to two factors: (i) an incapacity to suckle by calves presenting with severe clefting of lips and palate and (ii) severe cardiac defects. Their premature death is the major reason why we were able to recruit only a handful of cases among the most severely affected animals for clinical examination.

Interestingly, the analysis of the birth weights of the offspring of Etsar supported a significant reduction of birth weights among carriers of the CHD7 frameshift mutation as compared with the general population (**Supplementary Fig. 13.a**); a result which has never been shown in humans probably because of the heterogeneity of the mutations and of the populations studied. Indeed, among the progeny of Etsar which were available for sampling and genotyping (i.e. comprising wild type progeny and moderately to mildly affected heterozygous carriers), heterozygous carriers showed a significant reduction in birth weight as compared with the Montbéliarde population ($p<0.05$) whereas wild type progeny did not. In addition, the progeny of Etsar which died before one month of age (i.e. which comprise a number of severely affected heterozygous carriers of the mutation) showed very significant reduction of birth weight as compared with (i) the general population which died in the same period of age, (ii) the general population in total, and (iii) the wild type progeny. In contrast, calves from the Montbéliarde population which also died within their first month of life did not show significant differences with these two last groups. Taken together these results indicate that heterozygous carriers of the mutation display reduced birth weight and that this reduction seems to be correlated with the severity of the syndrome. Analysis of birth conditions scores did not reveal significant differences between groups (not shown).

Finally it is worth noting that a small number of heterozygous carrier females (n=10) had been kept for reproduction by the breeders. These animals did not show any obvious symptoms and were mistakenly considered as wild type before they were genotyped, even if (i) retrospectively the breeders remembered that they experienced difficulties and required more attention in the beginning of their life and (ii) clinical examination revealed mild signs of ataxia and damage to cranial nerves (e.g. **Supplementary Fig. 12.g**,**h)**. Interestingly these females showed a marked reduction in milk production between 7 and 50 days and 51 to 100 days as compared with their wild type sisters (**Supplementary Fig. 13.b**). The poor average milk production of the heterozygous carriers corresponds to 72.6 % and 65.2 % of the average production of their wild-type sisters on the two periods investigated. Their age at conception (**Supplementary Fig. 13.c**) which can be considered as an indicator of their general growth (since the breeders usually delay the insemination of the heifers which have not reached sufficient body weight) was not significantly different from their wild type sisters. In addition, clinical examination did not reveal sufficient reductions of their body size or signs of metabolic defects that could explain such a reduction in their milk production. This phenotype has most probably a hormonal etiology like reproductive dysfunction frequently observed in *CHD7*+/- humans and mouse[76,77] which has been attributed to abnormal development and maintenance of GnRH neurons[77]. In this case poor lactation would not be caused

by a reduction of the pulsatile GnRH/LH secretion, but on the contrary by an abnormal regulation of the secretion of these hormones which are normally inhibited during lactation[78].
In conclusion this large pedigree combined with extensive phenotype recording enabled us to explore aspects of the CHARGE syndrome that are difficult to investigable in humans. The analysis of bovine fetuses also offers promising prospects because of their size, and ease of production at key development stages using cull cows. To enable such studies in the future, the semen of Etsar has been conserved and will be made available to other research groups upon request.

Supplementary Data 1: Results of differential expression analysis comparing dominant red and dominant black skin samples according to Dorshorst *et al.* [41].
A threshold of FDR<0.05 was used to retain significant expression changes between dominant red and dominant black skin samples. Transcript annotation was updated using information from the last release (88) of from the UCSC Genome Browser (see text for details).

REFERENCES

1. Simmons, D. The Use of Animal Models in Studying Genetic Disease: Transgenesis and Induced Mutation. *Nature Education* **1**, 70 (2008).
2. Rodríguez-Seguí, S., Akerman, I. & Ferrer, J. GATA believe it: new essential regulators of pancreas development. *J. Clin. Invest.* **122**, 3469–3471 (2012).
3. Andersson, L. Molecular consequences of animal breeding. *Curr. Opin. Genet. Dev.* **23**, 295-301 (2013).
4. Daetwyler, H.D. *et al.* Whole-genome sequencing of 234 bulls facilitates mapping of monogenic and complex traits in cattle. *Nat. Genet.* **46**, 858-865 (2014).
5. Li, H. *et al.* The Sequence Alignment/Map format and SAMtools. *Bioinformatics* **25**, 2078–2079 (2009).
6. McLaren, W. *et al.* Deriving the consequences of genomic variants with the Ensembl API and SNP Effect Predictor. *Bioinformatics* **26**, 2069-2070 (2010).
7. Ye, K., Schulz, M.H., Long, Q., Apweiler, R. & Ning, Z. Pindel: a pattern growth approach to detect break points of large deletions and medium sized insertions from paired-end short reads. *Bioinformatics* **25**, 2865–2871 (2009).
8. Rausch, T., *et al.* DELLY: structural variant discovery by integrated paired-end and split-read analysis. *Bioinformatics* **28**, i333-i339 (2012).
9. Thorvaldsdóttir, H. *et al.* Integrative Genomics Viewer (IGV): high-performance genomics data visualization and exploration. *Brief Bioinform.* **14**, 178-192 (2013).
10. Vissing, H. *et al.* Glycine to serine substitution in the triple helical domain of pro-alpha 1 (II) collagen results in a lethal perinatal form of short-limbed dwarfism. *J. Biol. Chem.* **264**, 18265-18267 (1989).
11. Hanley, K.P. *et al.* Ectopic SOX9 mediates extracellular matrix deposition characteristic of organ fibrosis. *J. Biol. Chem.* **283**, 14063-14071 (2008).
12. Shi, S., Kirk, M. & Kahn, A.J. The role of type I collagen in the regulation of the osteoblast phenotype. *J. Bone Miner. Res.* **11**, 1139-1145 (1996).
13. Levy, C., Khaled, M. & Fisher, D.E. MITF: master regulator of melanocyte development and melanoma oncogene. *Trends Mol. Med.* **12**, 406-414 (2006).
14. Takebayashi, K. *et al.* The recessive phenotype displayed by a dominant negative microphthalmia-associated transcription factor mutant is a result of impaired nucleation potential. *Mol. Cell. Biol.* **16**, 1203-1211 (1996).
15. Hodgkinson, C.A. *et al.* Mutations at the mouse microphthalmia locus are associated with defects in a gene encoding a novel basic-helix-loop-helix-zipper protein. *Cell* **74**, 395-404 (1993).
16. Steingrímsson, E. *et al.* Molecular basis of mouse microphthalmia (mi) mutations helps explain their developmental and phenotypic consequences. *Nat. Genet.* **8**, 256-263 (1994).
17. Hansdottir, A.G. *et al.* The novel mouse microphthalmia mutations Mitfmi-enu5 and Mitfmi-bcc2 produce dominant negative Mitf proteins. *Genomics* **83**, 932-935 (2004).
18. Philipp, U. *et al.* A MITF mutation associated with a dominant white phenotype and bilateral deafness in German Fleckvieh cattle. *PLoS One* **6**, e28857 (2011).
19. Hauswirth, R. *et al.* Mutations in MITF and PAX3 cause "splashed white" and other white spotting phenotypes in horses. *PLoS Genet.* **8**, e1002653 (2012).
20. Léger, S. *et al.* Novel and recurrent non-truncating mutations of the MITF basic domain: genotypic and phenotypic variations in Waardenburg and Tietz syndromes. *Eur. J. Hum. Genet.* **20**, 584-587 (2012).
21. Bajpai, R. *et al.* CHD7 cooperates with PBAF to control multipotent neural crest formation. *Nature* **463**, 958–962 (2010).
22. Pagon, R.A., Graham, J.M. Jr, Zonana, J. & Yong, S.L. Coloboma, congenital heart disease, and choanal atresia with multiple anomalies: CHARGE association. *J. Pediatr.* **99**, 223-227 (1981).
23. Blake, K.D. *et al.* CHARGE association: an update and review for the primary pediatrician. *Clin. Pediatr. (Phila.)* **37**, 159–173 (1998).
24. Amiel, J. *et al.* Temporal bone anomaly proposed as a major criteria for diagnosis of CHARGE syndrome. *Am. J. Med Genet.* **99**, 124–127 (2001).
25. Verloes, A. Updated diagnostic criteria for CHARGE syndrome: a proposal. *Am. J. Med. Genet.* **133A**, 306-308 (2005).
26. Jongmans, M.C. *et al.* CHARGE syndrome: the phenotypic spectrum of mutations in the CHD7 gene. *J. Med. Genet.* **43**, 306–314 (2006).
27. Lalani, S.R. *et al.* Spectrum of CHD7 mutations in 110 individuals with CHARGE syndrome and genotype-phenotype correlation. *Am. J. Hum. Genet.* **78**, 303–314 (2006).

28. Vuorela, P. *et al.* Molecular analysis of the CHD7 gene in CHARGE syndrome: identification of 22 novel mutations and evidence for a low contribution of large CHD7 deletions. *Genet. Med.* **9**, 690-694 (2007).
29. Delahaye, A. *et al.* Familial CHARGE syndrome because of CHD7 mutation: clinical intra- and interfamilial variability. *Clin. Genet.* **72**, 112-121 (2007).
30. Vuorela, P.E. *et al.* A familial CHARGE syndrome with a CHD7 nonsense mutation and new clinical features. *Clin. Dysmorphol.* **17**, 249-253 (2008).
31. Hughes, S.S., Welsh, H.I., Safina, N.P., Bejaoui, K. & Ardinger, H.H. Family history and clefting as major criteria for CHARGE syndrome. *Am. J. Med. Genet.* **164A**, 48-53 (2014).
32. Lachmann, A. *et al.* ChEA: transcription factor regulation inferred from integrating genome-wide ChIP-X experiments. *Bioinformatics* **26**, 2438-2444 (2010).
33. Rouillard, A.D. *et al.* The harmonizome: a collection of processed datasets gathered to serve and mine knowledge about genes and proteins. *Database* (2016).
34. Radice, G.L. *et al.* Developmental defects in mouse embryos lacking N-cadherin. *Dev. Biol.* **181**, 64-78 (1997).
35. Kadowaki, M. *et al.* N-cadherin mediates cortical organization in the mouse brain. *Dev. Biol.* 304, 22-33 (2007).
36. Li, J. *et al.* N-cadherin haploinsufficiency affects cardiac gap junctions and arrhythmic susceptibility. *J Mol. Cell. Cardiol.* **44**, 597-606 (2008).
37. Letourneur, F. *et al.* Coatomer is essential for retrieval of dilysine-tagged proteins to the endoplasmic reticulum. *Cell* **79**, 1199-1207 (1994).
38. Jackson, L.P. *et al.* Molecular basis for recognition of dilysine trafficking motifs by COPI. *Dev. Cell* **23**, 1255-1262 (2012).
39. Coutinho, P. *et al.* Differential requirements for COPI transport during vertebrate early development. *Dev. Cell* **7**, 547-558 (2004).
40. Xu, X. *et al.* Mutation in archain 1, a subunit of COPI coatomer complex, causes diluted coat color and Purkinje cell degeneration. *PLoS Genet.* **6**, e1000956 (2010).
41. Dorshorst, B. *et al.* Dominant Red coat color in Holstein cattle is associated with a missense mutation in the Coatomer Protein Complex, subunit Alpha (COPA) gene. *PLoS One* **10**, e0128969 (2015).
42. Capitan, A. *et al.* Rapid discovery of mutations responsible for sporadic dominant genetic defects in livestock using genome sequence data: Enhancing the value of farm animals as model species. *Proc. 10th World Congr. Genet. Appl. Livest. Sci.* **Commun. 182** (2014). Oral presentation available at [https://asas.confex.com/asas/WCGALP14/flvgateway.cgi/id/5159?recordingid=5159]
43. Watkin, L.B. et al. COPA mutations impair ER-Golgi transport and cause hereditary autoimmune-mediated lung disease and arthritis. Nat. Genet. 47, 654-660 (2015).
44. Vece, T.J. *et al.* Copa syndrome: a novel autosomal dominant immune dysregulatory disease. *J. Clin. Immunol.* **36**, 377-387 (2016).
45. Northrop, R.B., Connor, A.N. In: Neuman, M.R. (ed.) Introduction to Molecular Biology, Genomics and Proteomics for Biomedical Engineers, p. 37. CRC Press Taylor and Francis Group, Boca Raton (2009)
46. Chen, E.Y. *et al.* Enrichr: interactive and collaborative HTML5 gene list enrichment analysis tool. *BMC Bioinformatics* **14**, 128 (2013).
47. Kuleshov, M.V. *et al.* Enrichr: a comprehensive gene set enrichment analysis web server 2016 update. *Nucleic Acids Res.* **44**, W90–W97 (2016).
48. Baguma-Nibasheka, M. & Kablar, B. Pulmonary hypoplasia in the connective tissue growth factor (Ctgf) null mouse. *Dev. Dyn.* **237**, 485-493 (2008).
49. Gründer *et al.* Nuclear factor I-B (Nfib) deficient mice have severe lung hypoplasia. *Mech. Dev.* **112**, 69-77 (2002).
50. Ramirez, M.I. *et al.* T1alpha, a lung type I cell differentiation gene, is required for normal lung cell proliferation and alveolus formation at birth. *Dev. Biol.* **256**, 61-72 (2003).
51. Saadoun, D., Terrier, B. & Cacoub, P. Interleukin-25: key regulator of inflammatory and autoimmune diseases. *Curr. Pharm. Des.* **17**, 3781-3785 (2011).
52. Molofsky, A.B., Savage, A.K. & Locksley, R.M. Interleukin-33 in tissue homeostasis, injury, and inflammation. *Immunity* **42**, 1005-1019 (2015).
53. Parks, W.C., Wilson, C.L. & López-Boado, Y.S. Matrix metalloproteinases as modulators of inflammation and innate immunity. *Nat. Rev. Immunol.* **4**, 617-629 (2004).
54. Burkly, L. *et al.* Expression of relB is required for the development of thymic medulla and dendritic cells. *Nature* **373**, 531-536 (1995).

55. Valéro, R. *et al.* A defective NF-kappa B/RelB pathway in autoimmune-prone New Zealand black mice is associated with inefficient expansion of thymocyte and dendritic cells. *J. Immunol.* **169**, 185-192 (2002).
56. Chang, C.Y. *et al.* NFIB is a governor of epithelial-melanocyte stem cell behaviour in a shared niche. *Nature* **495**, 98-102 (2013).
57. Masui, Y. *et al.* A missense mutation in the death domain of EDAR abolishes the interaction with EDARADD and underlies hypohidrotic ectodermal dysplasia. *Dermatology* **223,** 74-79 (2011).
58. Sadier, A., Viriot, L., Pantalacci, S. & Laudet, V. The ectodysplasin pathway: from diseases to adaptations. *Trends Genet.* **30**, 24-31 (2014).
59. Li, H. & Durbin, R. Fast and accurate short read alignment with Burrows-Wheeler Transform. *Bioinformatics* **25**, 1754-1760 (2009).
60. Boichard, D. *et al.* Genomic Selection in French Dairy Cattle. *Anim. Prod. Sci.* **52**, 115-120 (2012).
61. Sargolzaei, M., Chesnais, J.P. & Schenkel, F.S. A new approach for efficient genotype imputation using information from relatives. *BMC Genomics* **15**, 478 (2014).
62. Weckx, S. *et al.* NovoSNP, a novel computational tool for sequence variation discovery. *Genome Res.* **15**, 436–442 (2005).
63. Lander, E.S. & Botstein, D. Homozygosity mapping: a way to map human recessive traits with the DNA of inbred children. *Science* **236**, 1567-1570 (1987).
64. Charlier, C. *et al.* Highly effective SNP-based association mapping and management of recessive defects in livestock. *Nat. Genet.* **40**, 449-454 (2008).
65. Boichard, D. Pedig: a Fortran package for pedigree analysis suited to large populations. 7th World Congress on Genetics Applied to Livestock Production, Montpellier, August 19-23 2002, paper 28-13 (2002).
66. Joerg, H., Fries, H.R., Meijerink, E. & Stranzinger, G.F. Red coat color in Holstein cattle is associated with a deletion in the MSHR gene. *Mamm. Genome* **7**, 317-318 (1996).
67. Grill, C. *et al.* MITF mutations associated with pigment deficiency syndromes and melanoma have different effects on protein function. *Hum. Mol. Genet.* **22**, 4357-4367 (2013).
68. Guibert, S., Girardot, M., Leveziel, H., Julien, R. & Oulmouden, A. Pheomelanin coat colour dilution in French cattle breeds is not correlated with the TYR, TYRP1 and DCT transcription levels. *Pigment Cell Res.* **17**, 337-345 (2004).
69. Girardot, M. *et al.* The insertion of a full-length Bos taurus LINE element is responsible for a transcriptional deregulation of the Normande Agouti gene. *Pigment Cell Res.* **19**, 346-355 (2006).
70. Thomsen, B. *et al.* A missense mutation in the bovine SLC35A3 gene, encoding a UDP-N-acetylglucosamine transporter, causes complex vertebral malformation. *Genome Res.* **16**, 97-105 (2006).
71. Sun, M. *et al.* Multiplex chromosomal exome sequencing accelerates identification of ENU-induced mutations in the mouse. *G3* **2**, 143–150 (2012).
72. Leduc, M. The Various Mechanisms of Red Colour Transmission in the Holstein Breed. *Holstein Journal* **May**, 17–19 (2006).
73. Pausch, H. *et al.* Homozygous haplotype deficiency reveals deleterious mutations compromising reproductive and rearing success in cattle. *BMC Genomics* **16**, 312 (2015).
74. Hall, B.D. Choanal atresia and associated multiple anomalies. *J. Pediatr.* **95,** 395-398 (1979).
75. Hittner, H.M., Hirsch, N.J., Kreh, G.M. & Rudolph, A.J. Colobomatous microphthalmia, heart disease, hearing loss, and mental retardation--a syndrome. *J. Pediatr. Ophthalmol. Strabismus.* **16**, 122-128 (1979).
76. Dauber, A., Hirschhorn, J.N., Picker, J., Maher, T.A. & Milunsky, A. Delayed puberty due to a novel mutation in CHD7 causing CHARGE syndrome. *Pediatrics* **126**, e1594-1598 (2010).
77. Layman, W.S., Hurd, E.A. & Martin, D.M. Reproductive dysfunction and decreased GnRH neurogenesis in a mouse model of CHARGE syndrome. *Hum. Mol. Genet.* **20**, 3138-3150 (2011).
78. Smith, M.S., True, C. & Grove, K.L. The neuroendocrine basis of lactation-induced suppression of GnRH: role of kisspeptin and leptin. *Brain Res.* **1364**, 139-152 (2010).
